# Broadly Applicable Synthesis of Arylated Dithieno[3,2‐*b*:2′,3′‐*d*]pyrroles as Building Blocks for Organic Electronic Materials

**DOI:** 10.1002/chem.202101478

**Published:** 2021-07-29

**Authors:** Astrid Vogt, Fabian Schwer, Sebastian Förtsch, Christoph Lorenz, Elena Mena‐Osteritz, Anna Aubele, Teresa Kraus, Peter Bäuerle

**Affiliations:** ^1^ Institute of Organic Chemistry II and Advanced Materials University of Ulm Albert-Einstein-Allee 11 89081 Ulm Germany; ^2^ IFF August-Wolff-Straße 13 29699 Walsrode Germany

**Keywords:** cross-coupling, dithienopyrrole, heterocycles, organic solar cell, structure-property relationship

## Abstract

A novel and versatile method for the N‐arylation of dithieno[3,2‐*b*:2′,3′‐*d*]pyrrole (DTP) is presented. By Pd‐ or Cu‐catalyzed coupling a variety of arenes and acenes were directly attached at the DTP−nitrogen yielding a variety of functionalized DTPs. Investigations on optical and redox properties led to valuable structure‐property relationships, which were corroborated by quantum chemical calculations. Further functionalization and elongation of the conjugation of an acceptor‐substituted DTP was elaborated to result in complex cruciform‐type donor−acceptor oligomers, which were investigated and implemented in single material organic solar cells.

## Graphical abstract

**A versatile method** is presented for the direct N‐arylation of dithieno[3,2‐*b*:2′,3′‐*d*]pyrrole (DTP) providing a variety of functionalized derivatives. Three catalyst systems were explored, whereby Pd_2_dba_3_/*t*Bu_3_P showed broad applicability with good yields throughout. Investigations on optical and redox properties led to valuable structure‐property relationships. Further functionalization of an acceptor‐substituted DTP yielded cruciform‐type donor−acceptor oligomers, which were implemented into single material organic solar cells.

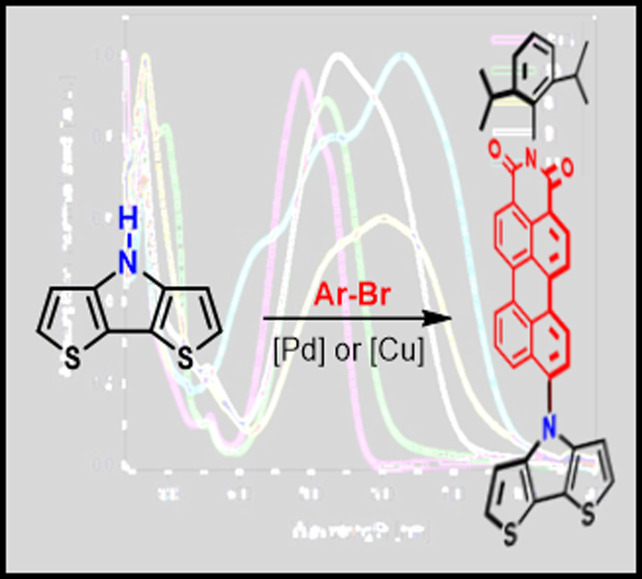

## Introduction

**Scheme 1:**
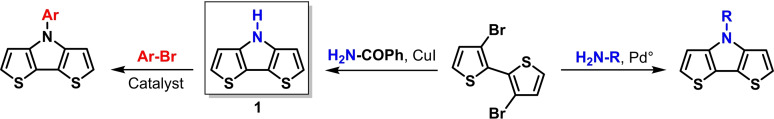
Common synthesis of alkylated and arylated dithienopyrroles (DTP) by reacting 3,3′‐dibromo‐2,2′‐bithiophene with amines (right). New ‘inverted’ synthesis of arylated DTPs via *H*−DTP **1** and reaction with aryl bromides (left, this work).

Dithieno[3,2‐*b*:2′,3′‐*d*]pyrroles (DTP) represent an interesting multi‐functional and electron‐rich building block for organic semiconductors, for example, as donor unit in low bandgap donor−acceptor polymers^[1]^ or conjugated oligomers,[^2^, ^3^] which are applied in organic electronic devices, in particular in organic solar cells.^[4]^ Due to the reactive α‐positions, DTPs can be oxidatively polymerized into conductive polymers,^[5]^ which for example can be applied as electrode material in rechargeable batteries.[^6^, ^7^] The frequently used *N*‐alkyl and *N*‐aryl DTPs are effectively synthesized via Pd‐catalyzed Buchwald‐Hartwig amination/cyclization of 3,3′‐dibromo‐2,2′‐bithiophene precursors and alkyl or aryl amines to form the central pyrrole ring (Scheme 1).[^1^, ^8^] In this respect, some N‐phenylated DTPs have been described, whereby only very few examples of DTPs bearing larger aryls or polycyclic aromatic hydrocarbons such as truxene^[9]^ or 1‐naphthaline^[10]^ are known.

We have recently disclosed a straightforward and safe method for the large‐scale synthesis of basic 4*H*−dithieno[3,2‐*b*:2′,3′‐*d*]pyrrole **1** (*H*−DTP).^[11]^ This progress now allowed us to test and optimize the synthesis of a variety of arylated DTPs comprising larger aryls and acenes by Pd‐ or Cu‐catalyzed coupling of *H*−DTP **1** and well available aryl and acene halides **2 a**–**k** (Scheme 1). The resulting N‐arylated and N‐acene‐substituted DTPs represent interesting building blocks for functional materials applicable in organic electronics.

## Results and Discussion

4*H*−DTP **1** can be seen as electron‐rich analogue of 9*H*−carbazole and intermediate 8*H*−thieno[3,2‐*b*]indole. Direct N‐arylations are well known for carbazole derivatives, which are well represented in natural products or in organic materials. Typical methods include reaction of carbazole with diaryliodonium salts without metal catalysts,^[12]^ with aryl halides and Cu‐catalysis (CuI),^[13]^ or Pd‐catalysis (Pd_2_dba_3_/ligand).^[14]^ Mostly phenyl substituents were coupled, but sterically hindered aryls^[14]^ or heteroaryls[^13a^, ^13c^] could only very rarely be attached. With respect to thienoindoles only one example is known, and Driver et al. described the coupling of the heteroacene and 1‐bromo‐4‐butylbenzene with the catalyst system Pd(OAc)_2_/*t*Bu_3_P. The resulting N‐arylated thienoindole was used as a building block for the preparation of heteroheptacenes with good charge transport properties.^[15]^ As in the case of arylated DTPs, a broad series of corresponding thienoindoles were prepared by Langer et al. by Buchwald‐Hartwig amination of dibrominated phenylthiophene precursors and aryl amines.^[16]^ The only example of a direct N‐arylation of DTP was this year released by Tang et al., who reacted 2‐bromonaphthoquinone and 2‐bromoanthraquinone with *H*−DTP **1** under Pd‐catalysis with (Pd_2_dba_3_/X−Phos) to the corresponding arylated DTPs in 40 % and 47 % yield, respectively.^[17]^

Although *N*‐phenyl DTP **3 a** can be conveniently prepared via amination of 3,3′‐dibromo‐2,2′‐bithiophene and aniline in 84 % yield,[^5^, ^8^] we investigated in initial studies the alternative ‘inverse’ arylation of *H*−DTP **1** as amine precursor. A priori, we discarded diaryliodonium salts as arylation reagents (see above), because the scope of available derivatives is restricted. In order to screen the possible catalyst system and the leaving group of the arylation reagent, *H*−DTP **1** was firstly reacted with various phenyl halides and tosylate as model reagents under Pd(OAc)_2_ catalysis and NaO*t*Bu as base in toluene. A brief screening of the ligand revealed *t*Bu_3_P>*t*BuXPhos>P(*o*‐Tol*)*_3_ and of the substrate Br>I>Cl>OTos (Table S1, Supporting Information). In the following, we therefore used the well available broad scope of aryl bromides as phenylating reagents. Thus, in the reaction of *H*−DTP **1** and bromobenzene **2 a** *Ph*−DTP **3 a** was isolated in 78 % yield with the catalytic system Pd(OAc)_2_/*t*Bu_3_P. The change to Pd_2_dba_3_/*t*Bu_3_P or CuI under microwave‐assisted conditions as catalysts gave slightly higher or the same yield. Having established the frame conditions, we then examined the scope of the arylation of *H*−DTP **1** and further tested additional electronically and sterically different aryl and acene bromides **2 b**–**2 k** with all three catalytic systems. The results are compiled in Table 1.

**Table 1:** Arylation reaction of *H*−DTP **1** with aryl bromides **2 a**–**k** to arylated DTPs **3 a**–**k**.^[a]^

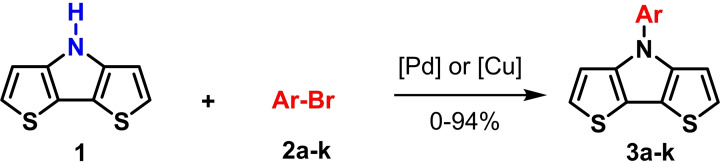							
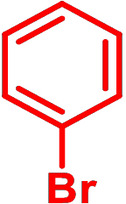	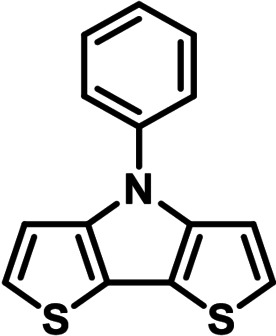	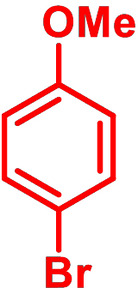	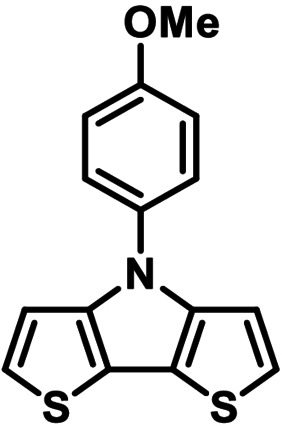	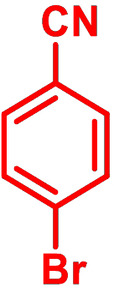	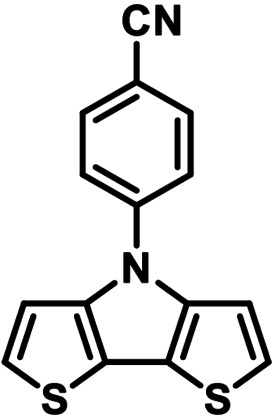	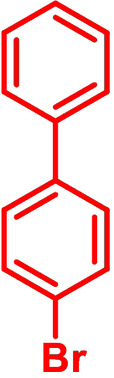	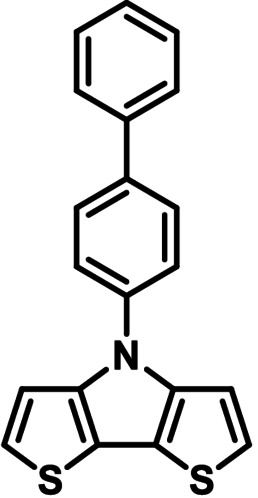
**2 a/3 a**		**2 b/3 b**		**2 c/3 c**		**2 d/3 d**	
(78 %/76 %/76 %)		(54 %/89 %/61 %)		(59 %/85 %/52 %)		(66 %/87 %/69 %)	
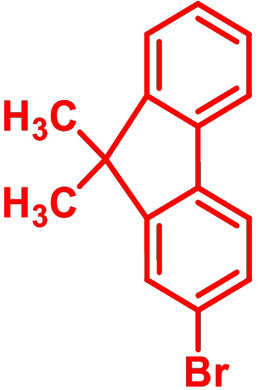	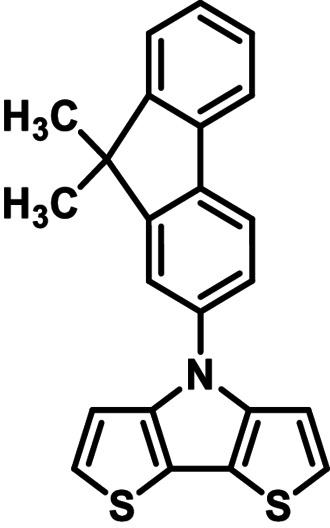	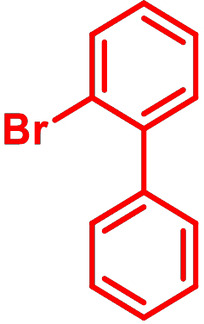	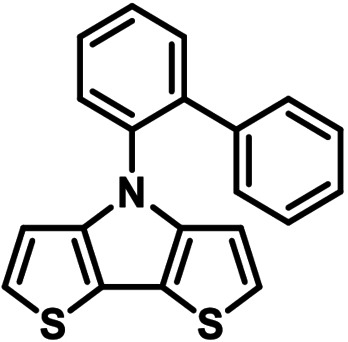	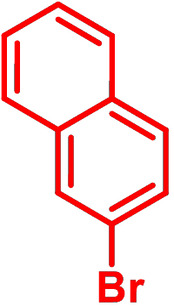	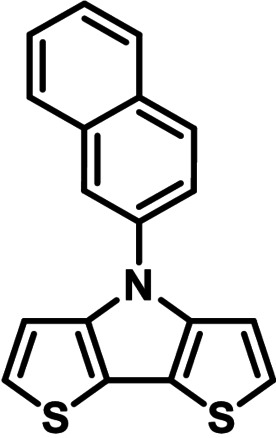	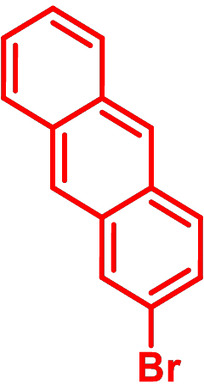	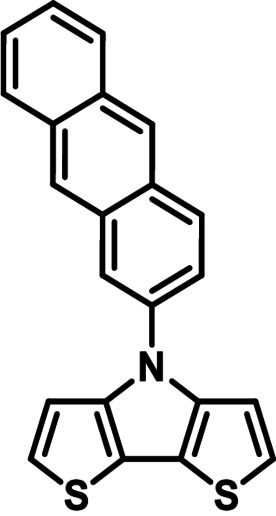
**2 e/3 e**		**2 f/3 f**		**2 g/3 g**		**2 h/3 h**	
(72 %/94 %/72 %)		(0 %/0 %/33 %)		(59 %/89 %/87 %)		(41 %/61 %/45 %)	
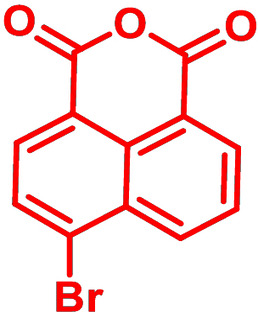	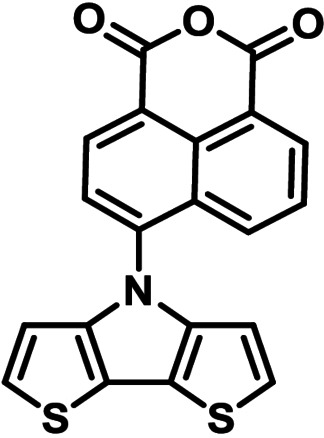	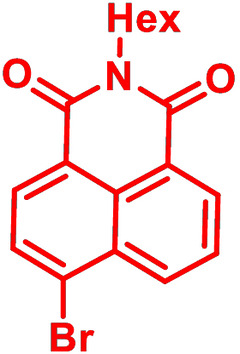	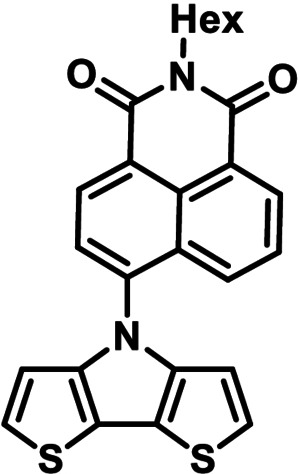	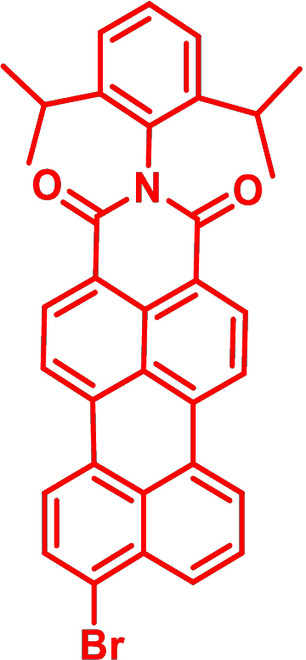	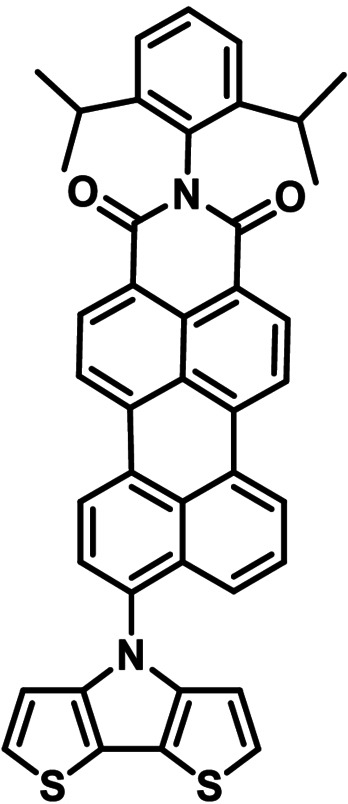		
**2 i/3 i**		**2 j/3 j**		**2 k/3 k**			
(11 %/18 %/0 %)		(64 %/93 %/45 %)		(69 %/59 %/9 %)			

[a] Yields are given as isolated yields of purified substance with Pd(OAc)_2_/*t*Bu_3_P, Pd_2_dba_3_/*t*Bu_3_P, CuI/microwave as catalyst. Detailed conditions are given in the experimental part.

As with bromobenzene **2 a**, the aryl substrates **2 b**–**e** bearing electron‐donating (**2 b**), electron‐withdrawing (**2 c**), and extended conjugated π‐systems (**2 d**–**e**) readily reacted with *H*−DTP **1** to the targeted arylated DTPs **3 b**–**e** in acceptable to good yields (52–94 %) with moderate variations for the three catalysts. In particular, CuI was the only catalyst to promote reaction of *H*−DTP **1** with 2‐bromo‐1,1′‐biphenyl **2 f** and DTP **3 f** could be isolated in lower 33 % yield most probably due to steric hindrance of the biphenyl unit in the catalytic cycle. In the following, we reacted acene bromides **2 g** and **2 h** with *H*−DTP **1** yielding 2‐naphthyl−DTP **3 g** and 2‐anthryl−DTP **3 h**. Here, Pd_2_dba_3_/*t*Bu_3_P gave slightly better results compared to the other catalysts. We also tested functionalized and electron‐deficient acene bromides, among them 4‐bromo‐1,8‐naphthalic anhydride **2 i** (N−Anh), N‐hexyl‐4‐bromo‐1,8‐naphthalimide **2 j** (N−Imi), and extended 9‐bromoperylene‐3,4‐dicarboximide (PDCI) **2 k**. Whereas anhydride **2 i** reacted very slowly and gave only low yields of DTP **3 i** with the Pd‐catalysts, no reaction with CuI was observed. Dicarboximides **2 j** and **2 k** were much more reactive and were converted to corresponding covalently linked push‐pull donor (D)‐acceptor (A) DTPs **3 j** and **3 k** in good to excellent yields after purification. These promising results prompted us to further investigate in particular *PDCI*−DTP **3 k** as building block for the synthesis of advanced photoactive materials and their application in single material organic solar cells (see below).

The use of various catalytic systems and various aryl and acene bromides showed that Pd_2_dba_3_/*t*Bu_3_P had the broadest applicability with continuous good to excellent yields. The other Pd‐catalyst Pd(OAc)_2_/*t*Bu_3_P is as well broadly applicable, but by trend give somewhat lower yields. The only exception in the series was the completely failed reaction of sterically hindered 2‐bromo‐1,1′‐biphenyl **2 f** with *H*−DTP **1**, for which CuI worked to some degree. In general, CuI under microwave‐assistance gave more or less equal results. The only reaction, which failed was with anhydride **2 i**. The structures of the novel aryl and acene‐substituted DTPs **3 a**–**3 k** were fully characterized by NMR‐spectroscopy (Figures S1–S9, Supporting Information) and high‐resolution mass spectra (HRMS) (Figures S15–S20, Supporting Information). Thus, we can conclude that with respect to electronic effects and tolerance of functional groups the presented arylation method of *H*−DTP **1** with aryl bromides is quite broadly applicable leading to interesting functionalized DTP derivatives. The more challenging examples, bromides **2 f** and **2 i**, could be improved further by optimizing the applied catalyst system.^[18]^

### Quantum chemical DFT‐calculations on DTPs

**Figure 1:**
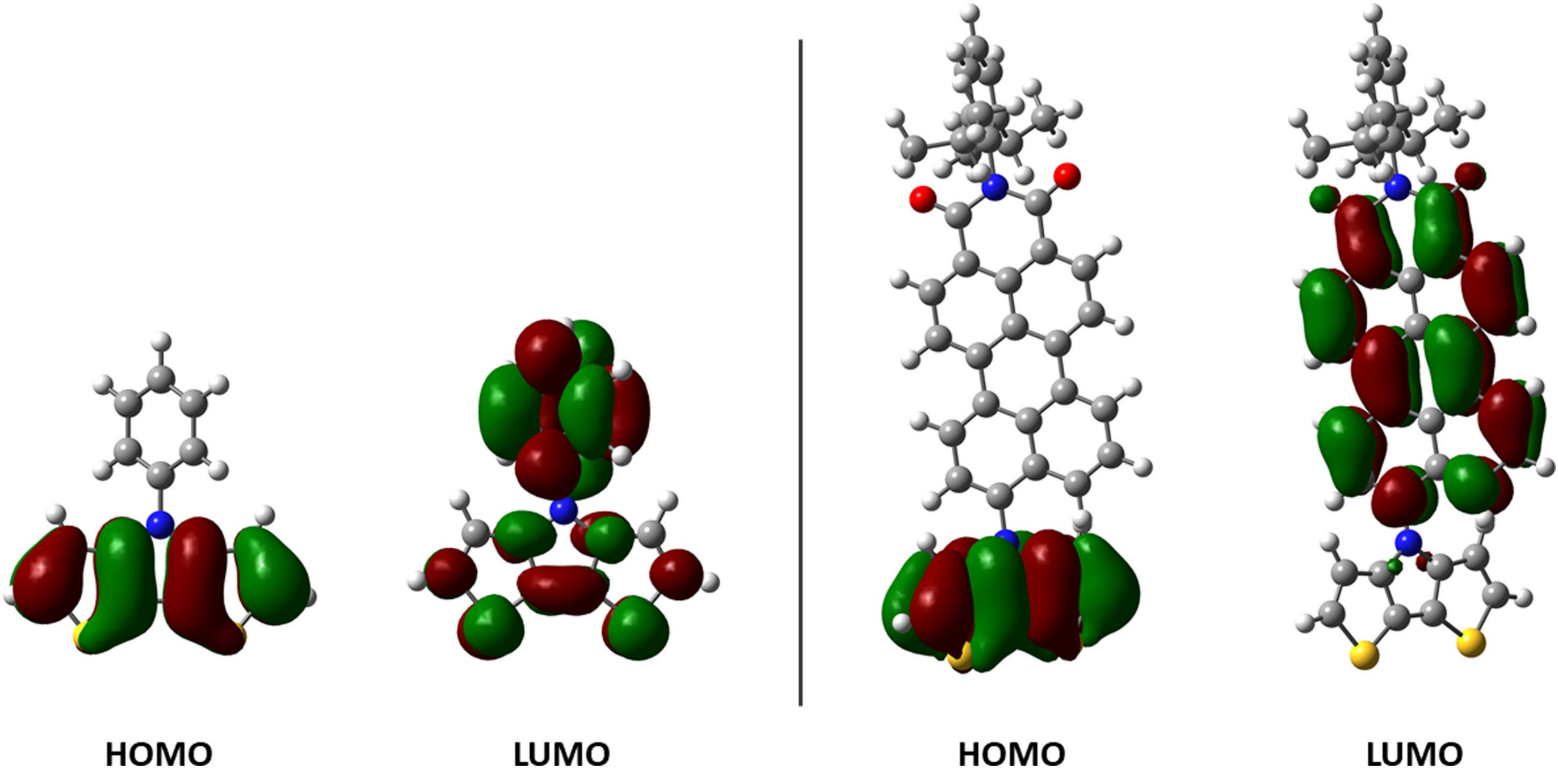
Electron density representation of the frontier molecular orbitals of *Ph*−DTP **3 a** (left) and *PDCI*−DTP **3 k** (right). Hydrogen atoms have been omitted for clarity.

DFT calculations were performed on parent *Ph*−DTP **3 a** in comparison to *PDCI*−DTP **3 k** in order to rationalize the electronic and steric influence of the substituents on the distribution of electron density in the frontier molecular orbitals. Thus, HOMO and LUMO of *Ph*−DTP **3 a** and donor−acceptor *PDCI*−DTP **3 k** are depicted in Figure 1. Previously, it was shown that in alkyl‐substituted DTPs the HOMO is only located at the DTP backbone with the nitrogen residing at a node.^[19]^ Herein, the same observation was made for the DTPs under investigation and substituents only slightly influence the HOMO energy level by inductive effects. In contrast, the LUMO in *Ph*−DTP **3 a** extends over the whole molecule with the expected quinoidal reorganization of the electron density in the DTP unit. The electron‐withdrawing perylene dicarboximide residue in *PDCI*−DTP **3 k** strongly influences the LUMO, which is fully localized on the π‐conjugated part of the PDCI moiety (Figure 1). Whereas the calculated torsion angle of the phenyl unit versus the DTP plane amounts to 37°, the respective calculated angle with the more sterically demanding PDCI‐substituent is 51°.

### Optical and redox properties of arylated DTPs 3 a–3 k

**Figure 2:**
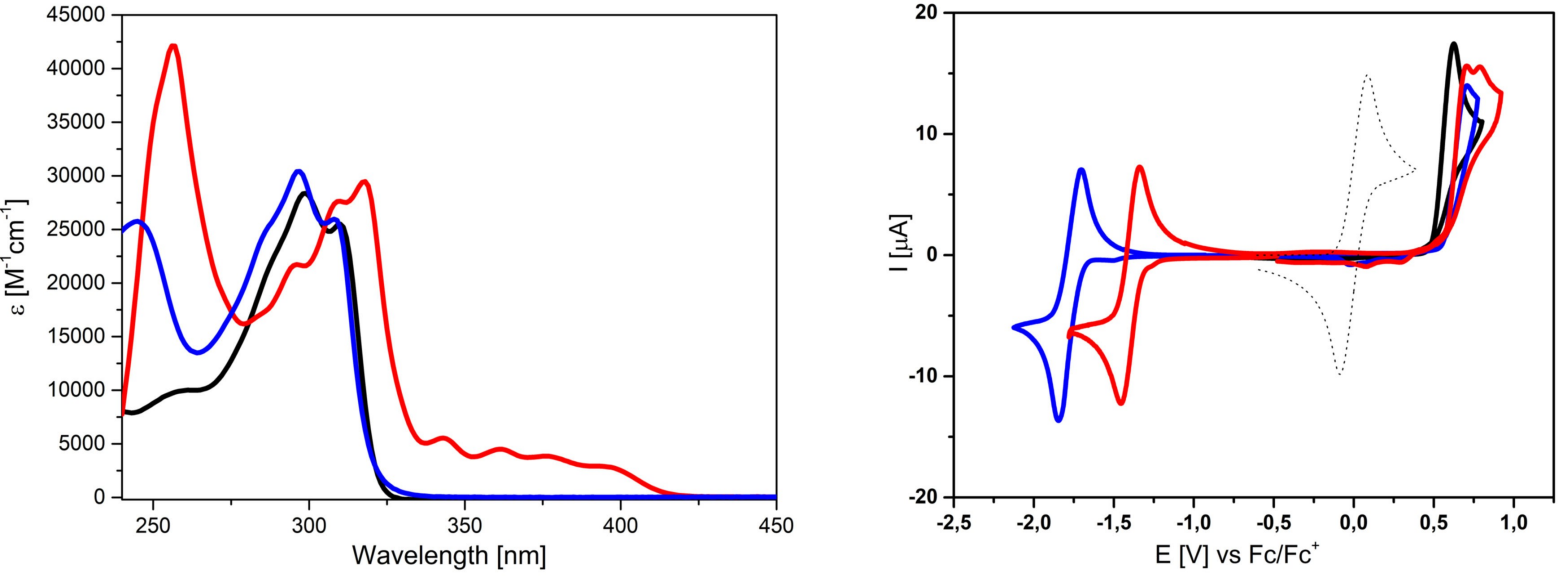
UV‐vis absorption spectra of *Ph*−DTP **3 a** (black curve), *2‐Bph*−DTP **3 f** (blue), and *2‐Anth*−DTP **3 h** (red) in THF solution (left) and cyclic voltammograms of *Ph*−DTP **3 a** (black curve) in comparison to acceptor‐substituted DTP **3 j** (red) and **3 k** (blue) in dichloromethane/tetrabutylammonium hexafluorophosphate (0.1 M), 100 mV/s, cyclic voltammogram of ferrocene (black, dotted) as reference (right).

Optical investigations on the various arylated DTPs were performed by UV‐vis absorption spectra in THF solution. Representative absorption spectra of DTPs **3 a**, **3 f**, and **3 h** are depicted in Figure 2 (left) and full data is listed in Table 2. In general, all DTPs exhibited a strong, mostly structured absorption band in the UV‐regime between 275–325 nm, which we address to the π‐π* transition of the DTP unit and corresponds to the HOMO‐LUMO energy gap. The absorption maximum for this transition is gradually red‐shifted from 294 nm for parent *H*−DTP **1** to 320 nm for fluorenyl−DTP **3 e** due to increasing conjugation of the substituent at the nitrogen. Substituents with extended π‐conjugation superimpose their specific absorption signature, which is most prominent for 2‐anthryl derivative **3 h** (Figure 2, left). The strong absorption band at 256 nm and the weaker absorption at 343 nm, 361 nm, 376 nm, and 394 nm can be addressed to the anthracene unit.^[20]^ Sterical hindrance works in the opposite direction which can be seen for biphenyls **3 d** and **3 f**. Whereas linear 4′‐substituted biphenyl in **3 d** exerts additional conjugation to the system and absorbs at 311 nm, the sterically hindered and twisted 2′‐biphenyl in **3 f** blue‐shifts the main absorption band to 297 nm and an additional band at 245 nm arises due to the biphenyl unit (Figure 2, left).^[20]^ On the other hand, electron‐withdrawing units in DTPs **3 i**, **3 j**, and **3 k** cause substantial red‐shifts of the longest wavelength band up to 513 nm for **3 k** due to partial charge‐transfer character in the transition (Figure 4, left). A smaller red‐shift of around 20 nm can be seen for the main absorption of cyanophenyl−DTP **3 c** compared to *Ph*−DTP **3 a**, whereas the electron‐donating anisyl‐substituent in *MeOPh*−DTP **3 b** practically does not influence absorption. This effect can be rationalized with the findings from electrochemistry and quantum chemical calculations (see below). Accordingly, the optical energy gaps *E*_g_^opt^ of the acceptor‐substituted DTPs are substantially lower compared to the arylated derivatives.

**Table 2:** Optical data of arylated DTPs **3 a**–**3 k**.

DTP	R	$\lambda_{max}^{sol}$^[a]^ [nm]	*ϵ* [M^−1^ cm^−1^]	λ_onset_ [nm]	*E*_g_^opt^ [eV]^[b]^
**3 a**	Ph	298, 310	28 330	321	3.86
**3 b**	MeOPh	297, 309	28 300	323	3.84
**3 c**	NCPh	293, 318	33 800	340	3.65
**3 d**	4′‐Bph	286, 311	38 700	335	3.70
**3 e**	Flu	293, 320	38 400	343	3.62
**3 f**	2′‐Bph	245, 297, 308	30 410	320	3.88
**3 g**	2‐Naph	262, (290), 308	24 600	352	3.52
**3 h**	2‐Anth	257, (296, 309), 318, (343, 361, 376, 394)	42 120	415	2.99
**3 i**	N‐Anh	295, 306, 399	24 370	454	2.73
**3 j**	N‐Imi	295, 306, 394	28 350	445	2.79
**3 k**	PDCI	266, 296, 513	36 810	560	2.21

[a] Absorption spectra measured in THF, maxima underlined, shoulders in brackets. [b] Onset absorption determined by tangent line and *E*_g_^opt^ calculated by *E*=1240/λ_onset_ (nm).

**Figure 3:**
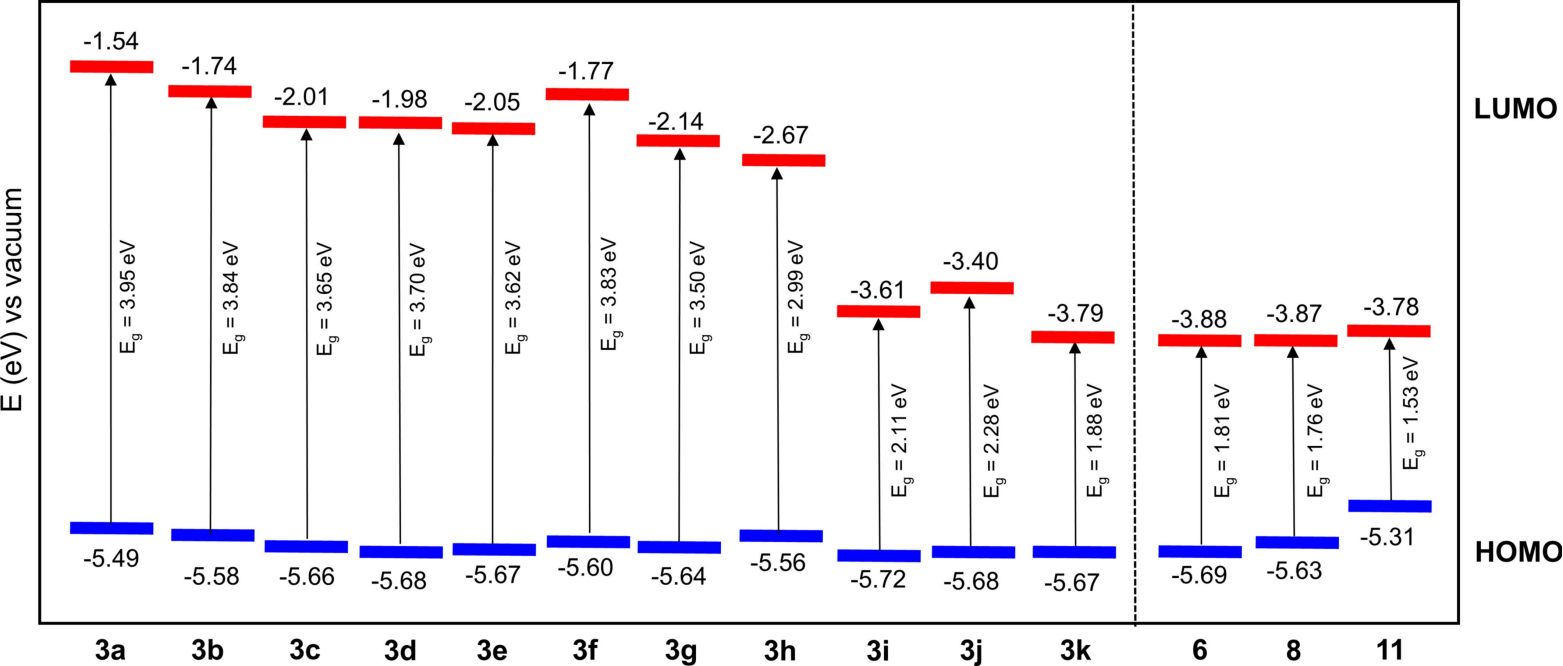
Energy diagram showing the HOMO (blue) and LUMO energy levels (red) and energy gaps *E*_g_ of arylated DTPs **3 a**–**3 k** and extended donor−acceptor systems **6**, **8**, and **11**.

In order to get information about the redox properties and energetics of the frontier orbitals, arylated DTPs **3 a**–**3 k** were studied by cyclic voltammetry in dichloromethane and tetrabutylammonium hexafluorophosphate (0.1 M) as electrolyte and all potentials were referenced against the ferrocene/ferricenium couple (Fc/Fc^+^). Representative examples are depicted in Figure 2 (right) and data is compiled in Table 3. All derivatives gave irreversible oxidation waves due to the reactive α‐positions of the DTP unit and the follow‐up polymerization of the radical cations to form conducting DTP−polymers.^[5]^ The peak potentials vary in a relatively small window from 0.49 V for *Ph*−DTP **3 a** to 0.75 V for naphthalic anhydride **3 i**. In the reductive potential regime, acceptor‐substituted DTPs **3 i**, **3 j**, and **3 k** showed reversible reduction waves at −1.57 V, −1.78 V, and −1.40 V due to the formation of stable radical anions according to the acceptor strength (Figure 2, right). In this respect, the measurements showed that the anhydride residue in DTP **3 i** represents the stronger electron‐withdrawing group compared to the corresponding alkylated dicarboximide in DTP **3 j**.

**Table 3:** Electrochemical data of arylated DTPs **3 a**–**3 k**.

DTP	R	*E*_p_^Ox1^ [V]	*E*_onset_^Ox^ [V] ^[a]^	*E*_1/2_^Red1^ [V]	*E*_onset_^Red^ [V] ^[b]^	HOMO [eV]^[c]^	LUMO [eV]^[d]^	*E*_g_ [eV]^[e]^
**3 a**	Ph	0.49	0.39	–	–	−5.49	−1.54	3.95
**3 b**	MeOPh	0.56	0.48	–	–	−5.58	−1.74	3.84
**3 c**	NCPh	0.65	0.56	–	–	−5.66	−2.01	3.65
**3 d**	4′‐Bph	0.74	0.58	–	–	−5.68	−1.98	3.70
**3 e**	Flu	0.71	0.57	–	–	−5.67	−2.05	3.62
**3 f**	2′‐Bph	0.60	0.50	–	–	−5.60	−1.77	3.83
**3 g**	2‐Naph	0.64	0.54	–	–	−5.64	−2.14	3.50
**3 h**	2‐Anth	0.64	0.56	–	–	−5.56	−2.67	2.99
**3 i**	N‐Anh	0.75	0.62	−1.57	−1.49	−5.72	−3.61	2.11
**3 j**	N‐Imi	0.70	0.58	−1.78	−1.70	−5.68	−3.40	2.28
**3 k**	PDCI	0.71	0.56	−1.40	−1.31	−5.67	−3.79	1.88

[a] Onset voltage determined by applying a tangent line. [b] Calculated from the onset value of the oxidation wave; Fc/Fc^+^ was set to −5.1 eV vs. vacuum. [c] Calculated from the onset value of the reduction wave or with E_HOMO_ and *E*_g_^opt^ sol. [d] Calculated E_HOMO_‐E_LUMO_.

HOMO energy levels were determined from the onset of the oxidation wave and are only marginally influenced by the substituents and range from −5.49 eV to −5.72 eV due to a node at the DTP−nitrogen in this orbital. On the contrary, the LUMO energies are substantially influenced by the electron‐withdrawing power of the aryl and acene groups. With increasing acceptor strength, the LUMO is increasingly destabilized and energies decrease from −1.54 eV for *Ph*‐−DTP **3 a** to −3.79 eV for PDCI−DTP **3 k** concomitant with a decrease of the electrochemical energy gap *E*_g_^CV^ from 3.95 eV to 1.88 eV. A resulting schematic energy level diagram of the frontier orbitals and the optical transitions in the series of investigated DTPs **3 a**–**3 k** was derived and is shown in Figure 3.

### Synthesis of ambipolar donor−acceptor dyads for organic electronic application

**Scheme 2:**
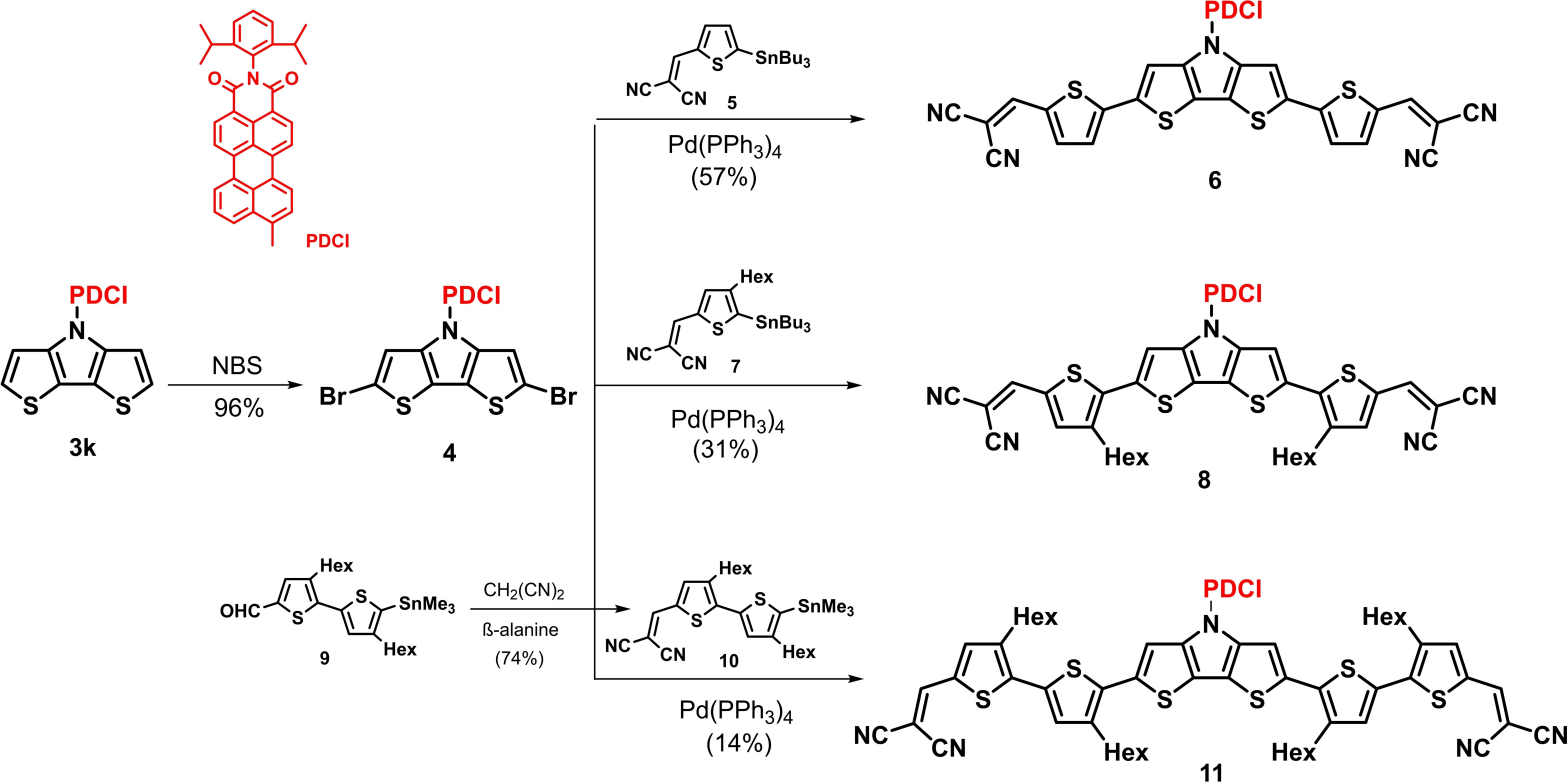
Synthesis of extended D−A dyads **6**, **8**, and **11** by Stille‐type coupling of dibrominated *PDCI*−DTP **4** with stannyls **5**, **7**, and **10**.

As already mentioned, DTP‐units have been frequently used as building block in conjugated materials for application in organic electronics, in particular for organic solar cells.[^1^, ^2^, ^3^, ^4^] In this respect, over the years we have developed DTP‐based co‐oligomers, which in conjunction with fullerenic acceptors (PCBM) reached power conversion efficiencies (PCE) of 7–8 % in solution‐processed bulk‐heterojunction organic solar cells (BHJ‐OSC).^[21]^ In order to further develop this structural concept, we implemented *PDCI*−DTP **3 k** as a core unit into enlarged oligomers, which are terminated with dicyanovinylene (DCV) acceptor groups and should strongly absorb in the visible regime of the solar spectrum. For the synthesis of enlarged D−A oligomers, *PDCI*−DTP **3 k** was brominated at the α‐positions with NBS to give dibromo−DTP **4** in 96 % yield, which was subsequently coupled with various stannylated dicyanovinylene (DCV)‐substituted thiophene and bithiophene units (Scheme 2). In this respect, DTP **4** was reacted with stannylated thiophene **5** ^[22]^ under Pd‐catalysis in a Stille‐type coupling reaction to give D−A dyad **6** in 57 % yield. Due to the resulting moderate solubility hexyl side chains were implemented at the outer thiophene units and DTP **4** was reacted with the corresponding 3‐hexyl derivative **7** ^[23]^ to give analogous D−A dyad **8** in 31 % yield. Further enlargement of the donor moiety with additional 3‐hexylthiophene units was obtained by reaction of brominated *PDCI*−DTP **4** and stannylated DCV−bithiophene **10**, which was synthesized in 74 % yield from corresponding aldehyde **9** and malononitrile. Cruciform‐type D−A dyad **11** was isolated as a black solid in moderate 14 % yield after purification. Structures and purities of D−A dyads **6**, **8**, and **11** were fully characterized by NMR‐spectroscopy (Figures S10–S14, Supporting Information) and HRMS (Figures S21–S25, Supporting Information).

**Figure 4:**
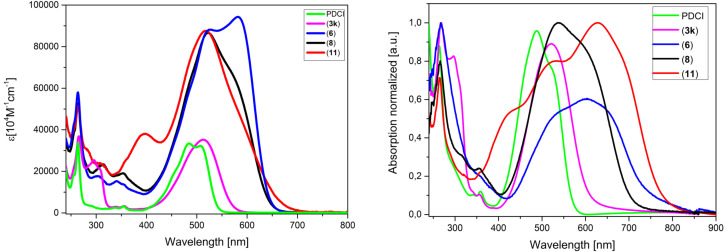
UV‐vis absorption spectra of D−A dyads **6** (blue), **8** (black), and **11** (red) in comparison to DTP **3 k** (magenta) and perylene‐3,4‐dicarboximide (PDCI, green) in dichloromethane solution (left); in thin films spin‐coated from chloroform solution and normalized to the highest energy absorption (right).

The optical properties of D−A dyads **6**, **8**, and **11** were determined by UV‐vis absorption in dichloromethane solution and spectra are shown in comparison to those of basic DTP **3 k** and substituent PDCI in Figure 4 (left), data is collected in Table 4. The absorption spectra reflect more or less the specific absorptions of the subunits, which are superimposed. The spectra are dominated by the strong absorptions of the PDCI acceptor unit at around 265 nm and 500–530 nm. The absorptions of the donor units rather appear as shoulders, which is at 295 nm for the DTP unit of **3 k** in accordance with the UV‐vis spectra of the other arylated DTPs.

**Table 4:** Optical properties of D−A dyads **6**, **8**, and **11** in comparison to DTP **3 k** and perylene‐3,4‐dicarboximide (PDCI) in dichloromethane solution and in thin films.

Oligomer	$\lambda_{max}^{sol}$^[a]^ [nm]	lg ϵ	$E_{g}^{sol}$^[b]^ [eV]	$\lambda_{max}^{film}\;$^[c]^ [nm]	$E_{g}^{film}\;$^[b]^ [eV]
**3k**	266, 295, 513	4.57	2.14	268, 297 (sh), 521	2.05
**6**	264, 304, 527, 582	4.97	1.93	268, 533 (sh), 603	1.65
**8**	264, 314, 523, 574 (sh)	4.94	1.90	265, 538	1.77
**11**	264, 397, 517, 570 (sh)	4.94	1.86	265, 430 (sh), 529 (sh), 629	1.61
**PDCI**	265, 486, 509	4.52	2.25	265, 488	2.17

[a] Absolute maximum is underlined, (sh) shoulder. [b] Energy gaps *E*_g_ determined by applying a tangent line and the formula *E*_g_=1240/λ_onset_ (nm). [c] Films were spin‐coated on glass slides from chloroform solution.

With increasing conjugation length of the donor block in **6**, **8**, and **11**, these π‐π* transitions, which comprise partial charge‐transfer (CT) character due to the terminal DCV‐substitution, appear as shoulders at lower energies of the main PDCI band and are red‐shifted to 570–582 nm. These values well correlate with the main absorption bands of the corresponding DCV‐substituted tetrameric^[23]^ and hexameric^[24]^ co‐oligomers without PDCI group at the DTP unit (560–612 nm). For the longest oligomer **11** an additional band with a maximum at 397 nm is visible which corresponds to the π‐π* transition of the pure oligothiophene subunit without attached DCV groups.^[25]^ The optical gaps, which were determined from the onset of the longest wavelength absorption, gradually decrease from 2.25 eV for pure PDCI to 1.86 eV for the most extended oligomer **11**.

Thin films, which were prepared by spin‐coating on glass slides from chloroform solutions (Figure 4, right; Table 3), showed the typical broadening and bathochromic shift of the absorption bands compared to the solution spectra. This behavior is most pronounced for D−A dyad **11**, where now the π‐π* transition of the donor part is shifted to 629 nm and promoted to the most intensive band. We address this behavior to partial ordering and π‐π stacking of the oligothiophene backbone. Compared to the values determined from the solution spectra, the optical gaps are concomitantly decreased for each derivative and range from 2.17 eV for PDCI to 1.61 eV for **11**.

**Figure 5:**
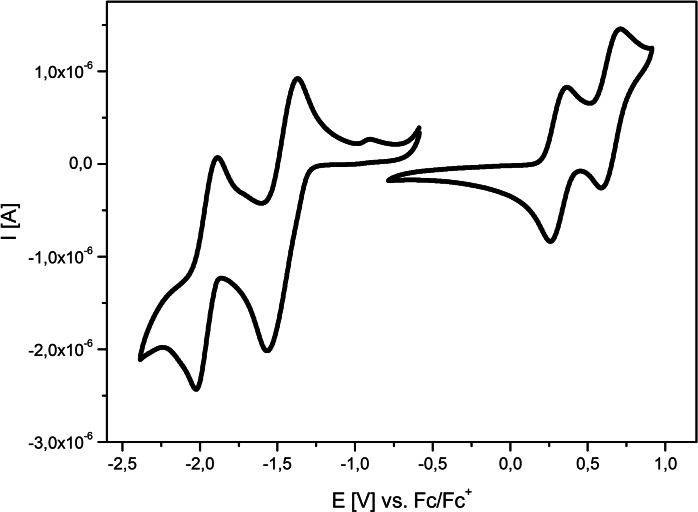
Cyclic voltammogram of D−A dyad **11** in dichloromethane/tetrabutylammonium hexafluorophosphate (0.1 M) at 100 mV/s. Potentials vs. ferrocene/ferricenium (Fc/Fc^+^).

The redox properties of D−A oligomers **6**, **8**, and **11** were studied by cyclic voltammetry (CV) and differential pulse voltammetry (DPV) in dichloromethane and tetrabutylammonium hexafluorophosphate (0.1 M) as electrolyte, potentials were referenced against the ferrocene/ferricenium couple (Fc/Fc^+^) and data is compiled in Table 5. Exemplarily, the CV of oligomer **11** is shown in Figure 5, while those of **6** and **8** are depicted in Figure S26 (Supporting Information). Four reversible redox waves can be identified, two in the oxidative, two in the reductive potential range. We could address the various redox waves by comparing the CVs of individual subunits. The first one‐electron oxidation occurs at *E*_1/2_^Ox1^=0.32 V, whereas the second oxidation occurs at *E*_1/2_^Ox2^=0.65 V. The latter corresponds to the formation of stable radical cations and dications, respectively, which are located on the extended oligomeric DTP−donor part.^[25]^ The first reduction wave at *E*_1/2_^Red1^=−1.46 V reflects a superimposition of the reversible one‐electron reduction of the PCDI‐substituent^[25]^ and the irreversible reduction of the terminal DCV groups.^[25]^ The second reversible one‐electron reduction at *E*_1/2_^Red1^=−1.96 V is addressed to the further reduction of the PDCI unit under the formation of stable dianions.

**Table 5:** Electrochemical properties of D−A dyads **6**, **8**, and **11** in comparison to DTP **3 k** and perylene‐3,4‐dicarboximide (PDCI) in dichloromethane/tetrabutylammonium hexafluorophosphate (0.1 M) at 100 mV/s. Potentials vs. ferrocene/ferricenium (Fc/Fc^+^).

Oligomer	*E*_1/2_^Ox1^ [V]	*E*_1/2_^Ox2^ [V]	*E*_on_^Ox1^ [V] ^[a]^	*E*_1/2_^Red1^ [V]	*E*_1/2_^Red2^ [V]	*E*_on_^Red1^ [V] ^[a]^	HOMO [eV]^[b]^	LUMO [eV]^[c]^	*E*_g_^CV^ [eV]^[d]^
**3 k**	0.71	–	0.56	−1.40	–	−1.31	−5.66	−3.79	1.87
**6**	0.66	1.13^[e]^	0.59	−1.34	−1.83	−1.22	−5.69	−3.88	1.81
**8**	0.59	1.06	0.53	−1.35	−1.83	−1.23	−5.63	−3.87	1.76
**11**	0.32	0.65	0.21	−1.46	−1.96	−1.32	−5.31	−3.78	1.53
**PDCI** ^[f]^	0.95	–	0.87	−1.46	−1.95	−1.33	−5.97	−3.77	2.20

[a] Onset voltage determined by applying a tangent line. [b] Calculated from the onset value of the oxidation wave; Fc/Fc^+^ was set to −5.1 eV vs. vacuum. [c] Calculated from the onset value of the reduction wave. [d] Calculated by *E*_HOMO_‐*E*_LUMO_. [e] Determined by DPV. [f] Values from Ref. [25].

HOMO and LUMO energy levels of −5.31 eV and −3.78 eV were determined from the onset potential of the first oxidation and reduction wave, respectively. The same behavior is noticed for the other D−A dyads, whereby the reduction potentials are very similar showing that there is little π‐conjugation between the reduced PDCI subunit and the DTP‐based donor due to the cruciform‐type connection. The oxidation potentials expectedly depend on the conjugated length of the donor subunit and gradually decrease from 0.71 V for DTP **3 k** to 0.32 V for the longest oligomer **11**. As a consequence, the electrochemical gap is step‐by‐step reduced from 1.87 eV for **3 k** to 1.53 eV for **11**.

### Photovoltaic properties of cruciform‐type D−A oligomers 8 and 11 in single material organic solar cells

Covalently linked, ambipolar molecular donor (D)‐acceptor (A) dyads have been used as sole photoactive component in single material organic solar cells (SMOSC).^[26]^ We could recently demonstrate power conversion efficiencies (PCE) of around 4 % by linking the same DTP‐based oligothiophene donor as in novel dyad **11** and fullerene PC_61_BM with alkyl ester spacers of variable length.^[27]^ However, directly connected and π‐conjugated D−A systems have been rarely described and reached lower PCEs of 0.35 % to 1.75 %.[^28^, ^29^, ^30^] A cruciform‐type D−A molecule comprising a benzodithiophene core and a calculated distortion of 56° between the D and A‐axes in the molecule showed ambipolar semiconducting behavior in organic field‐effect transistors.^[31]^ In this respect, our DTP oligomer **3 k**, in which the PDCI acceptor is tilted by 51° with respect to the DTP donor (see above) represents an interesting alternative of a cruciform‐type core system. We assume that in the related extended structures of dyads **6**, **8**, and **11** the PDCI unit is similarly tilted. Due to their broad and strong absorption in the visible and appropriate HOMO/LUMO energy levels, these dyads should be appropriate candidates for SMOSCs.

Because oligomer **6** was only barely soluble in organic solvents, we implemented the better soluble derivatives **8** and **11** as sole photoactive component in SMOSCs with the standard device structure glass/ITO/PEDOT:PSS/dyad **8** or **11**/LiF/Al. SMOSCs were built by spin‐coating solutions with concentrations of 10–15 mg mL^−1^ under ambient conditions. Thereby, various solvents, additives, and post‐treatment methods, such as solvent vapor annealing (SVA) or thermal annealing (TA) were tested. The best performing solar cells unfortunately showed very low performances and we could not exceed PCEs of 0.05 % for both dyads (Table 6, *J*‐*V* curves in Figure S27, Supporting Information). We assume that despite beneficial optoelectronic properties, the crossed subunits of the dyads hamper a favorable arrangement of the molecules in the photoactive film for the formation of suitable channels for the separate charge transport of holes and electrons which explains the rather moderate fill factor (FF) and low photocurrent densities *J*_SC_. Therefore, thermal deactivation of the excitons formed under illumination and recombination of charges seem to be dominating processes. A further drawback was the moderate solubility of dyad **8** and **11** not allowing for the preparation of optimal films. From these rather disappointing results in SMOSCs, we can conclude that for good performance in SMOSCs, the connection of donor and acceptor units by flexible insulating linkers^[27]^ seems to be more favorable than a direct cruciform‐type connection.

**Table 6:** Photovoltaic parameters of SMOSCs with glass/ITO/PEDOT:PSS/dyad **8** or **11**/LiF/Al.^[a]^

	Solvent	SVA/TA	*V*_OC_ [V]	*J*_SC_ [mA/cm^2^]	FF	PCE [%]
Dyad **8**	CF, r.t.	CF 20 s	0.98±0.02 (1.02)	0.21±0.00 (0.21)	0.24±0.00 (0.24)	0.05±0.00 (0.05)
Dyad **11**	CB, 80 °C	100 °C, 10 min	0.72±0.04 (0.77)	0.24±0.01 (0.23)	0.23±0.00 (0.23)	0.04±0.00 (0.04)

[a] Average values for 4 devices ± standard deviation (best value). CF=chloroform; CB=chlorobenzene.

## Conclusion

In summary, we have presented a novel, direct and versatile N‐arylation method of dithienopyrrole *H*−DTP **1** with widely available aryl or acene bromides **2 a**–**k** by efficient Pd‐ or Cu‐catalyzed coupling. The direct introduction of various aryls and acenes with different electronic and steric properties at the DTP nitrogen yielded intriguing functionalized DTPs **3 a**–**k**. The use of three catalytic systems, Pd(OAc)_2_/*t*Bu_3_P, Pd_2_dba_3_/*t*Bu_3_P, or CuI under microwave‐assistance, showed different selectivities and provided a broad scope of products with mostly good to excellent yields. Investigations on optical and redox properties of the functionalized DTPs led to valuable structure‐property relationships. Absorption spectra and cyclic voltammograms were typically a superimposition of the DTP properties and the specific signatures of the substituent. The results were corroborated by quantum chemical calculations revealing that the substituents at the DTP nitrogen have only little influence on the HOMO, whereby the distribution of electron density in the LUMO is rather delocalized on the substituent, in particular for the electron acceptors. Perylene dicarboximide‐substituted DTP **3 k** was further elaborated to result in conjugated cruciform‐type donor−acceptor oligomers, which were characterized and tested in single material organic solar cells.

## Conflict of interest

The authors declare no conflict of interest.

## Supporting information

As a service to our authors and readers, this journal provides supporting information supplied by the authors. Such materials are peer reviewed and may be re‐organized for online delivery, but are not copy‐edited or typeset. Technical support issues arising from supporting information (other than missing files) should be addressed to the authors.

Supporting Information
