# Supplementary material for: Broadly Applicable Synthesis of Arylated Dithieno[3,2‐b:2′,3′‐d]pyrroles as Building Blocks for Organic Electronic Materials
Source: Chemistry. 2021 Jul 29;27(48):12362–70. doi: 10.1002/chem.202101478 (PMC8456814; doi:10.1002/chem.202101478)
Supplement: Supplementary file 1 — Supporting Information [file CHEM-27-12362-s001.pdf]

Chemistry–A European Journal

Supporting Information

Broadly Applicable Synthesis of Arylated Dithieno[3,2-*b*:2′,3′-*d*]pyrroles as Building Blocks for Organic Electronic Materials

Astrid Vogt, Fabian Schwer, Sebastian Förtsch, Christoph Lorenz, Elena Mena-Osteritz, Anna Aubele, Teresa Kraus, and Peter Bäuerle*

Supporting Information

for **Broadly Applicable Synthesis of Arylated Dithieno[3,2-*b*:2',3'-*d*]pyrroles as Building Blocks for Organic Electronic Materials**

Table of Content

Experimental Section	page
1. Instruments and measurements	S2
2. Materials	S4
3. Synthesis	S5
4. ^{1}H-, ^{13}C-NMR spectra	S13
5. High resolution mass spectra	S27
6. Cyclovoltammograms	S33
7. Photovoltaic properties	S33
8. Notes and references	S34

Experimental Section

1. Instruments and measurements. NMR spectra were recorded on an Avance 400 (^{1}H NMR: 400 MHz, ^{13}C NMR: 101 MHz) or a Bruker AMX 500 spectrometer (^{1}H NMR: 500 MHz, ^{13}C NMR: 125 MHz). Chemical shifts (δ) are reported in ppm using residual solvent protons (^{1}H NMR: δ_H = 7.26 for $CDCl_3$; δ_H = 5.91 for $C_2D_2Cl_4$; ^{13}C NMR: δ_C = 77.16 for $CDCl_3$; δ_C = 73.78 for $C_2D_2Cl_4$) as internal standard. The splitting patterns are designated as follows: s (singlet), d (doublet), t (triplet), and m (multiplet). Coupling constants *J* relate to proton-proton couplings. Protons at the α- or β-positions of DTPs were assigned as Th-H_α or Th-H_β, respectively. Thin layer chromatography was carried out on aluminum plates, precoated with silica gel, Merck Si60 F254. Preparative column chromatography was performed on glass columns packed with silica gel (particle size 40–63 μm) from Macherey-Nagel or aluminium oxide, Merck 90 active basic, particle size 63-200 μm. HPLC was performed on a Shimadzu CBM-20A equipped with a SPD-20A UV-Vis detector and a LC-8A solvent delivery system using a Macherey-Nagel column (Nucleosil 100-5 NO_2). Melting points were determined using a Büchi Melting Point B-545 (not corrected) or a Mettler Toledo DSC 823e under argon flow (heating rate 10 °C/min). Thermogravimetric analyses were carried out with a TGA/SDTA 851e from Mettler Toledo. GC-MS measurements were performed on a Shimadzu GCMS-QP2010 SE. Elemental analyses were performed on an Elementar Vario EL. GC/EI-MS (70 eV) measurements were performed on a Shimadzu GCMS-QP2010 SE. High resolution MALDI

mass spectra were performed on a Bruker SolariX using trans-2-[3-(4-*tert*-butylphenyl)-2-methyl-2-propenylidene]malononitrile (DCTB) as matrix. Chemical ionisation (CI) mass spectra were recorded on a Finnigan MAT, SSQ-7000. EI mass spectra were recorded on a Varian Saturn 2000 GC-MS. High resolution APCI spectra were performed on a Bruker SolariX using acetonitrile as solvent. For the microwave-assisted synthesis vials of 10 ml were used in a microwave reactor (CEM Discover).

Optical measurements in solution were carried out in 1 cm cuvettes with Merck Uvasol grade solvents. Absorption spectra were recorded on a Perkin Elmer Lambda 19 spectrometer. Cyclic voltammetry experiments were performed with a computer-controlled Autolab PGSTAT30 potentiostat in a three-electrode single-compartment cell (3 mL). The platinum working electrode consisted of a platinum wire sealed in a soft glass tube with a surface of A = 0.785 mm^2, which was polished down to 0.25 µm with Buehler polishing paste prior to use to guarantee reproducible surfaces. The counter electrode consisted of a platinum wire and the reference electrode was an Ag/AgCl reference electrode. All potentials were internally referenced to the ferrocene/ferricenium couple (Fc/Fc^+). For the measurements, concentrations of 10^{-3} M of the electroactive species were used in freshly distilled and deaerated dichloromethane (Lichrosolv, Merck) purified with a Braun MB-SPS-800 and 0.1 M $(n\text{-}Bu)_4NPF_6$ (Fluka; recrystallized twice from ethanol).

Device fabrication of organic solar cells was performed with indium tin oxide (ITO) patterned glass substrates (15 Ω cm^{-2}, from Naranjo Substrates), which were precleaned with acetone, Mucasol soap solution, and isopropanol, before treated in a UV-ozone cleaner for 30 min. PEDOT:PSS (Ossila) was spin-coated at 3000 rpm to obtain thin films of 20-30 nm thickness. The active layer solutions (10 mg/mL to 20 mg/mL in chloroform, chlorobenzene or tetrachloroethane) were deposited by spin-coating at various spin speeds. Thin films of lithium fluoride (LiF, 0.7 nm) and aluminum (Al, 100-120 nm) were deposited by high-vacuum evaporation at pressures below 3 x 10^{-6} mbar (Nano 36, from Kurt J. Lesker Co.). Solvent vapor annealing (SVA) was performed by a literature known procedure.[1] One substrate contained four photoactive areas of 0.09 and 0.16 cm^2. *J*-*V*-characteristics of the prepared devices were obtained by using a solar simulator (Oriel Instruments, class AAA, AM 1.5G, 100 $mWcm^{-2}$). Apart from LiF and aluminum deposition, processing and characterization of the solar cells were performed under ambient conditions. The external quantum efficiency (EQE) was measured under monochromatic light from a 300 W Xenon lamp in combination with a mono-

chromator (Oriel, Cornerstone 260), modulated with a mechanical chopper. The response was recorded as the voltage over 220 Ω resistance using a lock-in amplifier (Merlin 70104). A calibrated Si-cell was used as a reference (Newport 70356-70316NS).

Quantum chemical calculations were carried out with the Gaussian 09 program.[2] Geometry optimizations and energy calculations were performed via DFT methods using the B3LYP[3] or the M06-2X correlation/exchange functional[4] and the 6-311+G(d) or the 6-311G basis set. Molecular orbital surfaces were generated utilizing the Gauss View 5.0 program with an isovalue of 0.02.

2. Materials. Toluene (Sigma Aldrich), THF (Carl Roth GmbH), dichloromethane, DMF (VWR), and diethyl ether (VWR) were dried and purified by a MB SPS-800 (MBraun). Acetone, chloroform, dichloromethane, ethanol, ethyl acetate, methanol, and petroleum ether and were purchased from VWR, chlorobenzene from Merck and distilled prior to use. Cesium carbonate, sodium *tert*-butanolate, $Pd(OAc)_2$, Pd_2dba_3, tri-*tert*-butylphosphine were purchased from Sigma Aldrich, magnesium sulfate from Grüssing GmbH, silica gel from Macherey-Nagel, aluminium oxide, bromine, and copper iodide from Merck. The ligands tBuXPhos and $P(o\text{-}tol)_3$ were purchased from Oxchem. Bromobenzene **2a**, iodobenzene **2a**, phenyl tosylate **2a**, 4-bromoanisole **2b,** and ß-alanine were purchased from Merck. 4-Bromobenzonitrile **2c**, 4-bromo-1,1'-biphenyl **2d**, 2-bromo-9,9-dimethylfluorene **2e**, 2-bromo-1,1'-biphenyl **2f**, 2-bromonaphthalene **2g,** 4-bromo-1,8-naphthalic anhydride **2i**, perylene-3,4,9,10-tetracarboxylic dianhydride, 2,6-diisopropyl aniline, N-bromosuccinimide, and malonodinitrile were purchased from Sigma-Aldrich, 2-bromoanthracene **2h** from Chempur. Poly(3,4-ethylendioxythiophene) polystyrolsulfonate (PEDOT-PSS, Clevios P, VP. AI 4038 aqueous solution) was bought from Heraeus, ultrapure aluminum (99.98%) from Umicore, and lithium fluoride from ACROS.

The following substances were internally synthesized according to literature-known procedures: 4*H*-dithieno[3,2-*b*:2',3'-*d*]pyrrole **1**,[5] 6-bromo-2-hexyl-1H-benzo[*de*]isoquinoline-1,3(2H)-dione **2j**,[6] 9-bromo-*N*-(2,6-diisopropylphenyl)-perylene-3,4-dicarboximide **2k**,[7] 5-[(tributylstannyl)thien-2-yl]methyleneyl)malononitrile **5**,[8] 4-hexyl-5-(tributylstannyl-)thiophene-2-carbaldehyde **7**,[9] 3,4'-dihexyl-5'-(trimethylstannyl)-2,2'-bithiophen-5-carbaldehyde **9**,[9] and $Pd[PPh_3]_4$.[10]

3. Synthesis.

Table S1: Screening of the Buchwald-Hartwig amination of various phenyl halides and tosylate **2a-d** with *H*-DTP **1** to yield *N*-phenyl DTP **3.**[a]

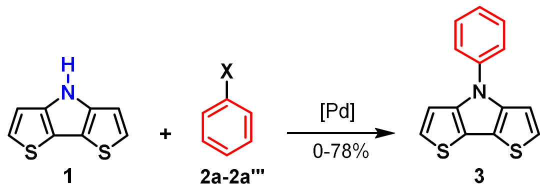

Entry	**2a** (X)	Precatalyst (mmol)	Ligand [mmol]	Solvent	Yield [%]
1	**2a** (Br)	$Pd(OAc)_2$ (0.03)	*t*Bu_3P (0.06)	toluene	**76**
2	**2a** (Br)	$Pd(OAc)_2$ (0.10)	P(*o*-Tol)$_3$ (0.20)	toluene	traces
3	**2a** (Br)	$Pd(OAc)_2$ (0.10)	*t*BuXPhos (0.20)	toluene	48
4	**2a'** (Cl)	$Pd(OAc)_2$ (0.03)	*t*Bu_3P (0.06)	toluene	15
5	**2a''** (I)	$Pd(OAc)_2$ (0.03)	*t*Bu_3P (0.06)	toluene	38
6[f]	**2a'''** (OTos)	$Pd(OAc)_2$ (0.03)	*t*Bu_3P (0.06)	toluene	0
7[b,c]	**2a** (Br)	Pd_2dba_3 (0.05)	*t*Bu_3P (0.10)	*o*-xylene	**78**
8[f]	**2a** (Br)	CuI (0.03)	-	toluene	**76**

[a]Reaction conditions: *H*-DTP **1** (1.0 mmol), phenyl halide/tosylate (1.2 mmol), NaO*t*Bu (2 mmol), solvent (2 mL), 2 days, 110 °C; isolated yields are given; [b]160 °C; [c]0.06 mmol base; [d]140 °C; [e]NEt_3 (0.06 mmol) as additive; [f]3 d.

*General procedure for the reaction of H-DTP **1** and aryl bromides **2a-2k** to arylated DTPs **3a-3k** using the catalytic systems* ***Pd(OAc)₂/tBu₃P*** and ***Pd₂dba₃/tBu₃P***: A suspension of *H*-DTP **1** (100 mg, 0.56 mmol), aryl bromide (0.59 mmol, 1.05 eq), *t*Bu_3P (45 mg, 0.22 mmol, 40 mol%), and $Pd(OAc)_2$ (13 mg, 0.06 mmol, 10 mol%) or Pd_2dba_3 (51 mg, 0.06 mmol, 0.10 eq) were dissolved in toluene (6 mL) was purged with argon. Then, solid NaO*t*Bu (80 mg, 0.84 mmol, 1.50 eq) was added and the reaction mixture heated at 110 °C for 6 d. The crude product was purified via column chromatography (LC) to yield corresponding arylated DTP.

*General procedure for the reaction of H-DTP **1** and aryl bromides **2a-2k** to arylated DTPs **3a-3k** using the catalytic system **CuI** under microwave-assistance (m.w.)*: In a sealed 10 mL microwave vial, *H*-DTP **1** (100 mg, 0.56 mmol, 1 eq), cesium carbonate (215 mg, 0.66 mmol, 1.20 eq), copper iodide (10 mg, 0.06 mmol, 10 mol%), the aryl bromide **2b-2k** (0.66 mmol, 1.20 eq), in dimethylformamide (2 mL) were heated at 180 °C (300 W, 15 bar) for 1 hr. Then,

the reaction was stopped by adding water (15 mL) and three times extracted with ethyl acetate (15 mL). The combined organic phases were washed twice with water (10 mL), dried over magnesium sulfate, filtered, and the solvent removed in vacuo. The crude product was purified via column chromatography on silica gel to yield corresponding arylated DTPs **3b**-**3k**.

4-Phenyl-4*H*-dithieno[3,2-*b*:2',3'-*d*]pyrrole (3a): 4-Bromobenzene **2a** (92 mg, 0.59 mmol); LC: SiO_2, petroleum ether (PE)/dichloromethane (DCM)=3:1; *Ph*-DTP **3b** was isolated as a colourless solid. Yields: *1.* $Pd(OAc)_2$/*t*Bu_3P: 111 mg, 0.43 mmol, 78 %; *2.* Pd_2dba_3/*t*Bu_3P: 108 mg, 0.42 mmol, 76 %; *3.* CuI /m.w.: 127 mg, 0.38 mmol, 76 %. Mp 126-127 °C (lit 126-127 °C); ^{1}H NMR ($CDCl_3$, 400 MHz): δ=7.61 (dd, *J*=7.7, 1.0 Hz, 2H, Ar-H), 7.57-7.51 (m, 2H, Ar-H), 7.38-7.31 (m, 1H, Ar-H), 7.19 (s, 4H, Th-$H_{\alpha,\beta}$), ppm. ^{13}C NMR ($CDCl_3$, 101 MHz): δ=144.1, 140.0, 129.9, 126.1, 123.6, 122.7, 117.1, 112.4, ppm. The analytical data was in accordance with literature.[11,12]

4-(*p*-Methoxyphenyl)-4*H*-dithieno[3,2-*b*:2',3'-*d*]pyrrole (3b): 4-Bromoanisole **2b** (110 mg, 0.59 mmol); LC: SiO_2, PE:DCM=7:3; DTP **3b** was isolated as a colourless solid. Yields: *1.* $Pd(OAc)_2$/*t*Bu_3P: 76 mg, 0.27 mmol, 54 %; *2.* Pd_2dba_3/*t*Bu_3P: 141 mg, 0.49 mmol, 89 %; *3.* CuI/ m.w.: 96 mg, 0.33 mmol, 61 %. The analytical data was in accordance with literature.[12] Mp 133-134 °C; ^{1}H NMR ($CDCl_3$, 400 MHz): δ=7.52-7.47 (m, 2H, Ar-H), 7.16 (d, *J*=5.4 Hz, 2H,Th-H_α), 7.10 (d, *J*=5.3 Hz, 2H, Th-H_β), 7.07-7.02 (m, 2H, Ar-H), 3.88 (s, 3H, C*H*$_3$-H) ppm; ^{13}C NMR ($CDCl_3$, 101 MHz): δ=158.0, 144.5, 133.1, 124.4, 123.4, 116.4, 115.0, 112.1, 55.7 ppm. GC/EI-MS (70 eV): *m/z* (%)=285 (100) $[M]^+$; CI-MS: *m/z* (%)=286 (100) $[M+H]^+$, 178 (2) $[M-C_6H_4OCH_3]^+$; Elemental analysis: calc. (%) for $C_{15}H_{11}NOS_2$: C 63.13, H 3.89, N 4.91, S 22.47; found: C 63.19, H 4.09, N 5.06, S 22.24. The analytical data was in accordance with literature.[11]

4-(*p*-Cyanophenyl)-4*H*-dithieno[3,2-*b*:2',3'-*d*]pyrrole (3c): 4-Bromobenzonitrile **2c** (107 mg, 0.59 mmol); LC: SiO_2, PE:DCM=8:2; DTP **3c** was isolated as a colourless solid. Yields: *1.* $Pd(OAc)_2$/*t*Bu_3P: 83 mg, 0.25 mmol, 59 %; *2.* Pd_2dba_3/*t*Bu_3P: 133 mg, 0.49 mmol, 85 %; *3.* CuI/ m.w.: 81 mg, 0.29 mmol, 52 %. Mp 243-245 °C; ^{1}H NMR ($CDCl_3$, 400 MHz): δ=7.82 (d, *J*=8.8 Hz, 2H, Ar-H), 7.71 (d, *J*=8.8 Hz, 2H, Ar-H), 7.24 (d, *J*=5.3 Hz, 2H, Th-H_α), 7.20 (d, *J*=5.3 Hz, 2H, Th-H_β), ppm; ^{13}C NMR ($CDCl_3$, 101 MHz): δ= 143.6, 143.2, 134.1, 124.5, 122.3, 118.8,

118.6, 112.2, 109.0, ppm. GC/EI-MS (70 eV): t_R=23.9 min; *m/z* (%)=280 (100) $[M]^+$. CI-MS: *m/z* (%)=281 (100) $[M+H]^+$. Elemental analysis: calc. (%) for $C_{15}H_8N_2S_2$: C 64.26, H 2.88, N 9.99, S 22.87; found: C 64.34, H 2.98, N 10.00, S 22.93.

4-(Biphen-1,1-yl)-4*H*-dithieno[3,2-*b*:2',3'-*d*]pyrrole (3d): 4-Bromobiphenyl **2d** (137 mg, 0.59 mmol); LC: SiO_2, PE:DCM=8:2; DTP **3d** was isolated as a colourless solid. Yields: *1.* $Pd(OAc)_2$/ $t$$Bu_3P$: 130 mg, 0.39 mmol, 66 %; *2.* Pd_2dba_3/$t$$Bu_3P$: 161 mg, 0.49mmol, 87 %; *3.* CuI/m.w.: 127 mg, 0.38 mmol, 69 %. Mp 163.5-165.5 °C; ^1H NMR ($CDCl_3$, 400 MHz): δ=7.79-7.73 (m, 2H, Ar-H), 7.71-7.63 (m, 4H, Ar-H), 7.57-7.48 (m, 2H, Ar-H), 7.46-7.39 (m, 1H, Ar-H), 7.24 (d, *J*=5.3 Hz, 2H, Th-H_α), 7.21 (d, *J*=5.3 Hz, 2H, Th-H_β) ppm; ^{13}C NMR ($CDCl_3$, 101 MHz): δ= 144.0, 140.2, 139.1, 138.9, 129.0, 128.5, 127.6, 127.1, 123.6, 122.8, 117.2, 112.4 ppm. GC/EI-MS (70 eV): t_R=27.8 min; *m/z* (%)=331 (100) $[M]^+$. Elemental analysis: calc. (%) for $C_{20}H_{13}NS_2$: C 72.48, H 3.95, N 4.23; found: C 72.48, H 3.79, N 4.34.

4-(9,9-Dimethyl-9H-fluoren-2-yl)-4*H*-dithieno[3,2-*b*:2',3'-*d*]pyrrole (3e): 2-Bromo-9,9-dimethylfluorene **2e** (160 mg, 0.59 mmol); LC: SiO_2, PE:DCM=8:2; DTP **3e** was isolated as a colourless solid. Yields: *1.* $Pd(OAc)_2$/$t$$Bu_3P$: 134 mg, 0.36 mmol, 72 %; *2.* Pd_2dba_3/$t$$Bu_3P$: 194 mg, 0.52 mmol, 94 %; *3.* CuI/m.w.: 149 mg, 0.40 mmol, 72 %. Mp 159-160 °C; ^1H NMR ($CDCl_3$, 400 MHz): δ=7.85 (d, *J*=8.0 Hz, 1H, Ar-H), 7.79-7.74 (m, 1H, Ar-H), 7.65 (d, *J*=2.0 Hz, 1H, Ar-H), 7.57 (dd, *J*=8.1, 2.1 Hz, 1H, Ar-H), 7.50-7.44 (m, 1H, Ar-H), 7.37 (td, *J*=7.0, 1.4 Hz, 2H, Ar-H), 7.21 (d, *J* = 0.6 Hz, 4H, Th-$H_{\alpha\beta}$), 1.56 (s, 6H, CH_3)ppm; ^{13}C NMR ($CDCl_3$, 101 MHz): δ=155.6, 153.7, 144.2, 139.1, 138.5, 137.4, 127.5, 127.4, 123.6, 122.8, 121.7, 121.2, 120.1, 117.3, 117.0, 112.5, 47.2, 27.3 ppm; GC/EI-MS (70 eV): *m/z* (%)=371 (100) $[M]^+$; Elemental analysis: calc. (%) for $C_{23}H_{17}NS_2$: C 74.36, H 4.61, N 3.77; found: C 74.48, H 4.63, N 3.91.

2-(Biphen-1,1-yl)-4*H*-dithieno[3,2-*b*:2',3'-*d*]pyrrole (3f): 2-Bromo-1,1'-biphenyl **2f** (137 mg, 0.59 mmol); LC: SiO_2, PE:EA=85:15; DTP **3f** was isolated as a grey solid. Yields: *1.* $Pd(OAc)_2$/ $t$$Bu_3P$: 0 mg, 0 mmol, 0 %; *2.* Pd_2dba_3/$t$$Bu_3P$: 0 mg, 0 mmol, 0 %; *3.* CuI/m.w.: 62 mg, 0.18 mmol, 33 %. Mp 116.2-119.1 °C; ^1H NMR ($CDCl_3$, 400 MHz): δ=7.56-7.51 (m, 1H, Ar-H), 7.49-7.45 (m, 1H, Ar-H), 7.45-7.36 (m, 2H, Ar-H), 7.07-7.00 (m, 3H, Ar-H), 6.99-6.94 (m, 2H, Ar-H), 6.87 (d, *J*=5.3 Hz, 3H, Th-H_α), 6.57 (d, *J*=5.3 Hz, 3H, Th-H_β) ppm).; ^{13}C NMR ($CDCl_3$, 101 MHz): δ = 144.8, 138.4, 138.0, 136.8, 131.6, 128.5, 128.3, 128.0, 127.3, 122.7, 116.0, 112.1

ppm. HR-MS (FTICR-MALDI): *m/z*: calcd. for $C_{20}H_{13}NS_2$: 331.04894; found: 331.04839 $[M]^+$; *δm/m*=1.66 ppm.

4-(Naphthalen-2-yl)-4H-dithieno[3,2-*b*:2',3'-*d*]pyrrole (3g): 2-Bromonaphthalene **2g** (121 mg, 0.59 mmol); LC: SiO_2, PE:DCM=8:2; DTP **3g** was isolated as a colorless solid. Yields: *1.* $Pd(OAc)_2/tBu_3P$: 101 mg, 0.31 mmol, 59 %; *2.* Pd_2dba_3/tBu_3P: 151 mg, 0.49 mmol, 89 %; *3.* CuI/m.w.: 146 mg, 0.48 mmol, 87 %. Mp: 162-164 °C; ^1H NMR (400 MHz, $CDCl_3$) δ=8.03-7.97 (m, 2H, Ar-H), 7.95-7.85 (m, 2H, Ar-H), 7.75 (dd, *J*=8.6, 2.2 Hz, 1H, Ar-H), 7.61-7.50 (m, 2H, Ar-H), 7.24 (d, *J*=5.3 Hz, 2H, Th-$H_α$), 7.21 (d, *J*=5.3 Hz, 2H, Th-$H_β$) ppm; ^{13}C NMR (101 MHz, $CDCl_3$) δ=144.2, 137.4, 134.0, 131.6, 129.9, 128.0, 127.8, 127.2, 126.1, 123.7, 121.9, 120.0, 117.2, 112.4 ppm; GC/EI-MS: *m/z* (%) = 305 (100) $[M]^+$; HR-MS (FTCIR-APCI): m/z=$[M^+]$ calcd. for $C_{18}H_{11}NS_2$: 306.04069, found: 306.04064, *δm/m*=0.1 ppm.

4-(Anthracen-2-yl)-4H-dithieno[3,2-*b*:2',3'-*d*]pyrrole (3h): 2-Bromoanthracene **2h** (151 mg, 0.59 mmol); LC: SiO_2, PE:DCM=7:3; DTP **3h** was isolated as a bright yellow solid. Yields: *1.* $Pd(OAc)_2/tBu_3P$: 82 mg, 0.23 mmol, 41 %; *2.* Pd_2dba_3/tBu_3P: 120 mg, 0.34 mmol, 61 %; *3.* CuI/ m.w.: 89 mg, 0.25 mmol, 45 %. Mp 229-230 °C; ^1H NMR (400 MHz, $CDCl_3$) δ=8.47 (d, *J*=18.8 Hz, 2H, Ar-H), 8.20-8.11 (m, 2H, Ar-H), 8.03 (dd, *J*=10.0, 7.1 Hz, 2H, Ar-H), 7.77 (dd, *J*=9.0, 2.2 Hz, 1H, Ar-H), 7.55-7.47 (m, 2H, Ar-H), 7.30 (d, *J*=5.3 Hz, 2H, Th-$H_α$), 7.23 (d, *J*=5.4 Hz, 2H, Th-$H_β$) ppm; ^{13}C NMR (101 MHz, $CDCl_3$) δ=144.2, 136.8, 132.5, 131.8, 131.8, 130.5, 129.9, 128.4, 128.1, 126.7, 126.2, 126.0, 125.7, 123.8, 122.2, 119.3, 117.4, 112.5 ppm; HR-MS (FTCIR-APCI): m/z=$[M^+]$ calcd. for $C_{22}H_{13}NS_2$: 356.05622, found 356.05686, *δm/m*=1.7 ppm.

6-(4*H*-Dithieno[3,2-*b*:2',3'-*d*]pyrrol-4-yl)-1*H*,3*H*-benzo[*de*]isochromene-1,3-dione (3i): 4-Bromo-1,8-naphthalic anhydride **2i** (163 mg, 0.59 mmol); LC: SiO_2, DCM; DTP **3i** was isolated as an orange solid. Yields: *1.* $Pd(OAc)_2/tBu_3P$: 24 mg, 0.06 mmol, 11 %; *2.* Pd_2dba_3/tBu_3P: 38 mg, 0.10 mmol, 18 %; *3.* CuI/m.w.: 0 mg, 0 mmol, 0 %. Mp 305-310°C; ^1H NMR (TCE-d_2, 400 MHz): δ= 8.71-8.58 (m, 2H, Ar-H), 8.35 (dd, *J*=8.5, 1.1 Hz, 1H, Ar-H), 7.87 (d, *J*=7.9 Hz, 1H, Ar-H), 7.76 (dd, *J*=8.5, 7.3 Hz, 1H, Ar-H), 7.17 (d, *J*=5.4 Hz, 2H, Th-$H_α$), 6.77 (d, *J*=5.3 Hz, 2H, Th-$H_β$) ppm; ^{13}C NMR (TCE- d_2, 101 MHz): δ=160.9, 160.4, 145.4, 143.5, 134.7, 134.3, 132.5,

132.2, 128.3, 127.1, 125.1, 124.3, 119.5, 118.8, 117.3, 112.6 ppm; HR-MS (FTCIR-MALDI): *m/z*=[M+], calc. for $C_{20}H_9NO_3S_2$: 375.00238; found: 375.00303, *δm/m*=1.7 ppm.

6-(4*H*-Dithieno[3,2-*b*:2',3'-*d*]pyrrol-4-yl)-2-hexyl-1*H*-benzo[*de*]isoquinoline-1,3(2*H*)-dione (3j): *H*-DTP **1** (50 mg, 0.28 mmol), 6-Bromo-2-hexyl-1*H*-benzo[*de*]isoquinoline-1,3(2*H*)-dione **2j** (110 mg, 0.29 mmol); LC: SiO_2, PE:DCM=7:3; DTP **3j** was isolated as a yellow solid. Yields: *1.* $Pd(OAc)_2$/*t*Bu_3P: 82 mg, 0.18 mmol, 64 %; *2.* Pd_2dba_3/*t*Bu_3P: 119 mg, 0.26 mmol, 93 %; *3.* CuI/m.w.: 60 mg, 0.13 mmol, 45 %. Mp 161-163 °C (DSC); ^1H NMR ($CDCl_3$, 400 MHz): δ=8.75-8.66 (m, 2H, Ar-H_4, H_9), 8.24 (dd, *J*=8.5, 1.2 Hz, 1H, Ar-H_7), 7.87 (d, *J*=7.8 Hz, 1H, Ar-H_5), 7.74 (dd, *J*=8.5, 7.3 Hz, 1H, Ar-H_8), 7.19 (d, *J*=5.3 Hz, 2H, Th-H_α), 6.80 (d, *J*=5.4 Hz, 2H, Th-H_β), 4.27-4.14 (m, 2H, C*H*$_2$-H_α), 1.84-1.70 (m, 2H, C*H*$_2$-H_β), 1.45 (q, *J*=7.1 Hz, 2H, C*H*$_2$-H_γ), 1.40-1.24 (m, 4H, C*H*$_2$-$H_{\delta,\varepsilon}$), 0.96-0.84 (m, 3H, C*H*$_3$-$H_{\zeta,\zeta'}$) ppm; ^{13}C NMR ($CDCl_3$, 101 MHz): δ=164.1, 163.6, 145.6, 141.8, 132.1, 131.6, 130.4, 129.8, 127.5, 127.1, 124.3, 124.0, 123.5, 121.9, 118.1, 112.3, 40.8, 31.7, 28.2, 27.0, 22.7, 14.2 ppm; HR-MS (FTCIR-MALDI): *m/z*= [M+], calcd. for $C_{26}H_{22}N_2O_2S_2$: 458.11172; found: 458.11164; *δm/m*=0.18 ppm.

2-(2,6-Diisopropylphenyl)-8-(4H-dithieno[3,2-*b*:2',3'-*d*]pyrrol-4-yl)-1H-benzo[10,5]anthra[2,1,9-def]isoquioline-1,3(2H)-dione (*PDCI*-DTP) (3k): *H*-DTP **1** (50 mg, 0.28 mmol), 9-bromo-*N*-(2,6-diisopropylphenyl)-perylene-3,4-dicarboximide **2k** (164 mg, 0.29 mmol), LC: SiO_2, PE: DCM=9:1; *PDCI*-DTP **5m** was isolated as a dark red solid. Yields: *1.* $Pd(OAc)_2$/*t*Bu_3P: 128 mg, 0.20 mmol, 69 %; *2.* Pd_2dba_3/*t*Bu_3P: 108 mg, 0.16 mmol, 59 %; *3.* CuI/m.w.: 13 mg, 0.02 mmol, 9 %. Mp 253-254°C ^1H NMR (400 MHz, $CDCl_3$): δ=8.71 (dd, *J*=8.1, 1.6 Hz, 2H, H_4, H_{13}), 8.61-8.51 (m, 4H, H_5, H_6, H_{11}, H_{12}), 7.90 (dd, *J*=8.4, 0.9 Hz, 1H, H_9), 7.79 (d, *J*=8.0 Hz, 1H, H_7), 7.65 (dd, *J*=8.5, 7.5 Hz, 1H, H_{10}), 7.49 (dd, *J*=8.2, 7.3 Hz, 1H, p-Ph-*H*), 7.36 (d, *J*=7.8 Hz, 2H, m-Ph-*H*), 7.19 (d, *J*=5.3 Hz, 2H, Th-H_α), 6.87 (d, *J*=5.3 Hz, 2H, Th-H_β), 2.78 (q, *J*=6.9 Hz, 2H, *H*-CH_3), 1.20 (d, *J*=6.9 Hz, 12H, *CH*$_3$) ppm; ^{13}C NMR (101 MHz, $CDCl_3$) δ=164.1, 146.0, 145.8, 137.3, 132.3, 131.1, 130.7, 130.2, 130.0, 129.7, 129.5, 127.9, 126.9, 124.9, 124.7, 124.2, 124.0, 123.9, 121.5, 120.9, 120.8, 117.4, 112.5, 29.3, 24.2 ppm. HR-MS (FTCIR-MALDI): m/z=[M+], calcd. for $C_{42}H_{30}N_2O_2S_2$: 658.17432, found: 658.17325; *δm/m*=1.63 ppm.

2-(2,6-Diisopropylphenyl)-8-(2,6-dibromo-4H-dithieno[3,2-*b*:2',3'-*d*]pyrrol-4-yl)-1H-benzo-[10,5]anthra[2,1,9-*def*]isoquioline-1,3(2H)-dione (4): 511 mg (0.78 mmol) of **3k** was placed

in absolute THF (15 mL) under argon atmosphere and light exclusion and 283 mg (1.59 mmol) of NBS dissolved in THF (5 mL) was added and stirred for 18 h overnight at room temperature. For work-up, DCM (50 mL) was added to the mixture and washed with water (150 mL). The aqueous phase was counter-shaken twice with DCM (100 mL). After drying the organic phase over magnesium sulfate and removing the solvent, column chromatography was performed on flash silica gel with DCM. DTP **6** (608 mg, 0.74 mmol, 96 %) was obtained as an orange-red oil which solidified slowly in the refrigerator. Mp >400 °C; ^{1}H NMR (400 MHz, $CDCl_3$) δ=8.72 (dd, *J*=8.0, 2.0 Hz, 2H, H_4, H_{13}), 8.61-8.50 (m, 4H, H_5, H_6, H_{11}, H_{12}), 7.77-7.65 (m, 3H, H_7, H_9, H_{10}), 7.49 (t, *J*=7.8 Hz, 1H, p-Ph-*H*), 7.36 (d, *J*=7.8 Hz, 2H, m-Ph-*H*), 6.88 (s, 2H, Th-H_β), 2.78 (q, *J*=6.8 Hz, 2H, *H*-CH_3), 1.20 (d, *J*=6.9 Hz, 12H, CH_3) ppm; ^{13}C NMR (101 MHz, $CDCl_3$) δ=164.0, 145.8, 142.6, 137.2, 137.1, 136.6, 132.3, 132.3, 131.0, 130.6, 130.2, 130.1, 129.9, 129.7, 129.4, 128.4, 127.0, 126.1, 125.2, 124.7, 124.2, 123.6, 121.8, 121.7, 121.1, 121.0, 117.0, 115.5, 111.1, 68.1, 29.7, 29.3, 25.8, 24.2 ppm; HR-MS (FTCIR-MALDI): m/z= [M+], calcd. for $C_{42}H_{28}Br_2N_2O_2S_2$: 815.99385, found: 815.99209; *δm/m*=2.16 ppm.

2-(2,6-Diisopropylphenyl)-8-(4H-dithieno[3,2-*b*:2',3'-*d*]pyrrole-2,6-diyl)-1H-benzo[10,5]anthra[2,1,9-*def*]isoquioline-1,3(2H)-bis(thien-5-yl)bis(methaneylylidene)}dimalononitrile (6): Dibromo-DTP **4** (64 mg, 0.08 mmol) was submitted to argon atmosphere and added to **5** (88 mg, 0.20 mmol) dissolved in degassed dry toluene (2 mL). The mixture was degassed for 5 min by argon and $Pd[PPh_3]_4$ (5 mg, 4 µmol, 5 mol%) was added. The mixture was heated to 90 °C for 20 hrs. For workup, DCM (2 mL) and methanol (50 mL) were added to the batch and refrigerated overnight. The precipitated solid was filtered and washed with methanol. Column chromatography was then performed on flash silica gel using DCM:EA=99:1. Dyad **6** (43 mg, 0.04 mmol, 57 %) was isolated as a black solid. Mp >400°C, ^{1}H NMR (500 MHz, TCE-*d₂*, 355K) δ=8.66 (t, *J*=7.6 Hz, 2H, $H_{4,13}$), 8.58 (d, *J*=8.0 Hz, 1H, H_5), 8.57-8.48 (m, 3H, H_6, H_{11}, H_{12}), 7.77 (d, *J*=7.9 Hz, 1H, H_9), 7.66-7.59 (m, 4H, H-7, H10, DCV-*H*), 7.56 (d, *J*=4.2 Hz, 2H, Th-H_β), 7.42 (t, *J*=7.7 Hz, 1H, p-Ph-*H*), 7.31-7.21 (m, 4H, m-Ph-*H*, Th-H_β), 7.10 (s, 2H, Th-H_β), 2.74 (quint, *J*=6.8 Hz, 2H, *H*-CH_3), 1.16 (d, *J*=6.8 Hz, 12H, CH_3-*i*Pr) ppm; ^{13}C NMR (126 MHz, TCE-d_2, 355K) δ=163.9, 149.9, 149.7, 148.0, 146.2, 140.0, 137.0, 136.3, 135.8, 134.0, 132.1, 132.1, 131.6, 131.2, 130.8, 130.6, 129.9, 129.4, 128.9, 127.3, 126.0, 125.4, 124.9, 124.8, 124.1, 122.5, 122.3, 121.6, 121.5, 119.3, 114.3, 113.6, 111.5, 77.6, 77.3, 77.1, 76.7, 29.5, 24.2 ppm;

HR-MS (FTCIR-MALDI): m/z=[M^+], calcd. for $C_{58}H_{34}N_6O_2S_4$: 974.16206, found: 974.160075; *δm/m*=2.03 ppm.

2-(2,6-Diisopropylphenyl)-8-(4H-dithieno[3,2-b:2',3'-d]pyrrole-2,6-diyl)-1H-benzo[10,5]anthra[2,1,9-*def*]isoquioline-1,3(2H)-bis(3-hexylthien)-5-yl)]bis(methaneylylidene)}dimalononitrile (8): In a Schlenk tube DTP **4** (50 mg, 0.06 mmol) and 2-{[4-hexyl-5-(tributylstannyl)thien-2-yl]methylene}malononitrile **7** (82 mg, 0.15 mmol) were dissolved in toluene (1,5 mL) under argon atmosphere. Then, solid Pd[PPh_3]$_4$ (4 mg, 3,5 µmol) was added and the reaction mixture heated to 90 °C for 24 hrs. For work-up methanol (20 mL) was added to the reaction mixture and the black precipitate was filtered and washed with methanol. Then, the crude product was purified via column chromatography (SiO_2, dichloromethane) to yield D-A dyad **8** as black solid (22 mg, 0.02 mmol, 31 %). Mp >400°C; ^{1}H NMR (400 MHz, TCE- d_2): δ=8.67-8.62 (m, 2H, H_4, H_{13}), 8.58 (d, *J*=8.1 Hz, 1H, H_5), 8.55-8.48 (m, 3H, H_6, H_{11}, H_{12}), 7.79 (d, *J*=8.1 Hz, 1H, H_7), 7.70 (dd, *J*=8.3, 1.0 Hz, 1H, H_9), 7.63 (dd, *J*=8.4, 7.5 Hz, 1H, H_{10}), 7.59 (d, *J*=0.5 Hz, 2H, *H*-DCV), 7.49 (s, 2H, Th-H_β), 7.41 (t, *J*=7.8 Hz, 1H, *H*-p-Ph), 7.27 (d, *J*=7.8 Hz, 2H, *H*-m-Ph), 7.05 (s, 2H, Th-H_β), 2.83 (dd, *J*=8.5, 7.0 Hz, 4H, CH_2-$H_{\alpha,\alpha'}$), 2.78-2.68 (m, 2H, CH_3-*i*Pr), 1.73-1.59 (m, 4H, CH_2- $H_{\beta,\beta'}$), 1.42-1.22 (m, 12H, CH_2-$H_{\gamma,\gamma', \delta,\delta', \varepsilon,\varepsilon'}$), 1.15 (d, *J*=6.9 Hz, 12H, CH_3-*i*Pr), 0.85-0.77 (m, 6H, CH_3-H $_{\zeta,\zeta'}$) ppm; ^{13}C NMR (126 MHz, TCE-d_2) δ=164.0, 150.3, 147.1, 145.9, 144.9, 142.9, 141.2, 137.3, 136.8, 136.6, 134.4, 132.6, 132.4, 132.3, 131.4, 130.6, 130.5, 130.4, 129.7, 129.5, 128.7, 127.1, 125.8, 125.0, 124.3, 124.2, 121.9, 121.8, 121.5, 121.4, 120.6, 119.8, 114.9, 113.9, 112.8, 99.8, 31.8, 30.3, 29.7, 29.5, 29.4, 24.4, 22.9, 14.5 ppm; HR-MS (FTCIR-MALDI): m/z=[M^+] calcd. for $C_{70}H_{58}N_6O_2S_4$: 1142.34986, found: 1142.34945, *δm/m*= 0.35 ppm.

2-(3,4'-Dihexyl-5'-(trimethylstannyl)-(2,2'-bithien-5-yl)methyleneyl)malononitrile (10): 3,4'-Dihexyl-5'-(trimethylstannyl)-2,2'-bithiophen-5-carbaldehyde **9** (498 mg, 0.95 mmol) was dissolved in a mixture of dichloroethane (30 mL) and ethanol (15 mL). Solid malononitrile (313 mg, 4.74 mmol) and β-alanine (5 mg, 0.06 mmol) were added and the reaction mixture heated to 60 °C for 18 hrs. Subsequently, the mixture was diluted with dichloromethane (50 mL) and washed with water (100 mL). The aqueous phase was shaken twice with dichloromethane (50 mL, each), dried over magnesium sulfate, and the solvent evaporated. Then, the crude product was purified via column chromatography (Al_2O_3, activity grade II, DCM/PE,

1:1) to yield stannyl **10** as yellow, viscous oil (403 mg, 0.70 mmol, 74 %). ^{1}H NMR (400 MHz, $CDCl_3$) δ=7.68 (s, 1H, DCV-*H*), 7.51 (s, 1H, Th-H_4), 7.32 (s, 1H, Th-$H_{3'}$), 2.83-2.79 (m, 2H, C*H*$_2$-H_α), 2.62-2.58(m, 2H, C*H*$_2$-$H_{\alpha'}$), 1.66-1,62 (m, 4H, C*H*$_2$-$H_{\beta,\beta'}$), 1.38-1.31 (m, 12H, C*H*$_2$-$H_{\gamma,\gamma',\delta,\delta',\varepsilon,\varepsilon'}$), 0.91-0.88 (m, 6H, C*H*$_3$-$H_{\zeta,\zeta'}$), 0.42 (s, 9H, Sn(C*H*$_3$)$_3$) ppm; ^{13}C NMR (101 MHz, $CDCl_3$) δ= 152.1, 150.3, 142.0, 140.4, 139.2, 138.0, 132.6, 131.9, 130.8, 129.9, 32.8, 32.2, 31.9, 31.7, 30.5, 30.2, 29.9, 29.4, 29.3, 29.2, 22.7, 14.2, -7.6 ppm; HR-MS (FTCIR-MALDI): m/z=[M$^+$] calcd. for $C_{27}H_{38}N_2S_2Sn$: 574.14949, found: 574.14950, *δm/m*=0.02 ppm.

2-(2,6-Diisopropylphenyl)-8-(4H-dithieno[3,2-b:2',3'-d]pyrrole-2,6-diyl)-1H-benzo[10,5]anthra[2,1,9-*def*]isoquioline-1,3(2H)-bis(3,4'-dihexyl-[2,2'-bithien]-5',5-diyl)]bis(methaneylylidene)}dimalononitrile (11): In a Schlenk tube, 2-(2,6-diisopropylphenyl)-8-(2,6-dibromo-4H-dithieno[3,2-*b*:2′,3′-*d*]pyrrol-4-yl)-1H-benzo[10,5]anthra[2,1,9-*def*]isoquioline-1,3(2H)-dione **4** (20 mg, 0.02 mmol) and 2-(3,4'-dihexyl-5'-(trimethylstannyl)-(2,2'-bithien-5-yl)methylene-yl)malononitrile **10** (31 mg, 0.05 mmol) were dissolved in DMF (1,0 mL) under argon atmosphere. Then, solid Pd_2dba_3 (0.5 mg, 0.5 µmol) and P(o-tol)$_3$ (0,15 mg, 0.5µmol) were added and the reaction mixture heated to 85 °C for 24 hrs. For work-up methanol (20 mL) was added to the reaction mixture and the black precipitate was filtered and washed with methanol. Then, the crude product was purified via column chromatography (SiO_2, dichloromethane) to yield D-A dyad **11** as black solid (5.0 mg, 3.4 µmol, 14 %). Mp >400 °C; ^{1}H NMR (400 MHz, TCE-*d*$_2$): δ=8.74 (d, *J*=1.6 Hz, 1H, H_4), 8.72 (d, *J*=1.7 Hz, 1H, H_{13}), 8.66 (d, *J*=8.2 Hz, 1H, H_5), 8.61 (d, *J*=7.6 Hz, 1H, H_{12}), 8.58 (d, *J*=1.9 Hz, 1H, H_6), 8.56 (d, *J*=2.0 Hz, 1H, H_{11}), 7.93 (d, *J*=8.6 Hz, 1H, H_6), 7.87 (d, *J*=8.0 Hz, 1H, H_8), 7.74-7.68 (m, 1H, H_7), 7.68 (s, 2H, DCV-*H*), 7.51-7.49 (m, 1H, Ph-*H*), 7.49 (s, 2H, Th-H_4), 7.36 (d, *J*=7.8 Hz, 2H, Ph-*H*), 7.20 (s, 2H,Th-$H_{3'}$), 6.96 (s, 2H, Th-*H*β), 2.86-2.75 (m, 10H, C*H*$_2$-$H_{\alpha,\alpha'}$, C*H*$_3$-*i*Pr), 1.72-1.62 (m, 8H, C*H*$_2$-$H_{\beta,\beta'}$),1.32-1.24 (m, 24H, C*H*$_2$-$H_{\gamma,\gamma',\delta,\delta',\varepsilon,\varepsilon'}$), 1.19 (d, *J*=6.8 Hz, 12H, C*H*$_3$-*i*Pr), 0.85 (d, *J*=2.6 Hz, 12H, C*H*$_3$-$H_{\zeta,\zeta'}$) ppm. ^{13}C NMR (126 MHz, TCE-*d*$_2$) δ=164.1, 150.5, 145.9, 145.8, 144.2, 141.2, 141.0, 137.6, 135.5, 134.3, 132.3, 132.2, 131.9, 131.4, 129.6, 124.3, 121.7, 121.7, 121.3, 118.1, 115.0, 114.0, 111.4, 99.8, 75.4, 51.2. HR-MS (FTCIR-MALDI) m/z=[M$^+$] calcd. for $C_{90}H_{86}N_6O_2S_6$: 1475.51310, found: 1475.51202, *δm/m*=0.7 ppm.

4. ^{1}H-, ^{13}C-NMR spectra

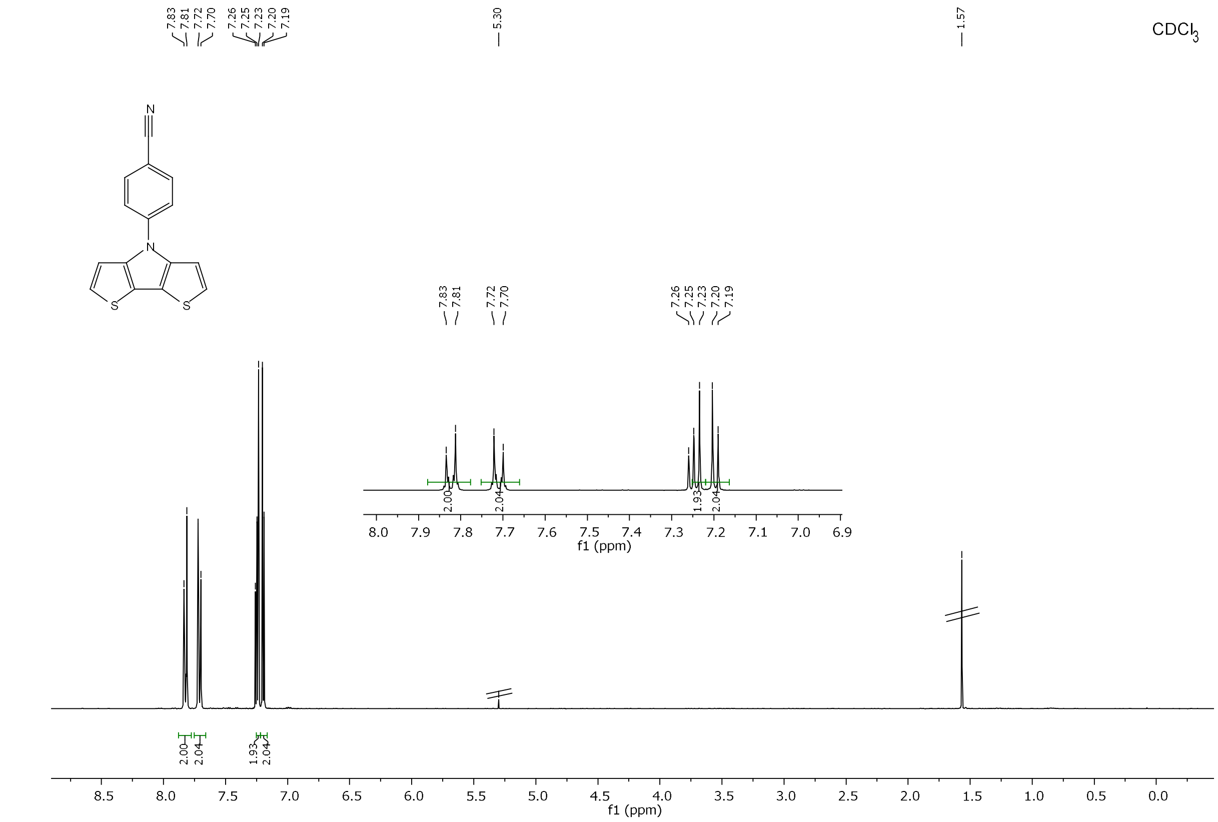

Figure S1a ^{1}H-NMR spectrum of **3c** measured in $CDCl_3$.

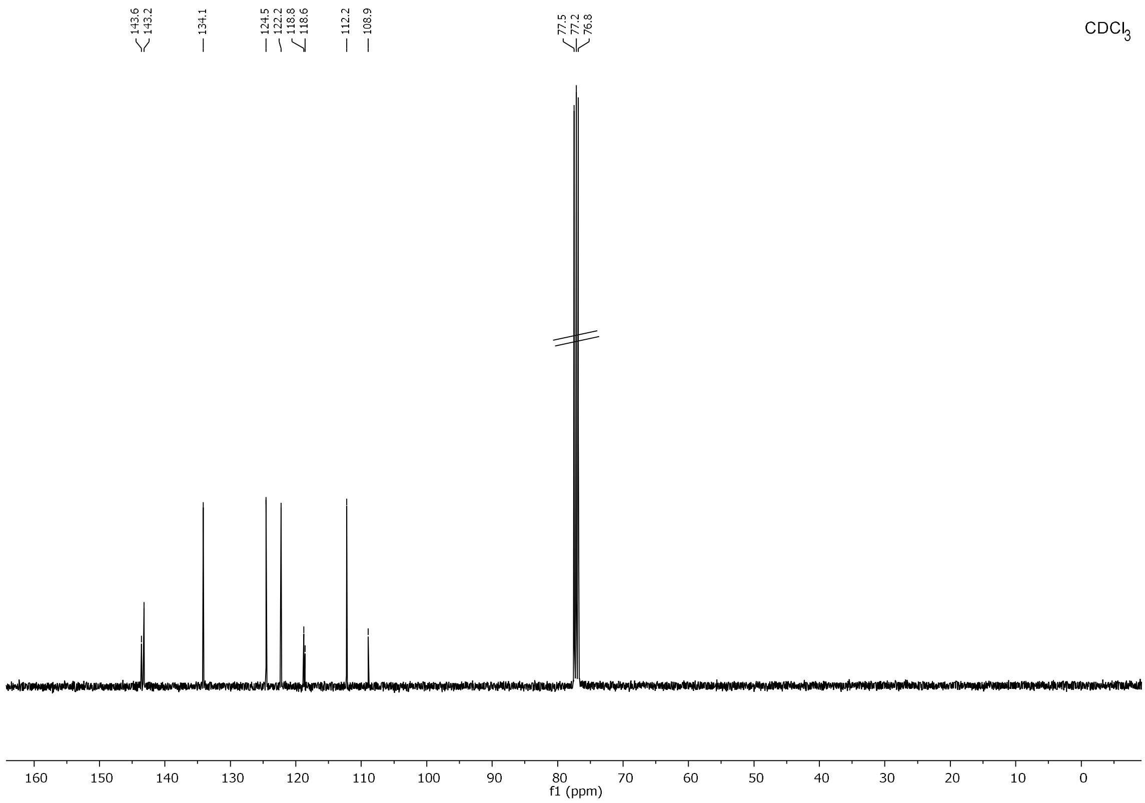

Figure S1b ^{13}C-NMR of spectrum of **3c** measured in $CDCl_3$.

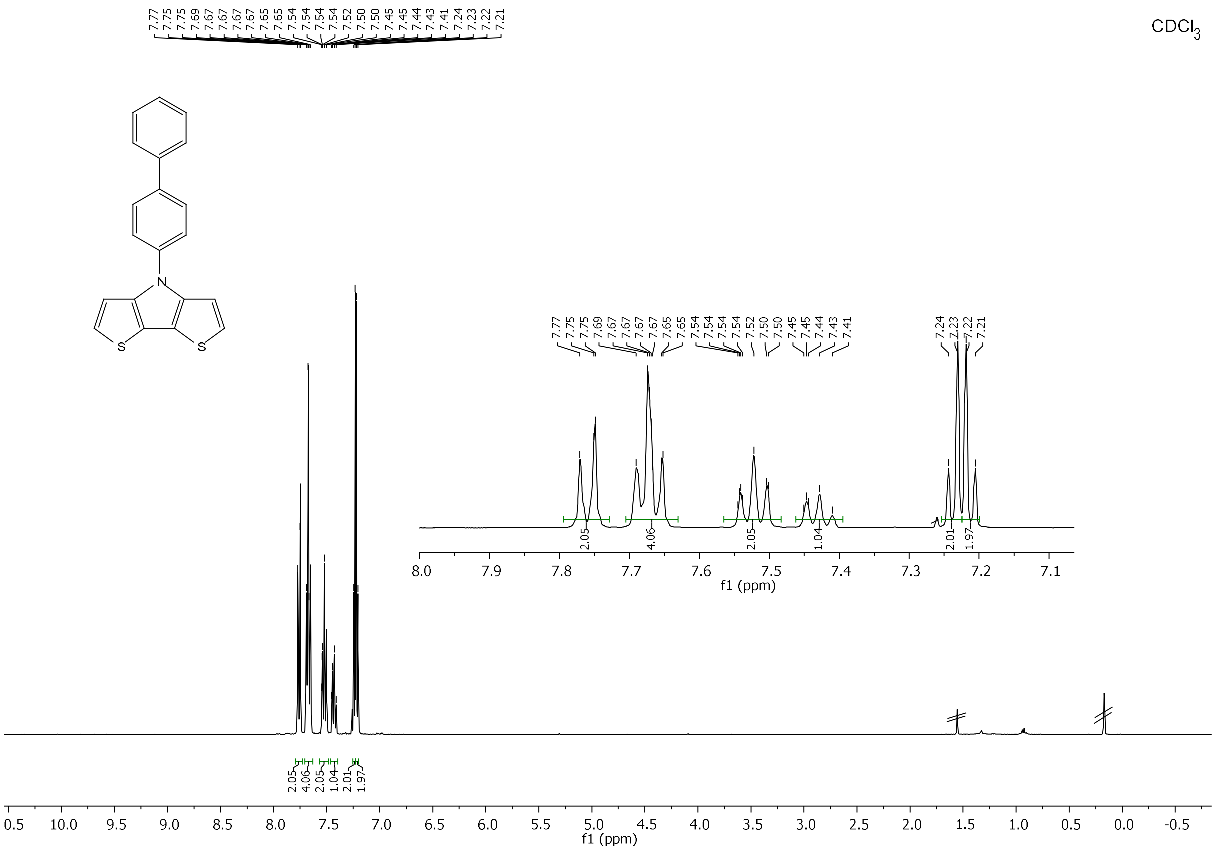

Figure S2a ^{1}H-NMR spectrum of **3d** measured in $CDCl_3$.

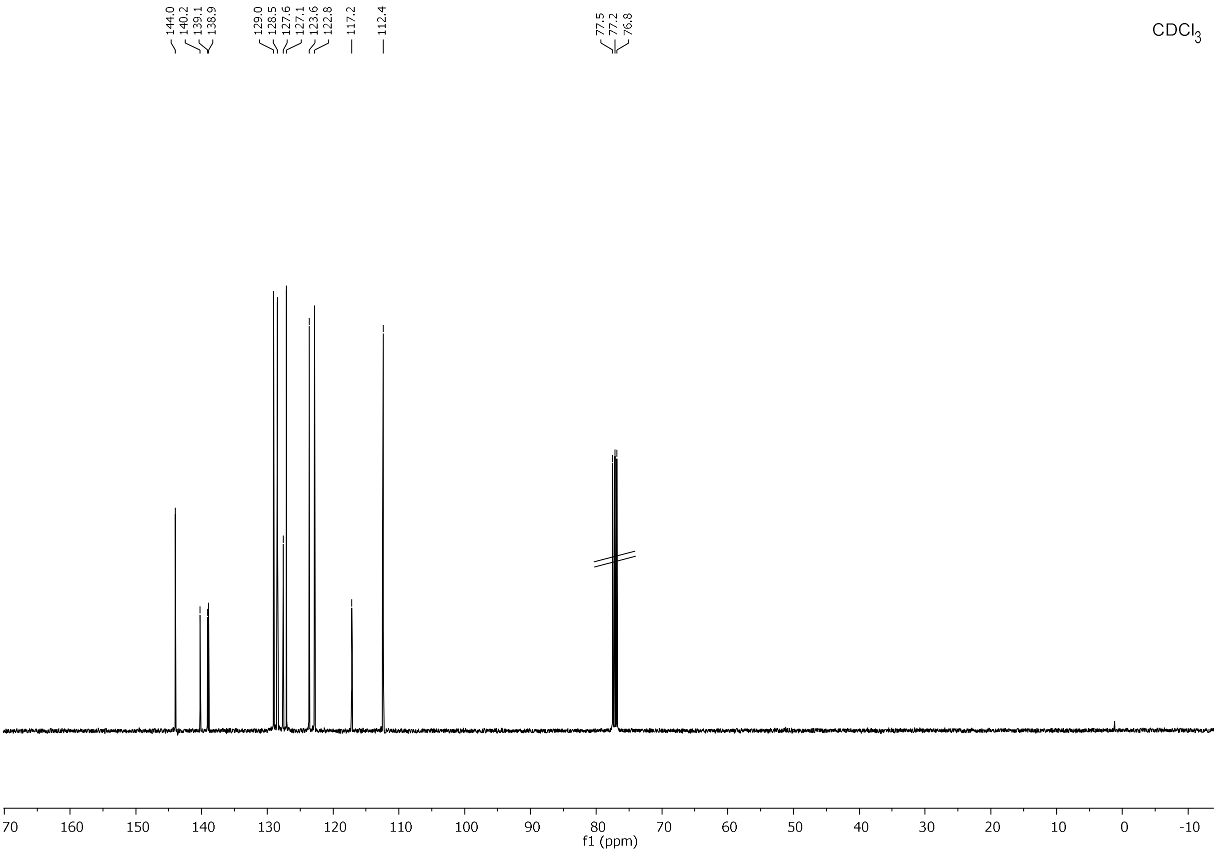

Figure S2b ^{13}C-NMR of spectrum of **3d** measured in $CDCl_3$.

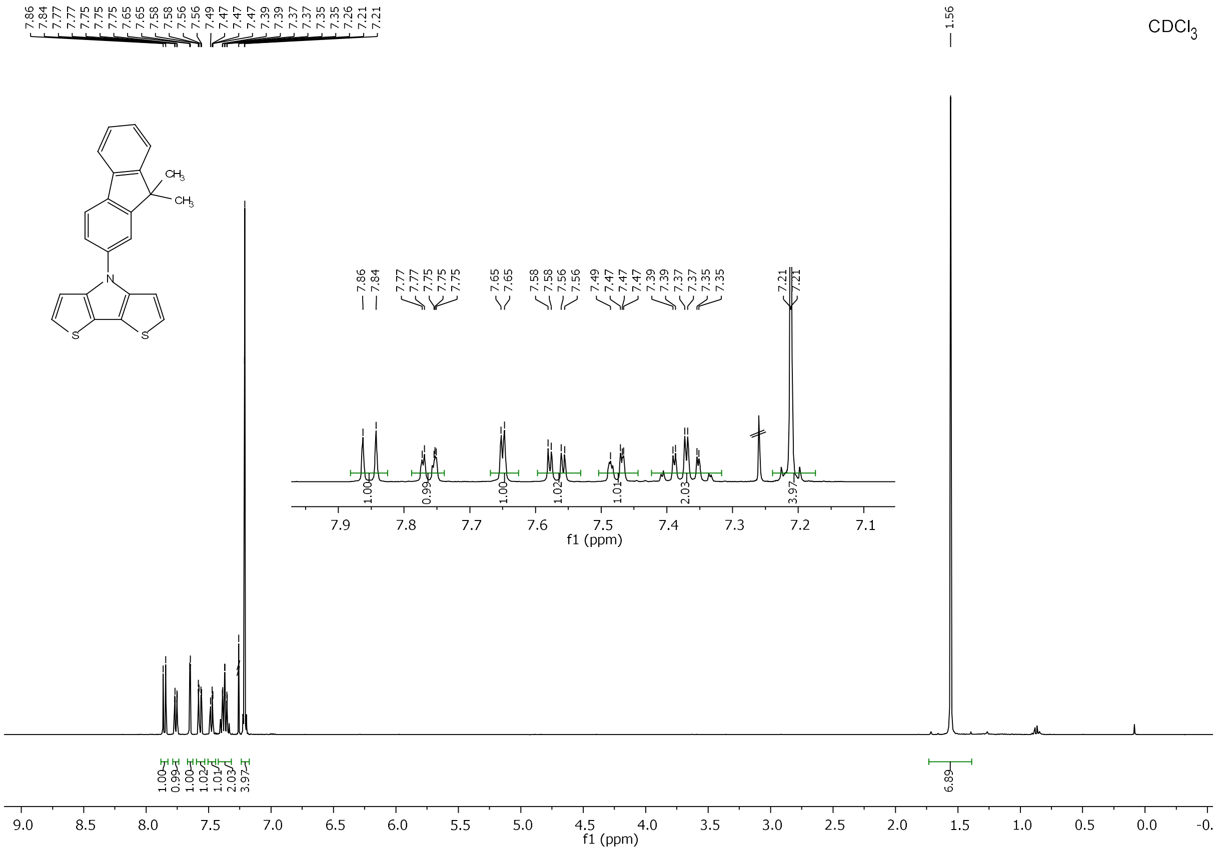

Figure S3a ^{1}H-NMR spectrum of **3e** measured in $CDCl_3$.

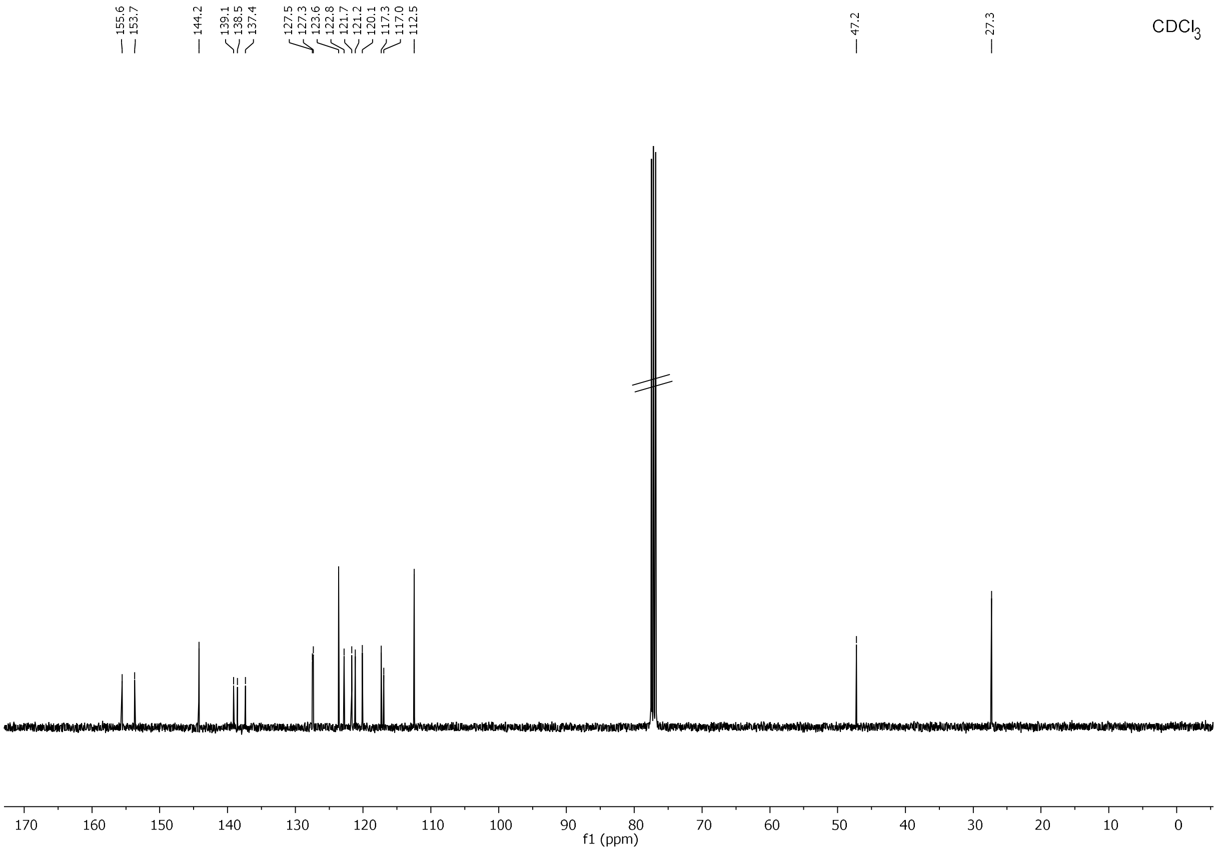

Figure S3b ^{13}C-NMR of spectrum of **3e** measured in $CDCl_3$.

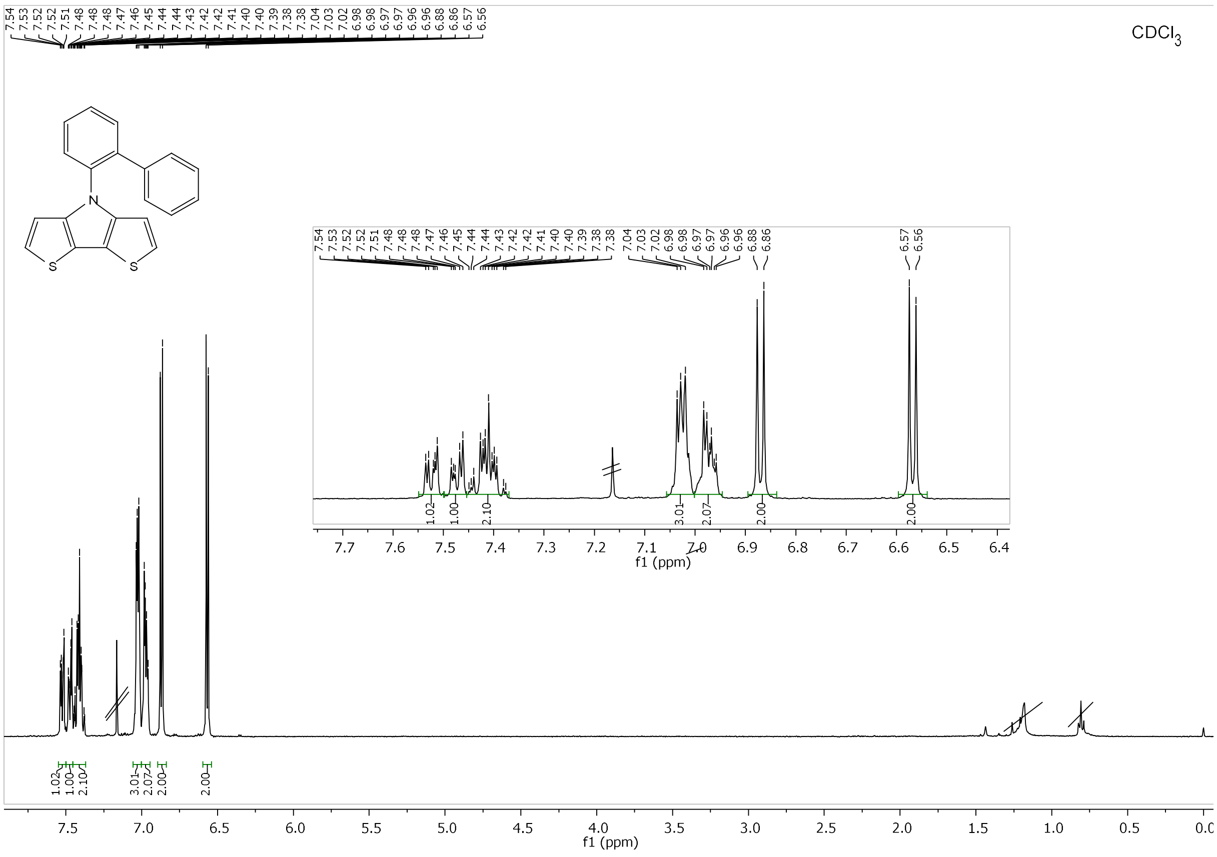

Figure S4a ^{1}H-NMR spectrum of **3f** measured in $CDCl_3$.

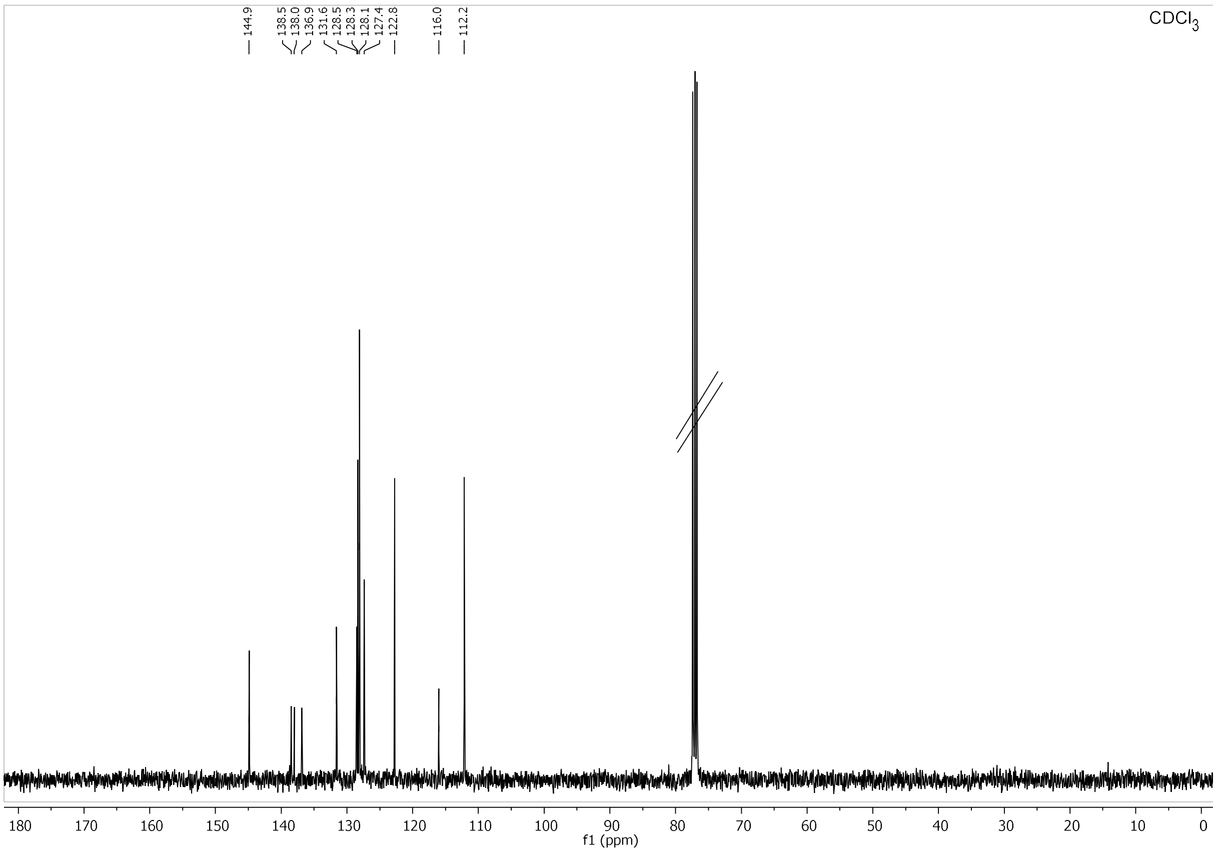

Figure S4b ^{13}C-NMR of spectrum of **3f** measured in $CDCl_3$.

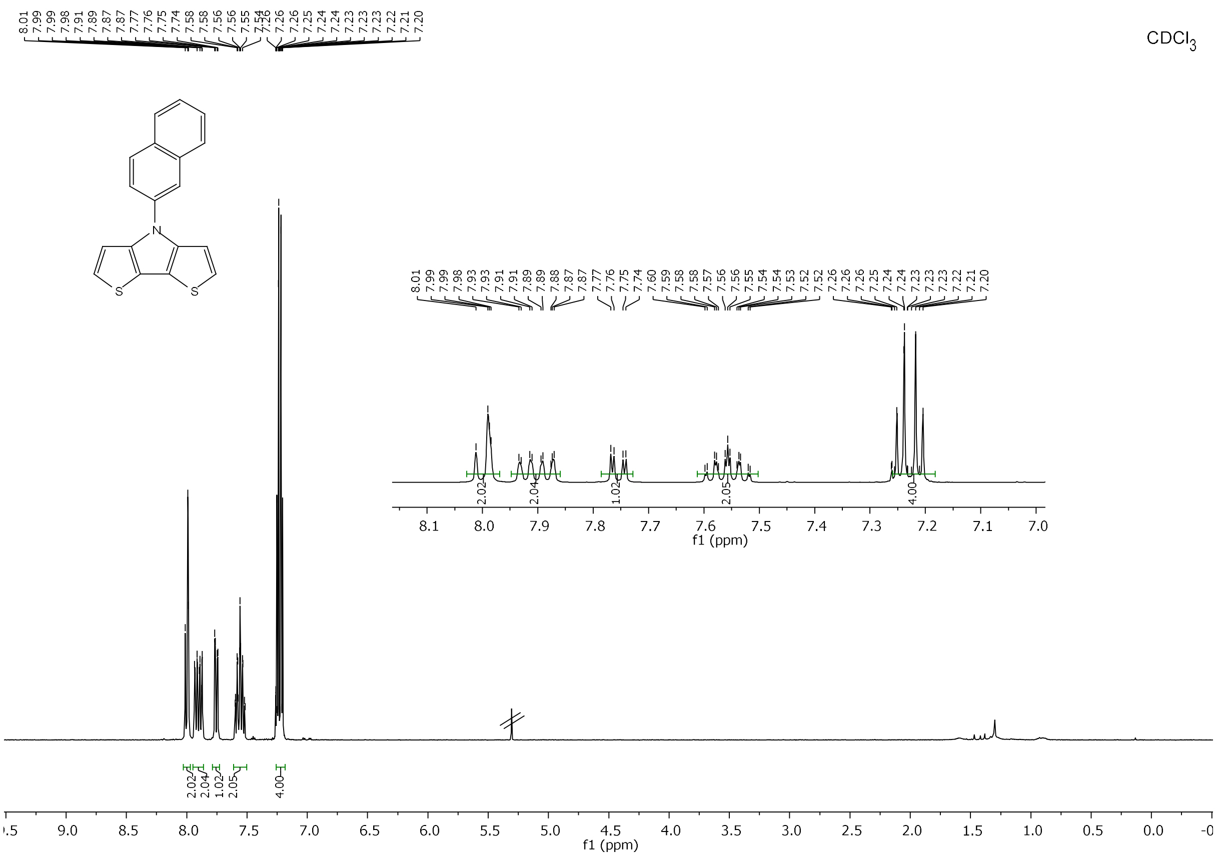

Figure S5a ^{1}H-NMR spectrum of **3g** measured in $CDCl_3$.

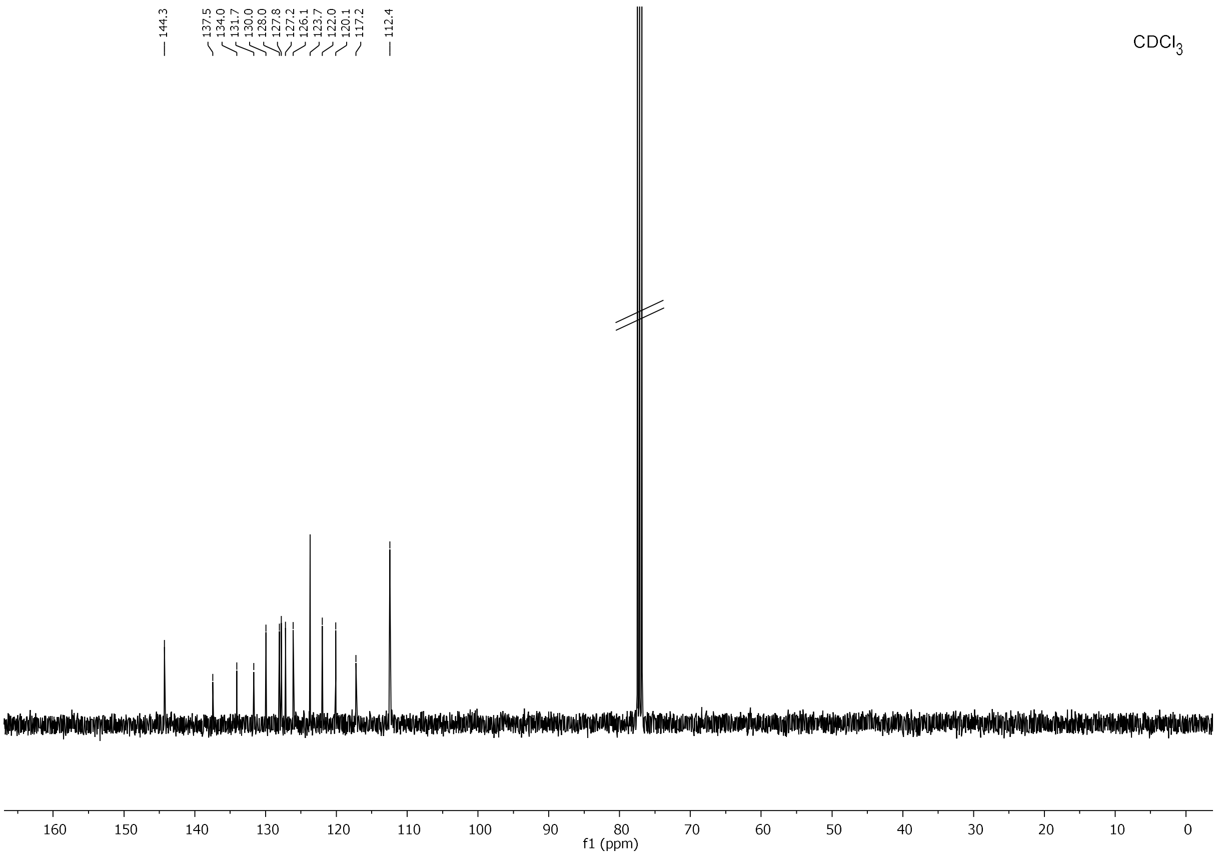

Figure S5b ^{13}C-NMR of spectrum of **3g** measured in $CDCl_3$.

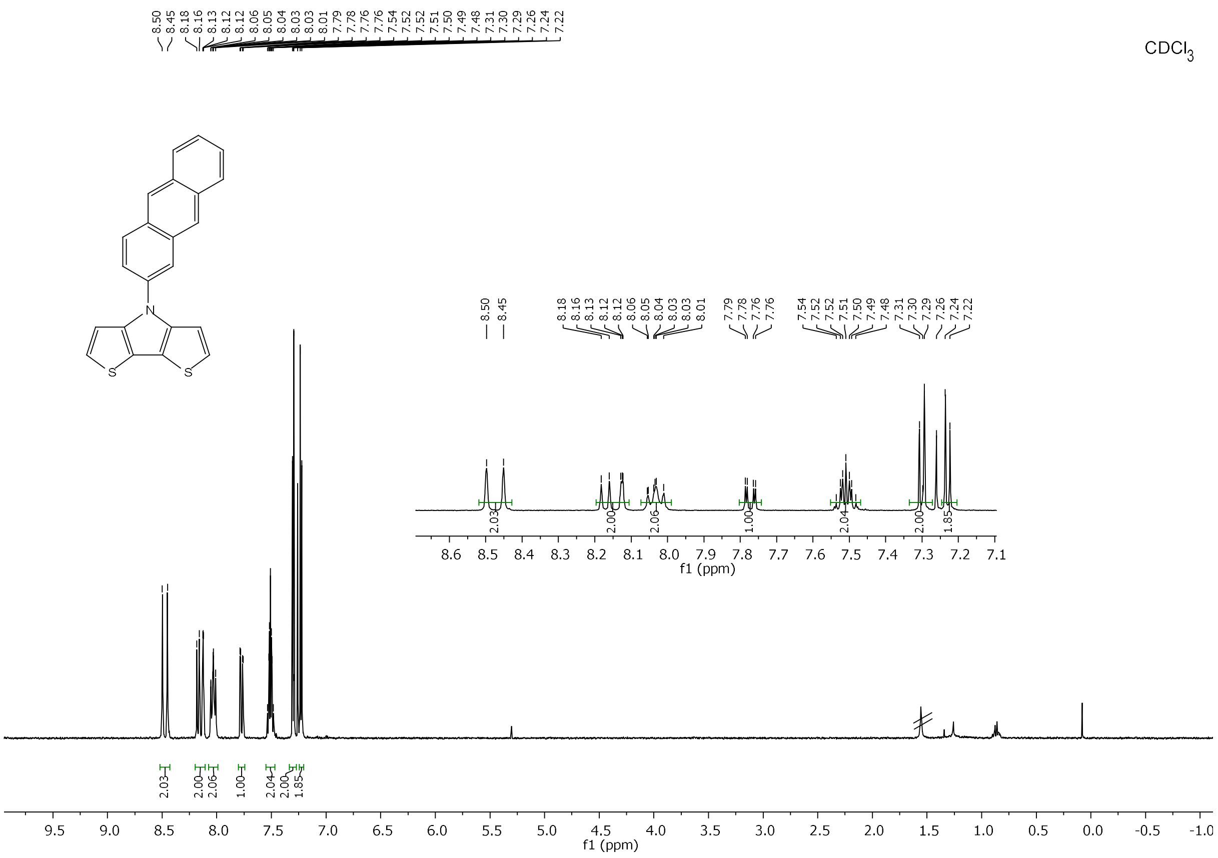

Figure S6a ^{1}H-NMR spectrum of **3h** measured in $CDCl_3$.

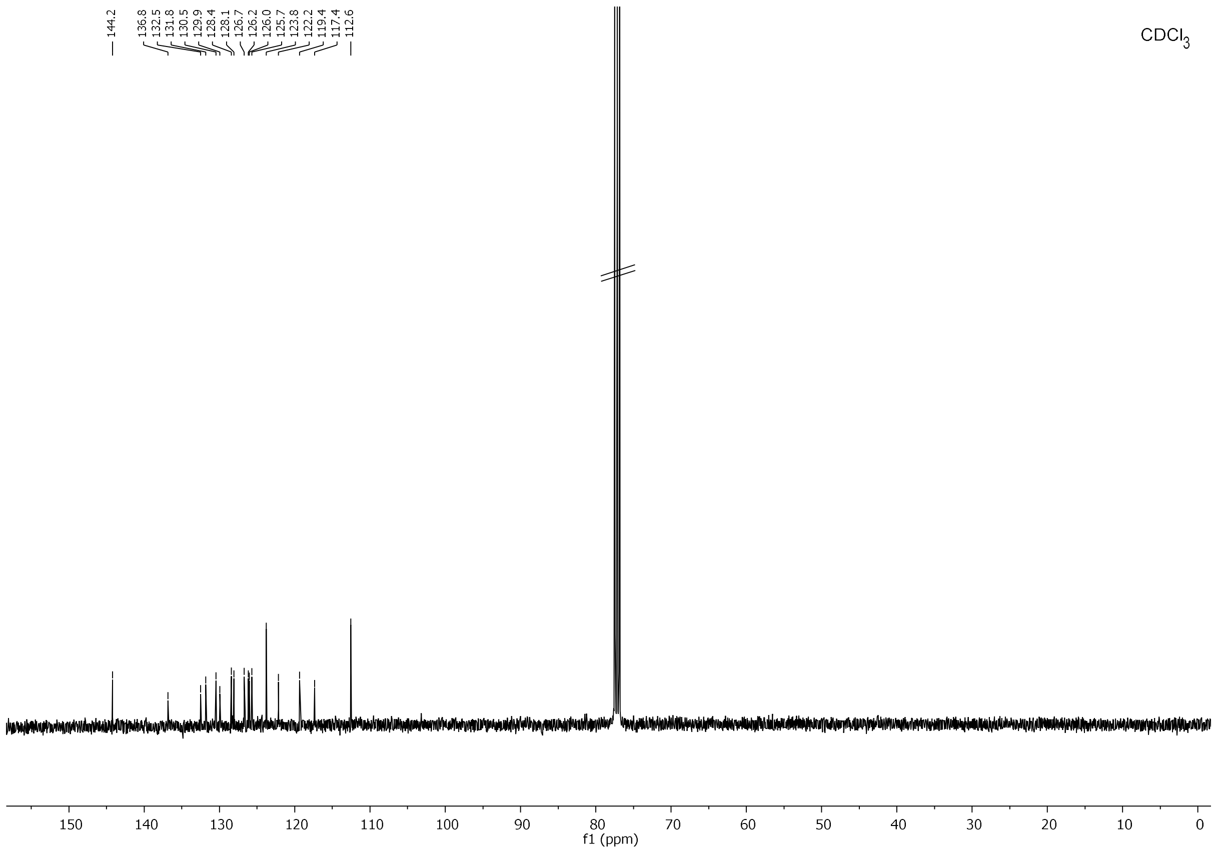

Figure S6b ^{13}C-NMR of spectrum of **3h** measured in $CDCl_3$.

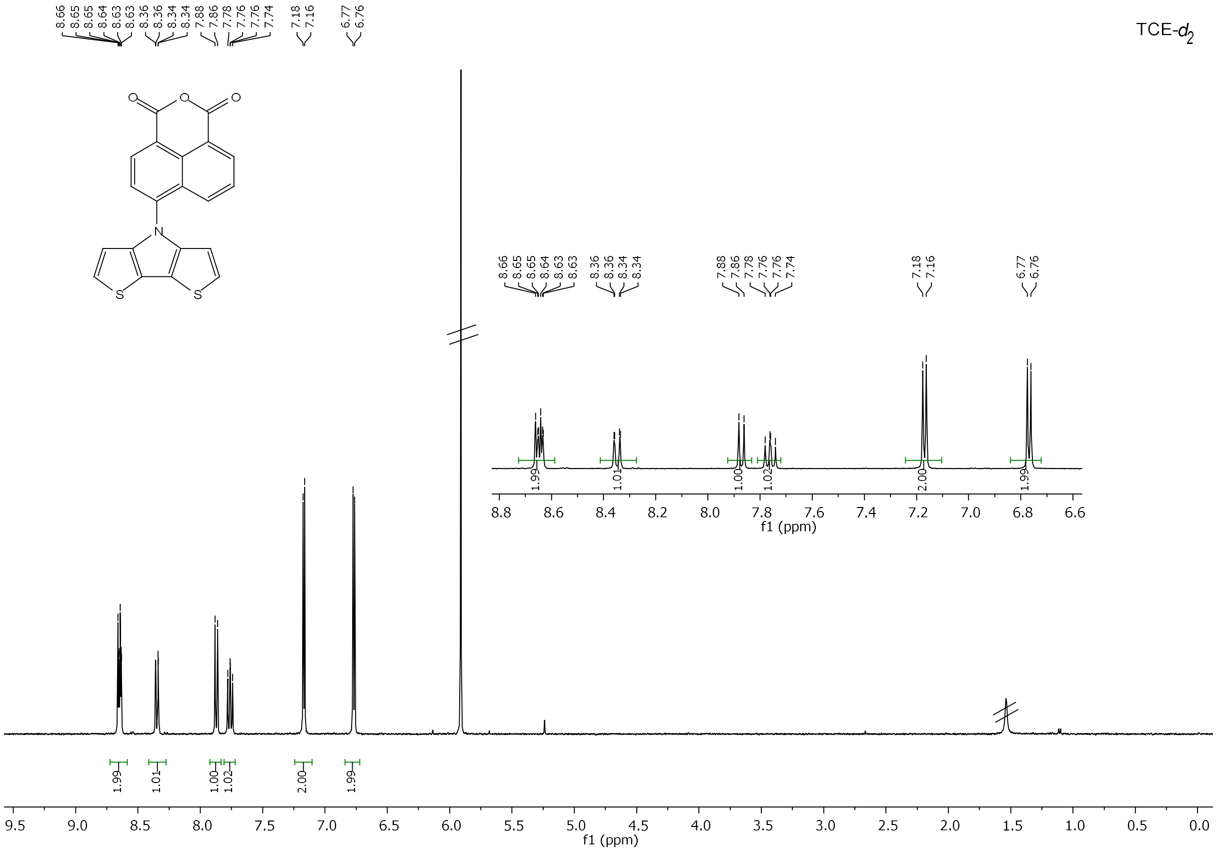

Figure S7a ^{1}H-NMR spectrum of **3i** measured in TCE-*d₂*.

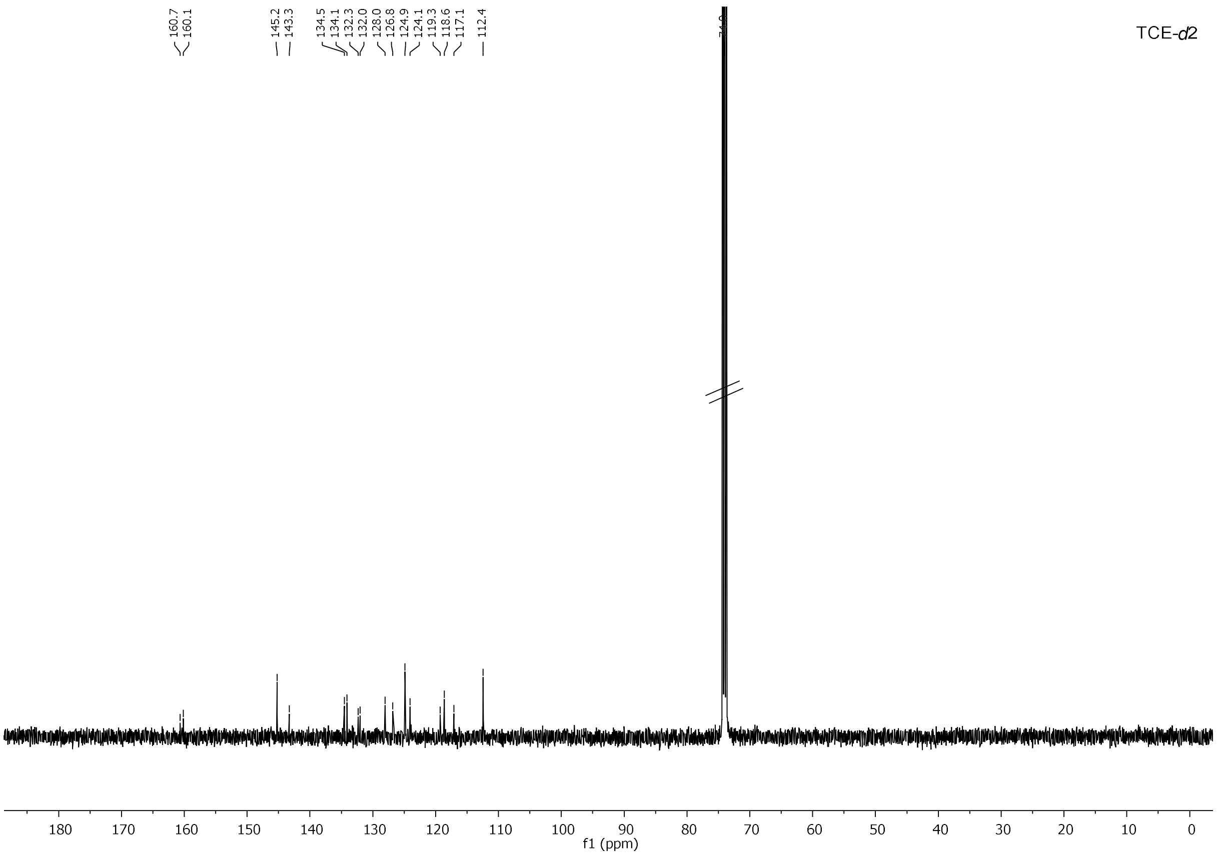

Figure S7b ^{13}C-NMR of spectrum of **3i** measured in TCE-d_2.

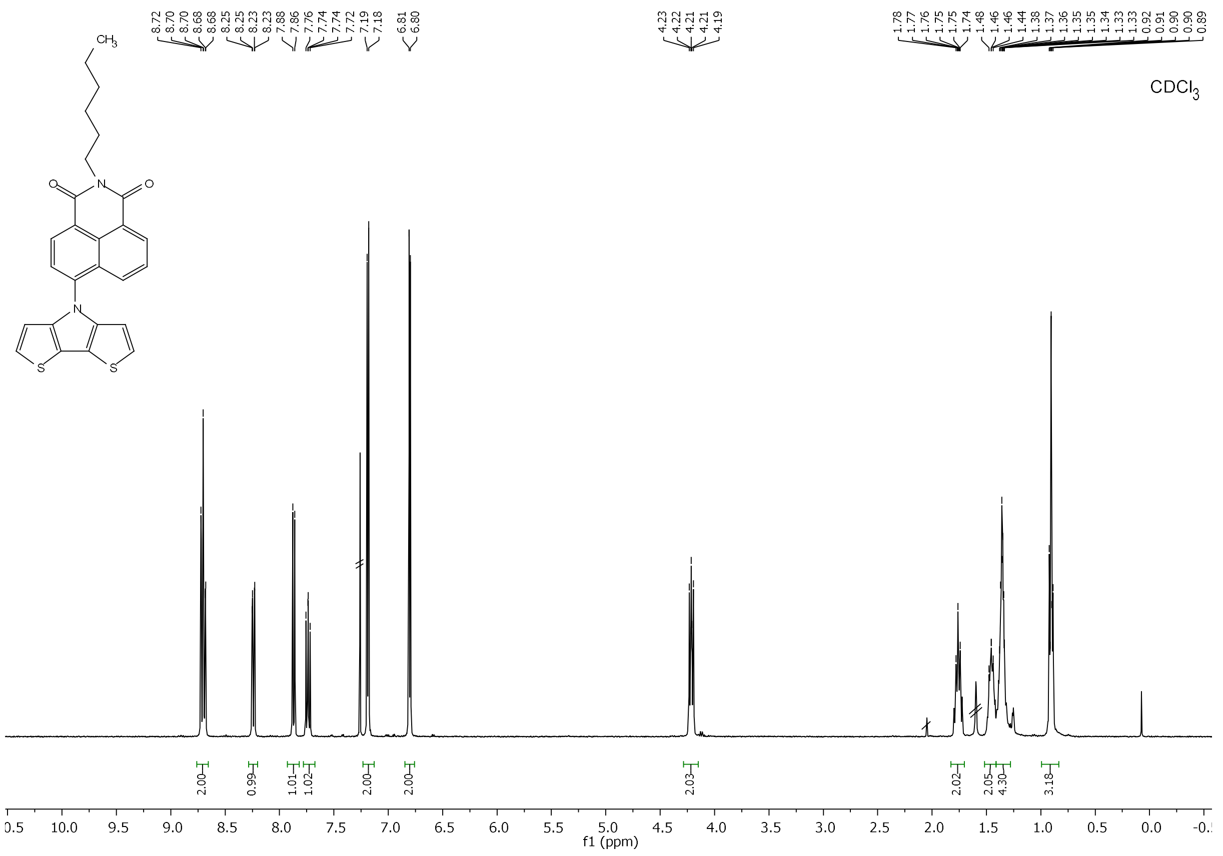

Figure S8a ^{1}H-NMR spectrum of **3j** measured in CDCl₃.

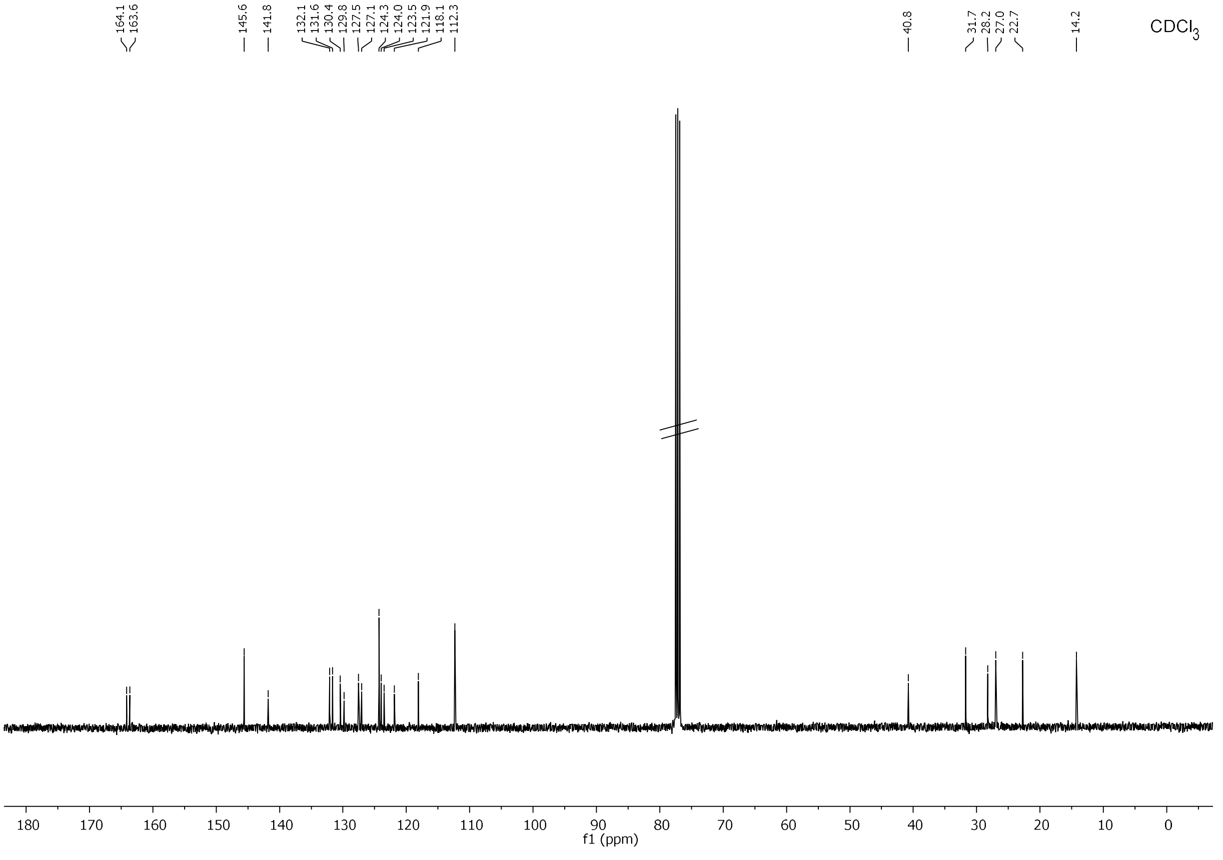

Figure S8b ^{13}C-NMR of spectrum of **3j** measured in CDCl₃.

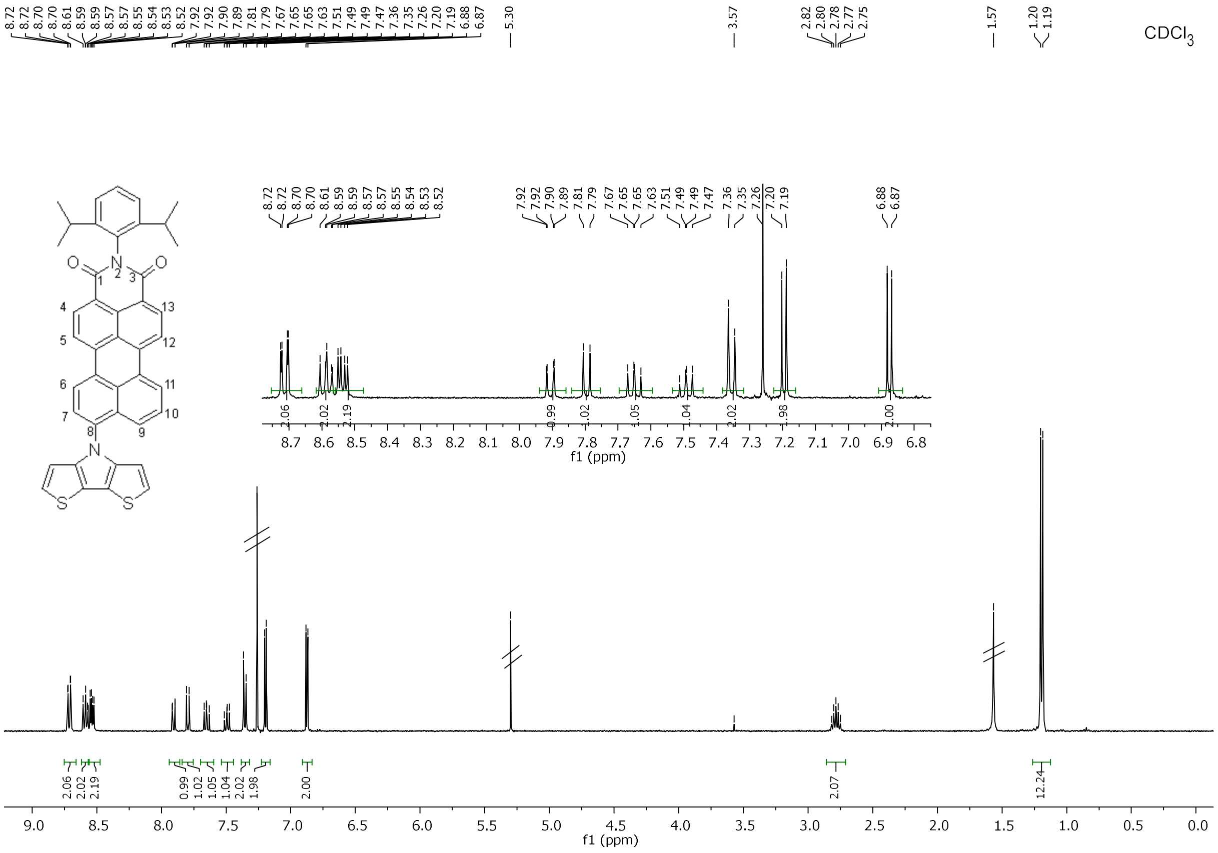

Figure S9a ^{1}H-NMR spectrum of **3k** measured in $CDCl_3$.

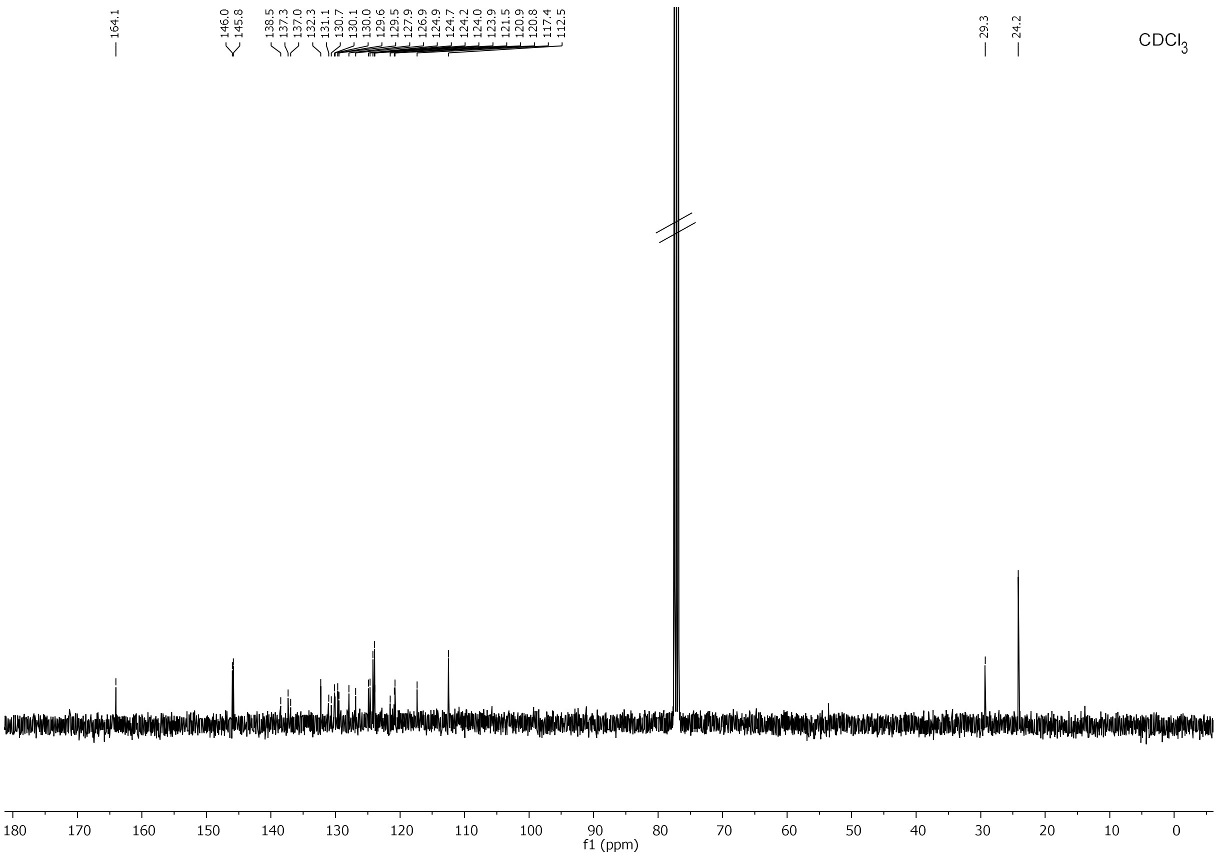

Figure S9b ^{13}C-NMR of spectrum of **3k** measured in $CDCl_3$.

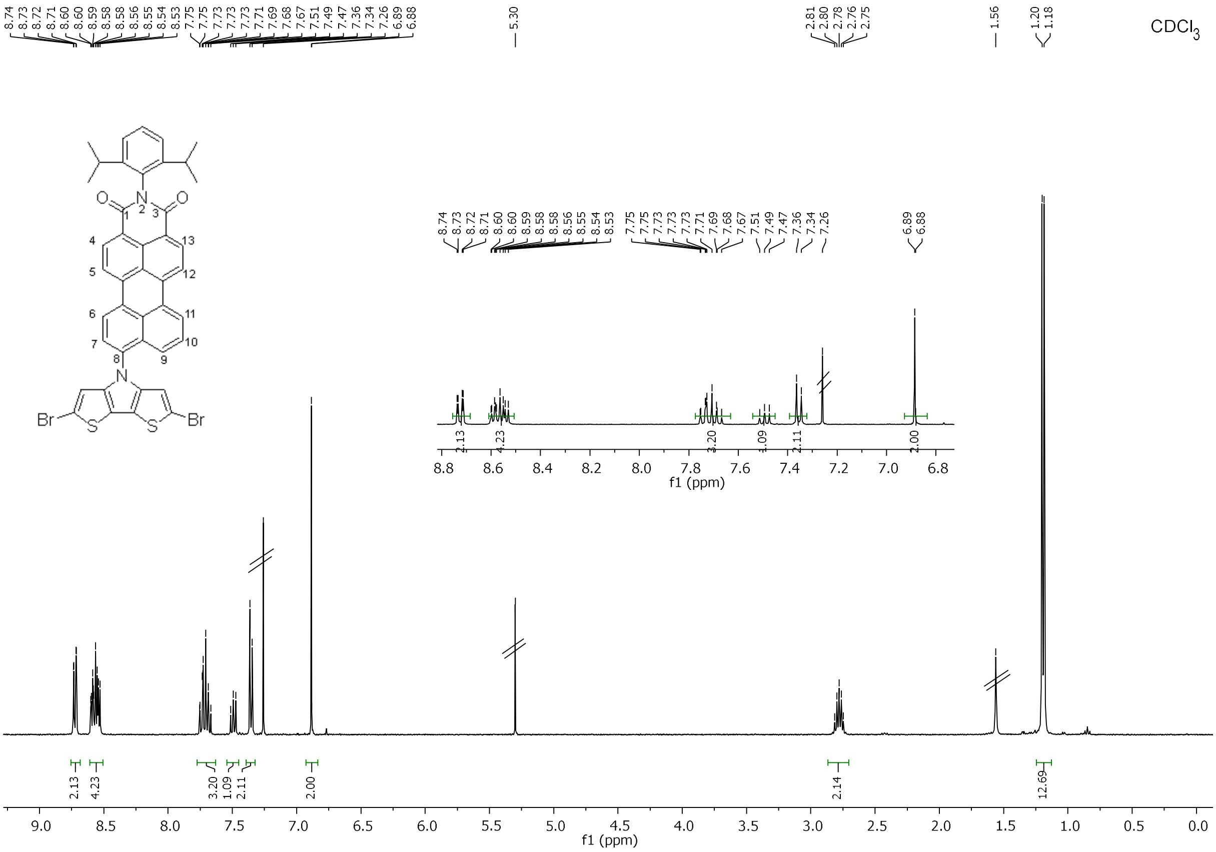

Figure S10a ^{1}H-NMR spectrum of **4** measured in $CDCl_3$.

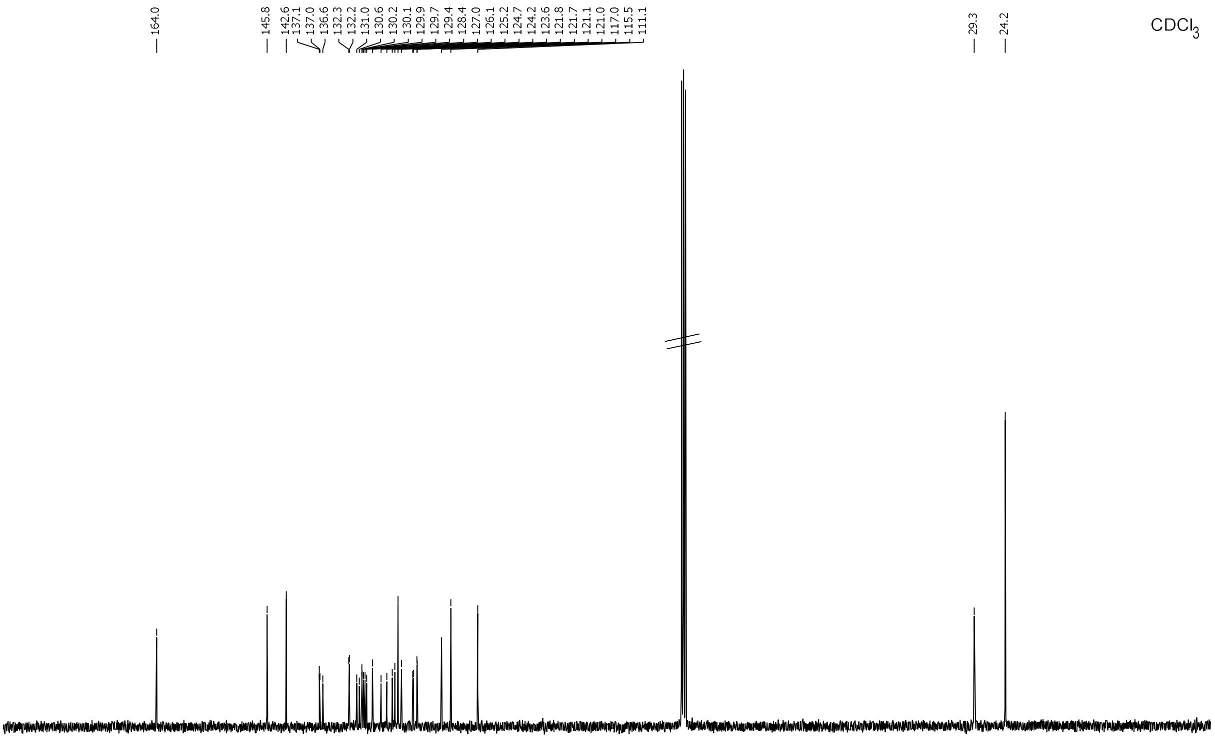

Figure S10b ^{13}C-NMR of spectrum of **4** measured in $CDCl_3$.

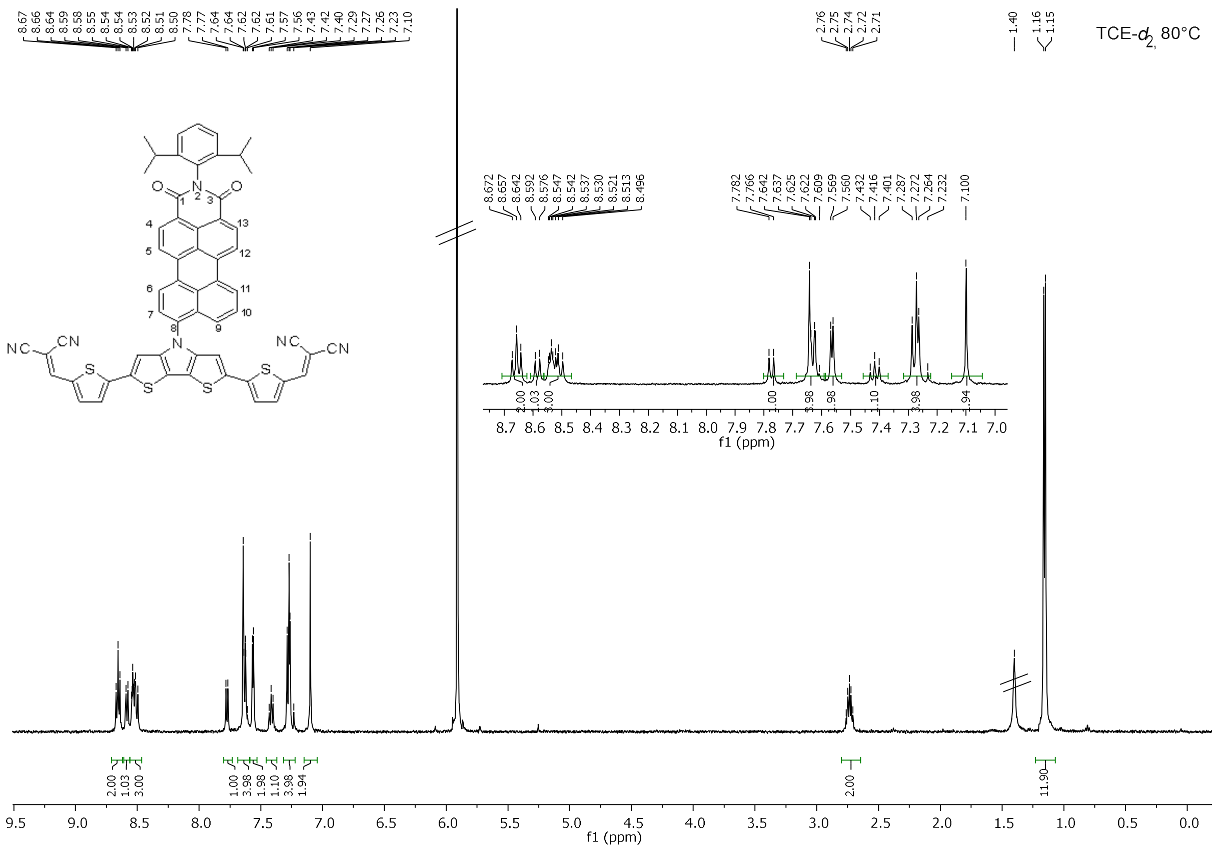

Figure S11a ^{1}H-NMR spectrum of **6** measured in TCE-d_2 (355 K).

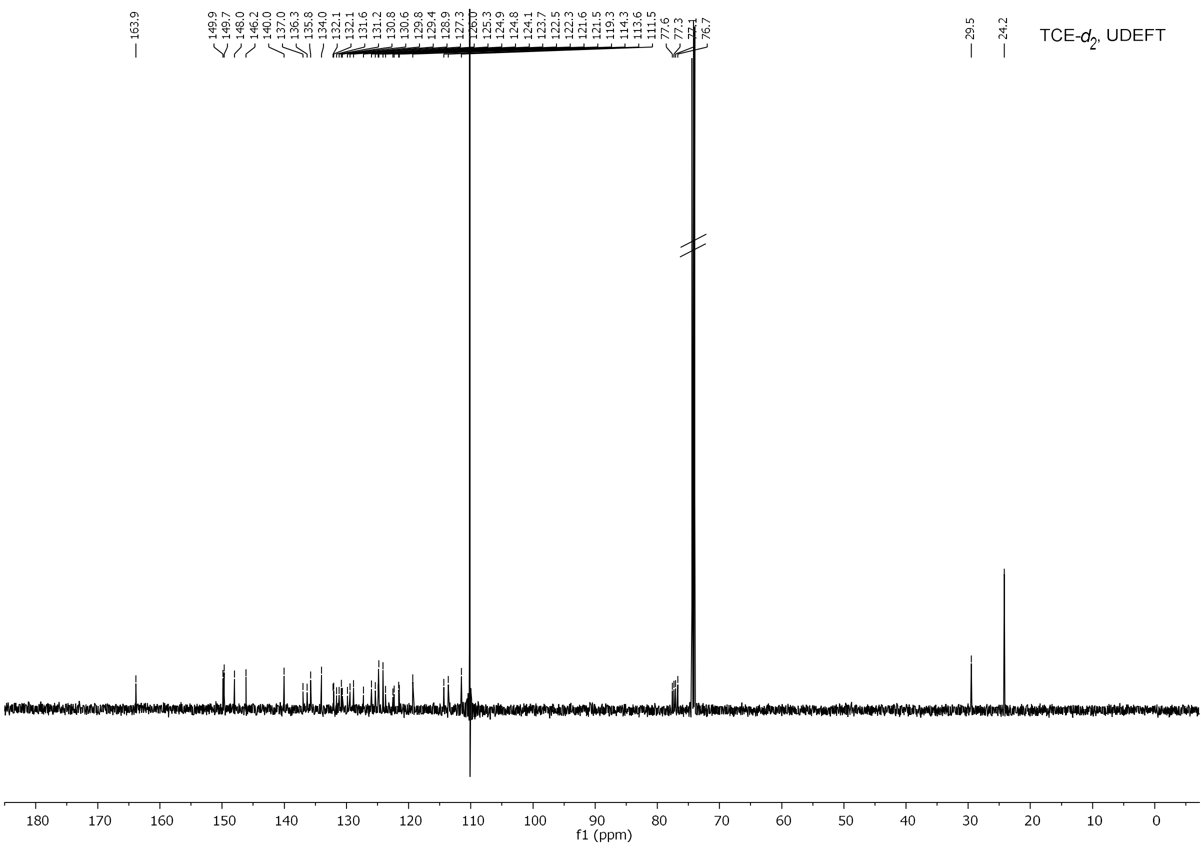

Figure S11b ^{13}C-NMR of spectrum of **6** measured in TCE-d_2 (355 K).

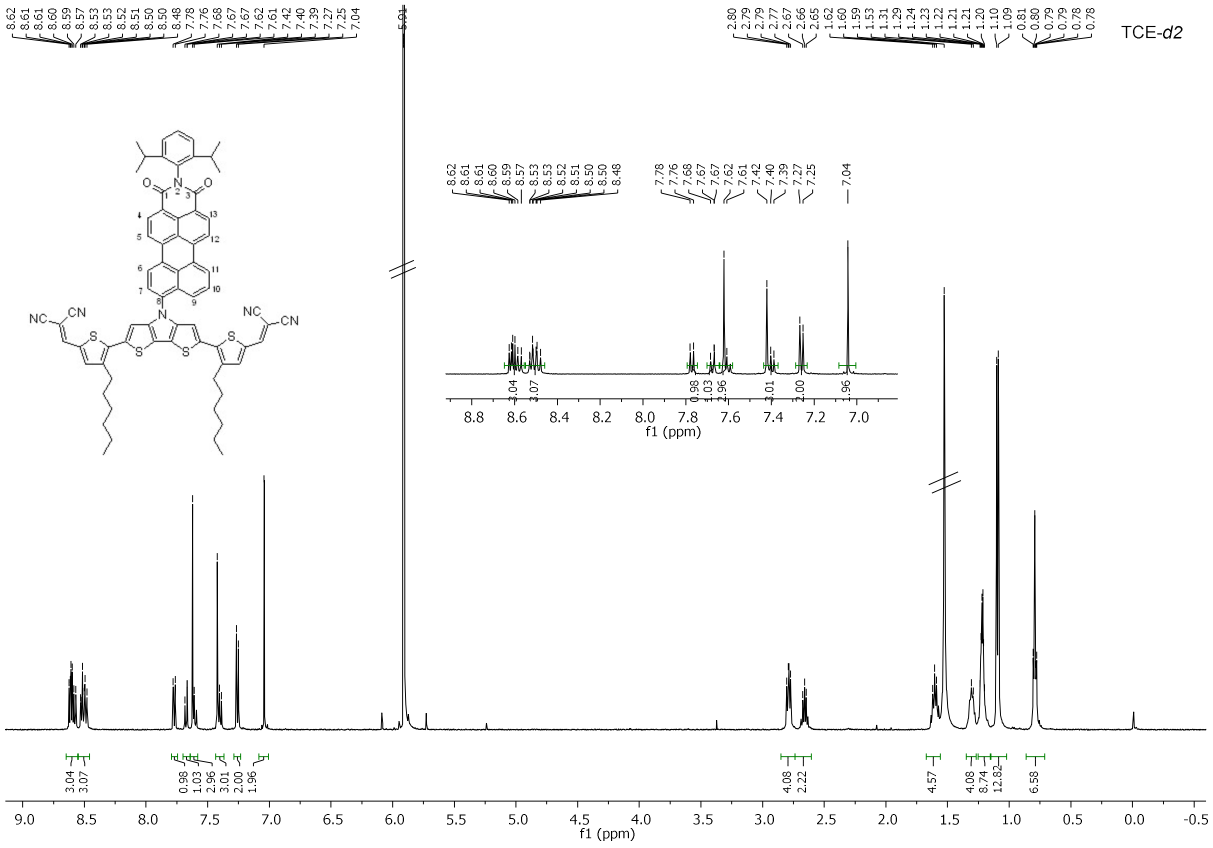

Figure S12a ^{1}H-NMR spectrum of **8** measured in TCE-d_2.

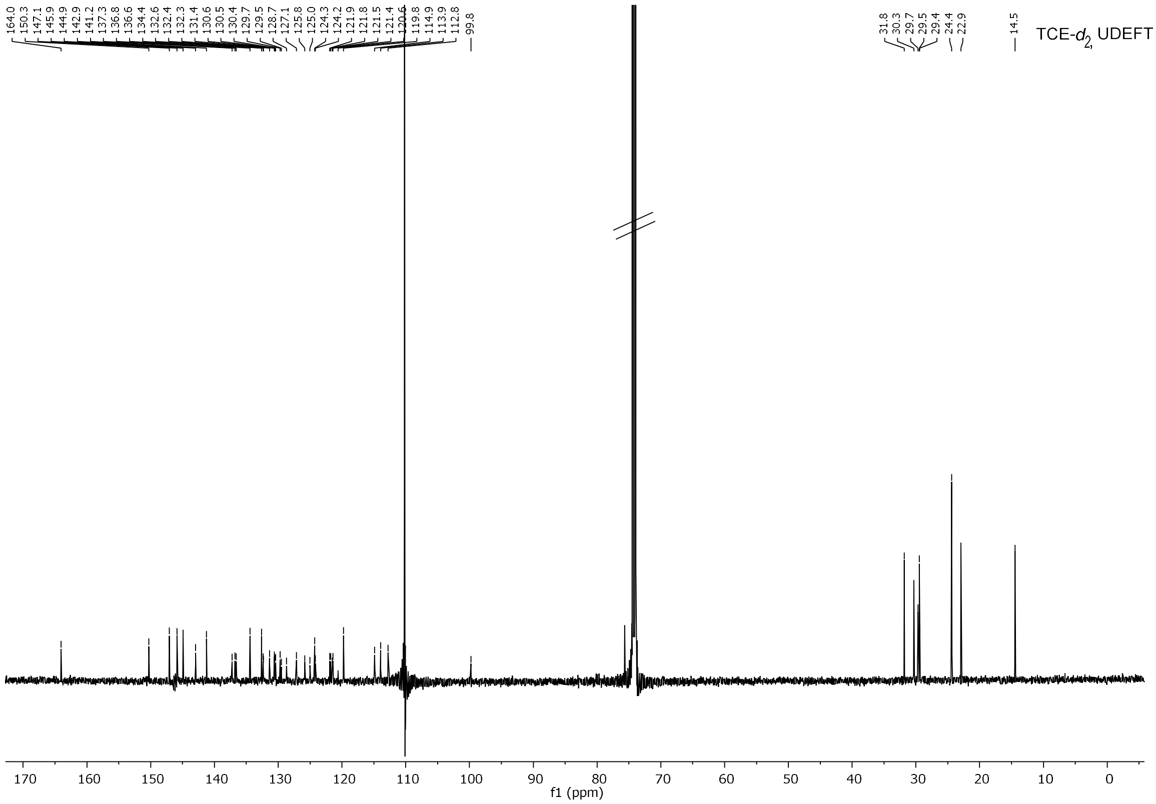

Figure S12b ^{13}C-NMR of spectrum of **8** measured in TCE-d_2.

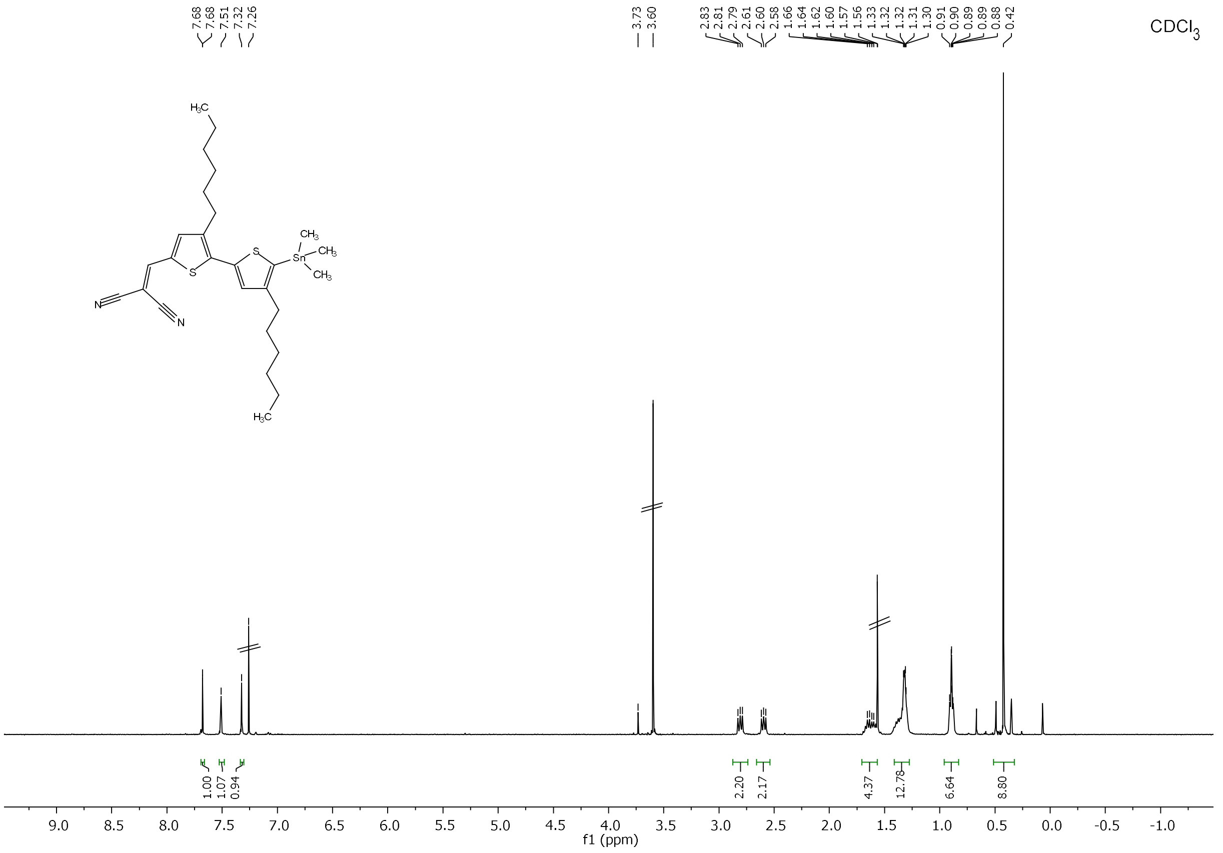

Figure S13a ^{1}H-NMR spectrum of **10** measured in $CDCl_3$.

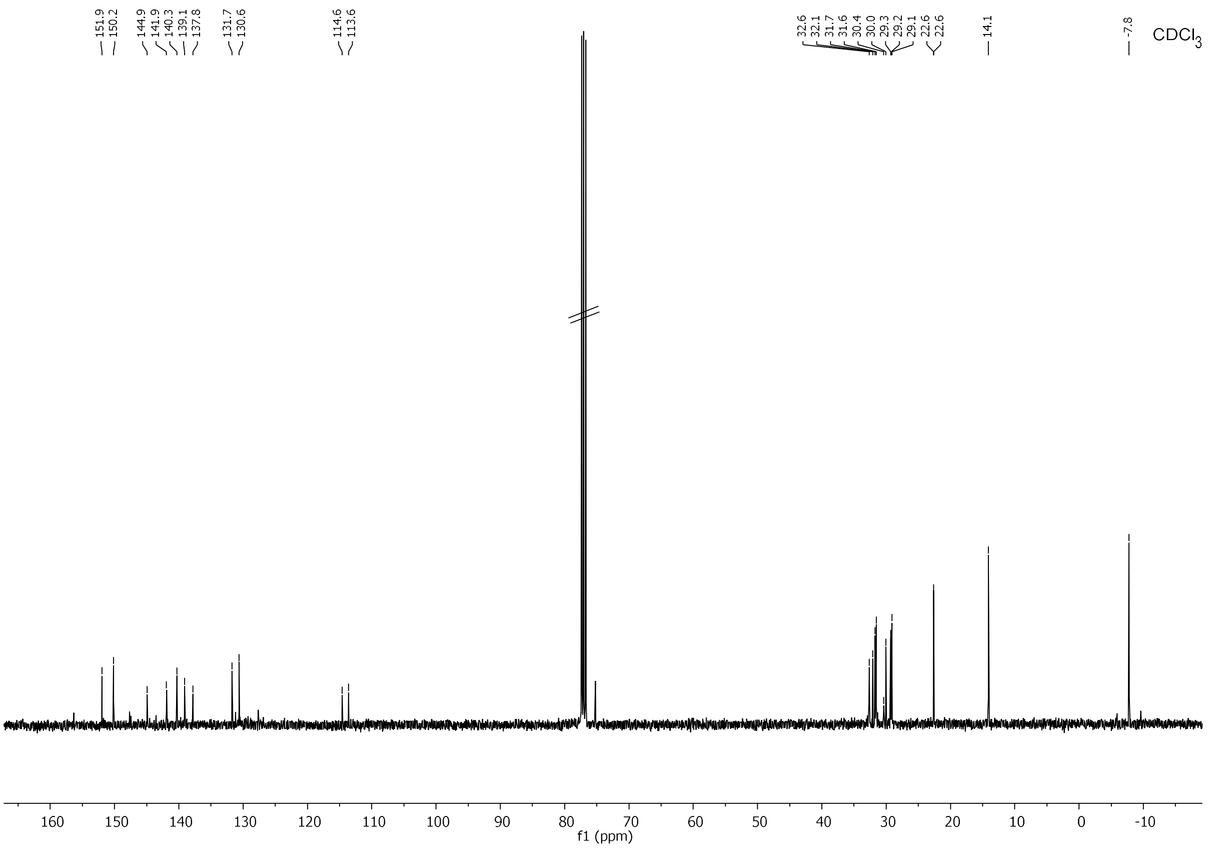

Figure S13b ^{13}C-NMR spectrum of **10** measured in $CDCl_3$.

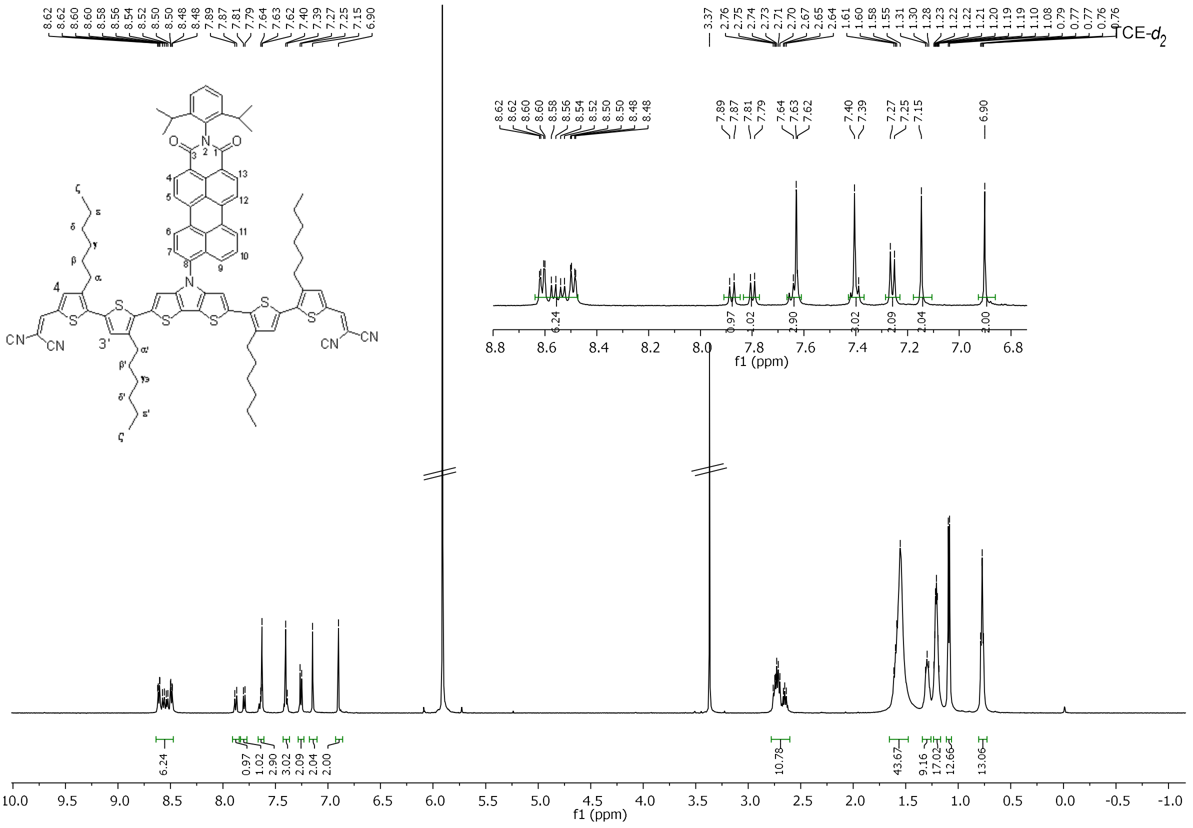

Figure S14a ^{1}H-NMR spectrum of **11** measured in TCE-d_2.

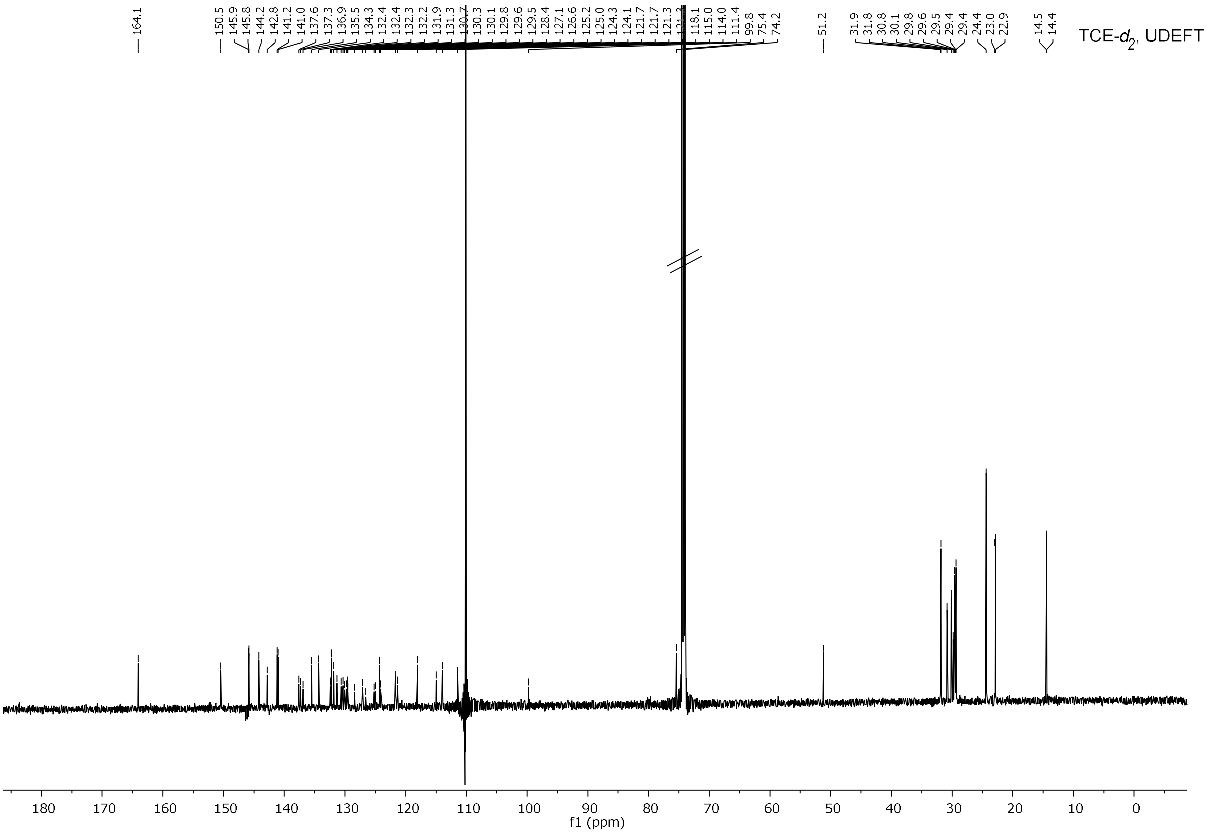

Figure S14b ^{13}C-NMR of spectrum of **11** measured in TCE-d_2.

5. High resolution mass spectra

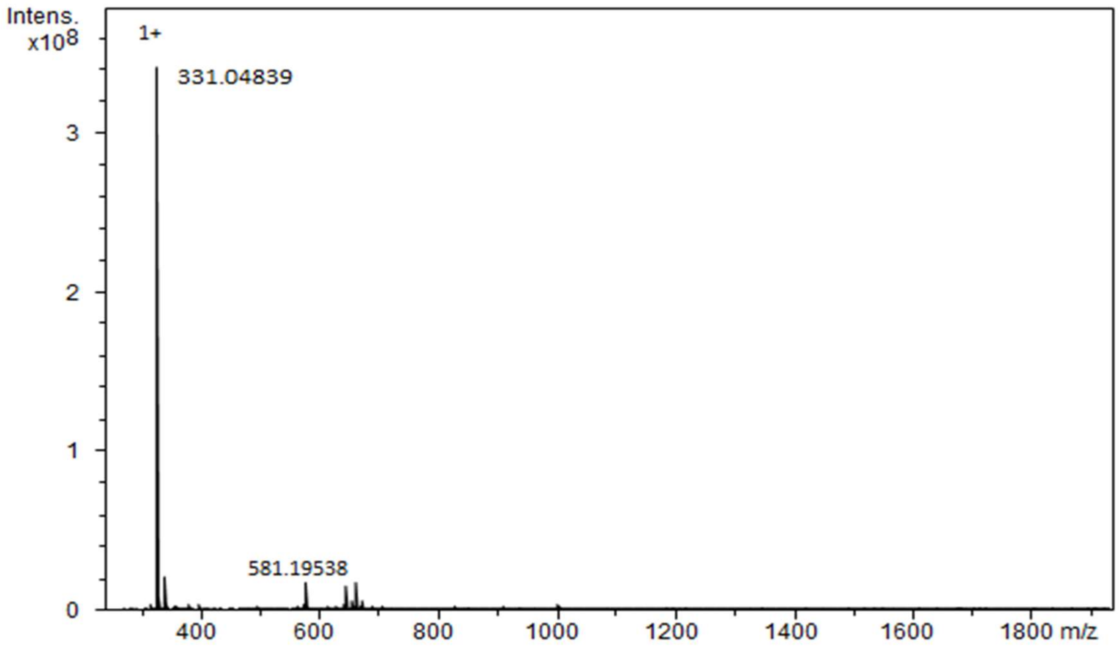

Figure S15 High resolution MALDI FTICR mass spectrum of **3f**.

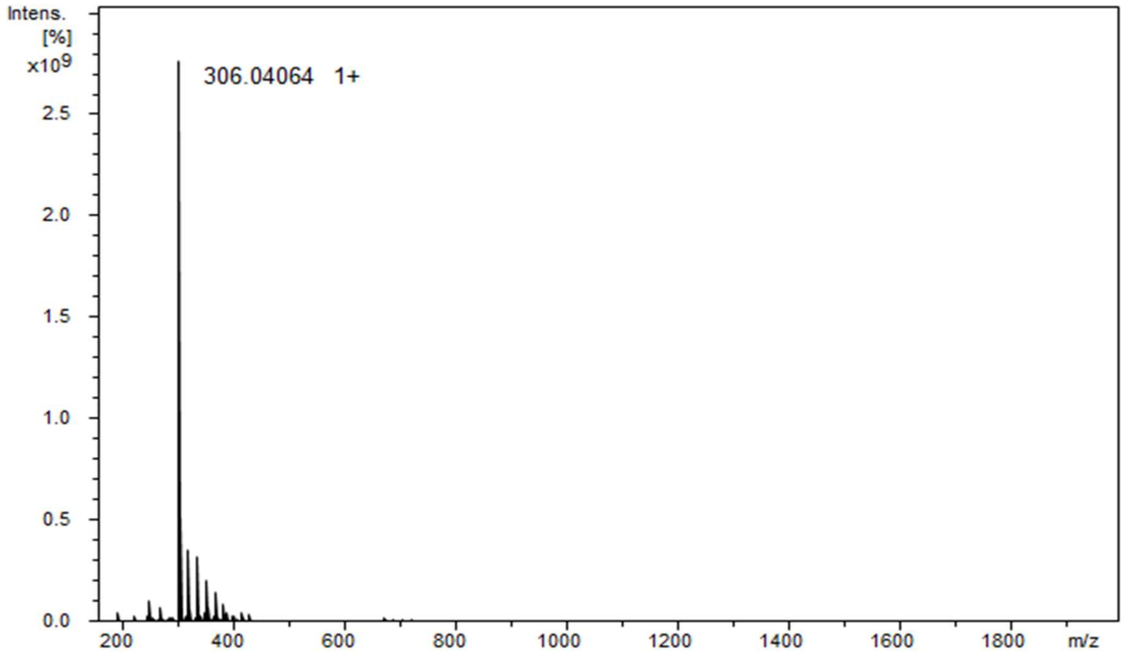

Figure S16 High resolution MALDI FTICR mass spectrum of **3g**.

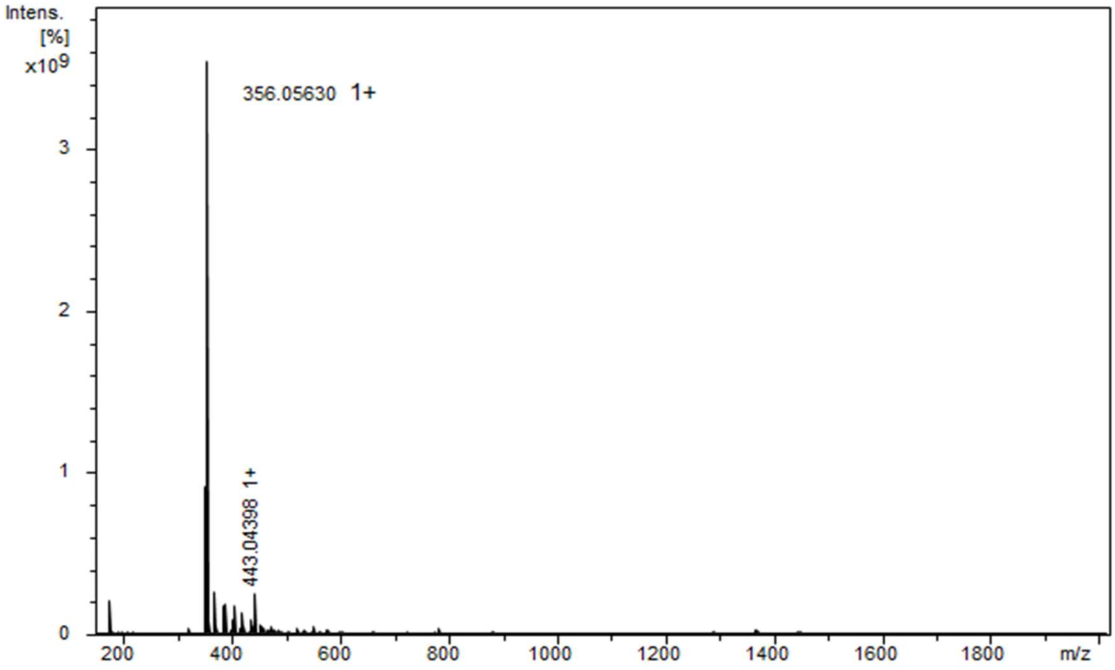

Figure S17 High resolution MALDI FTICR mass spectrum of **3h**.

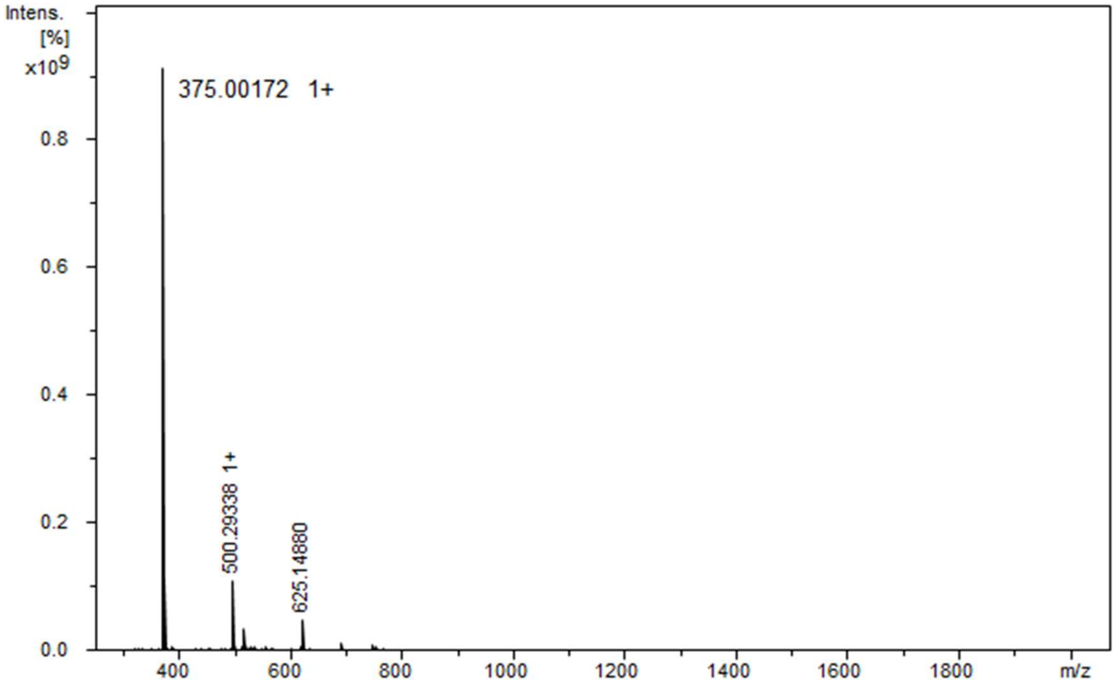

Figure S18 High resolution MALDI FTICR mass spectrum of **3i**.

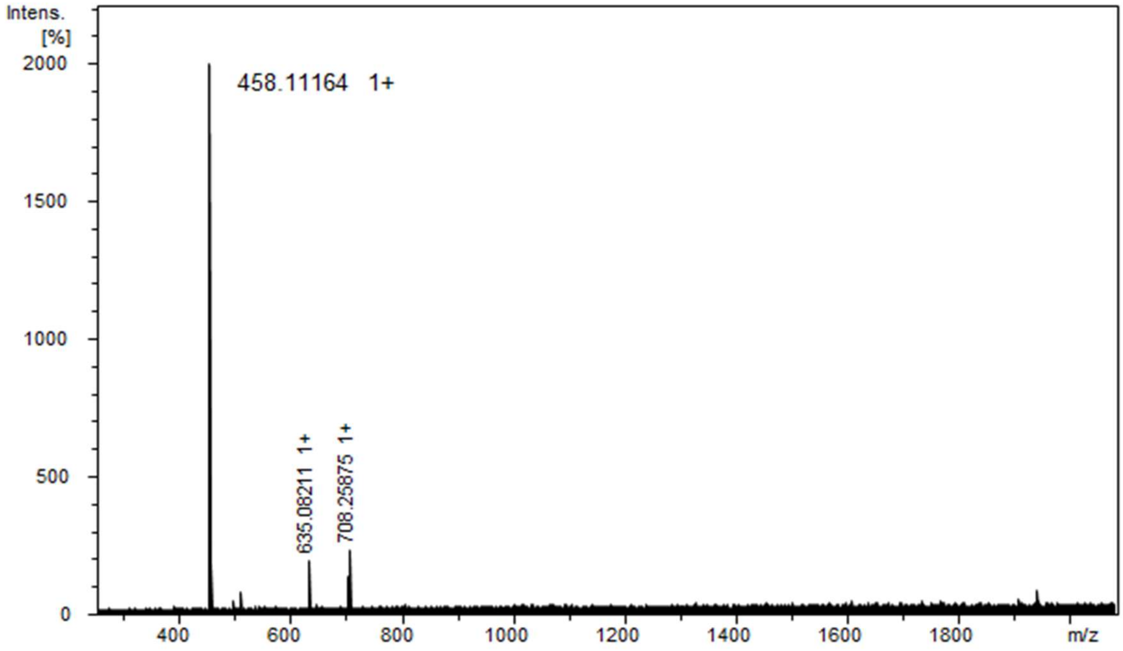

Figure S19 High resolution MALDI FTICR mass spectrum of **3j.**

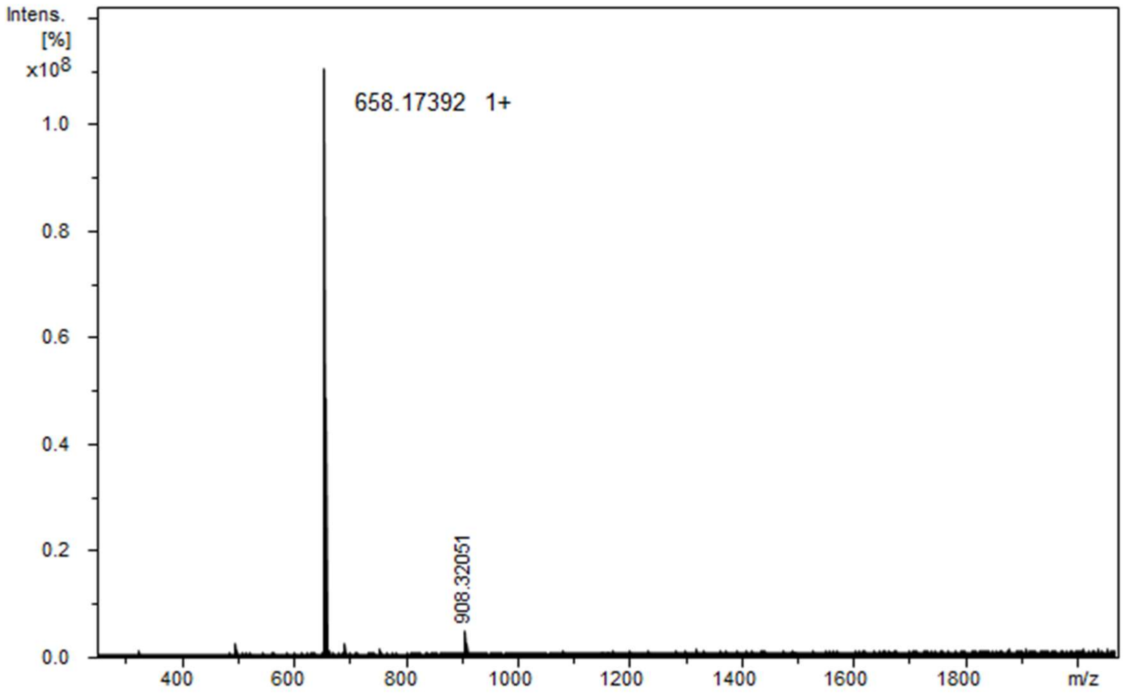

Figure S20 High resolution MALDI FTICR mass spectrum of **3k**.

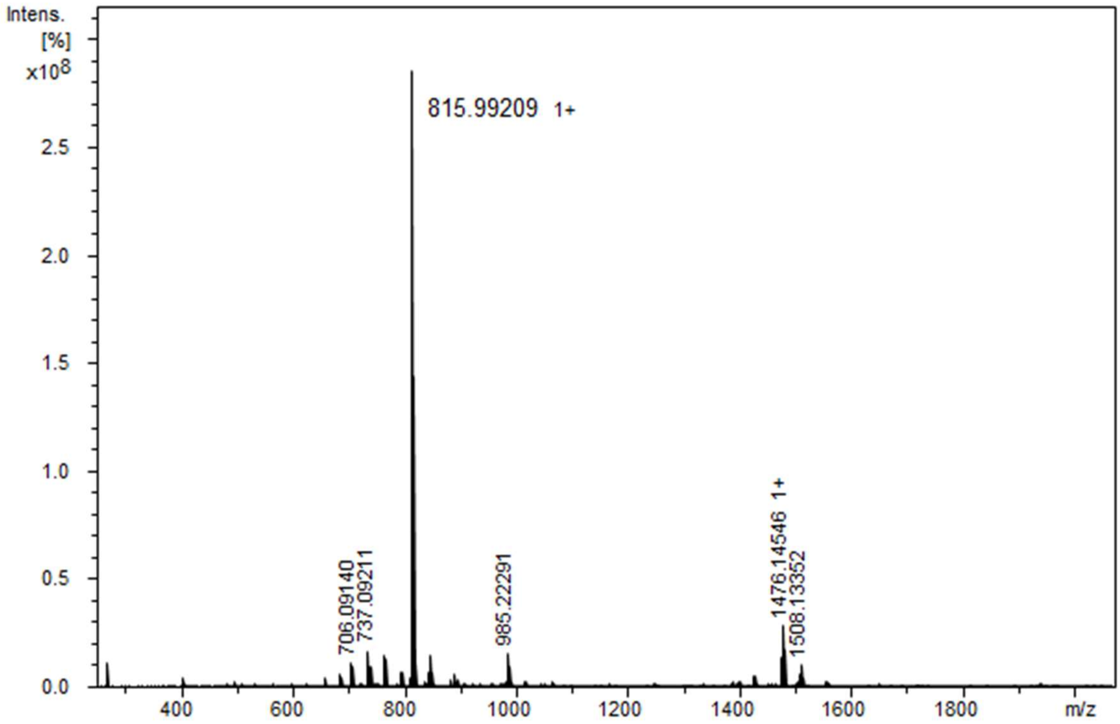

Figure S21 High resolution MALDI FTICR mass spectrum of **4**.

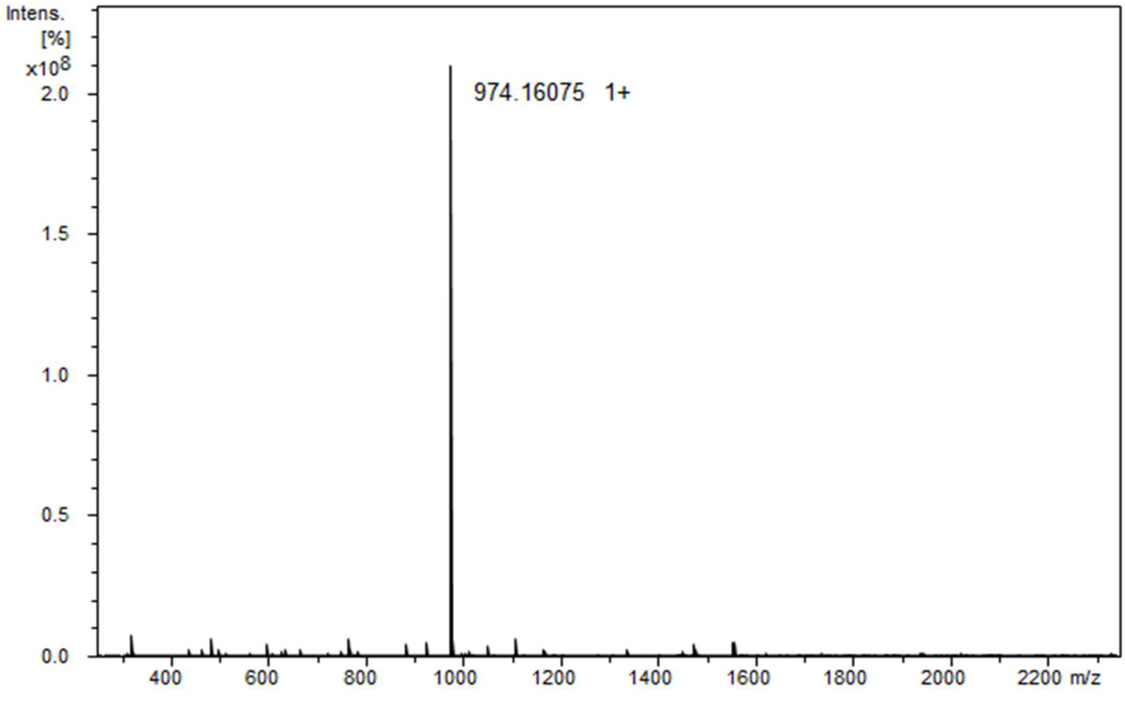

Figure S22 High resolution MALDI FTICR mass spectrum of **6**.

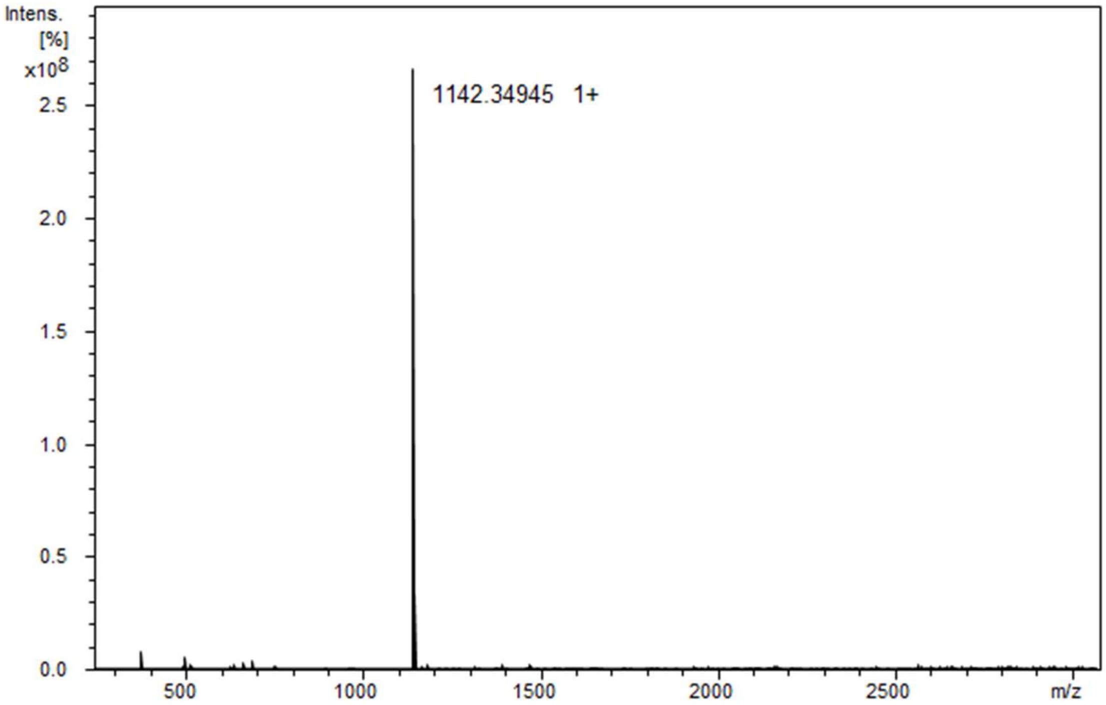

Figure S23 High resolution MALDI FTICR mass spectrum of **8**.

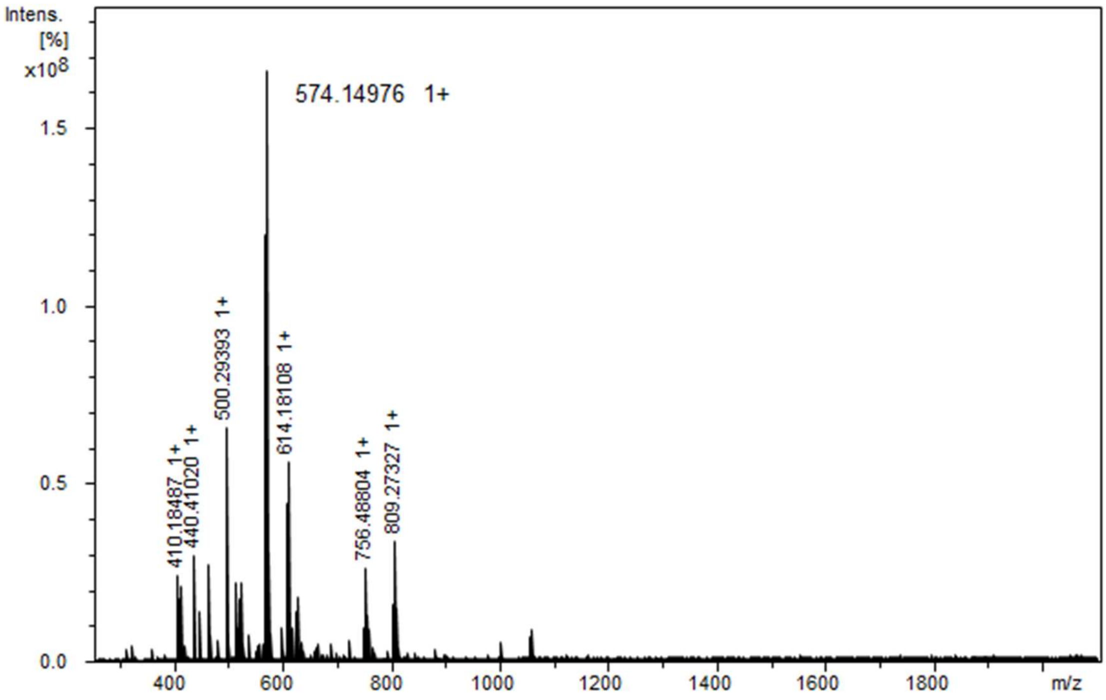

Figure S24 High resolution MALDI FTICR mass spectrum of **10**

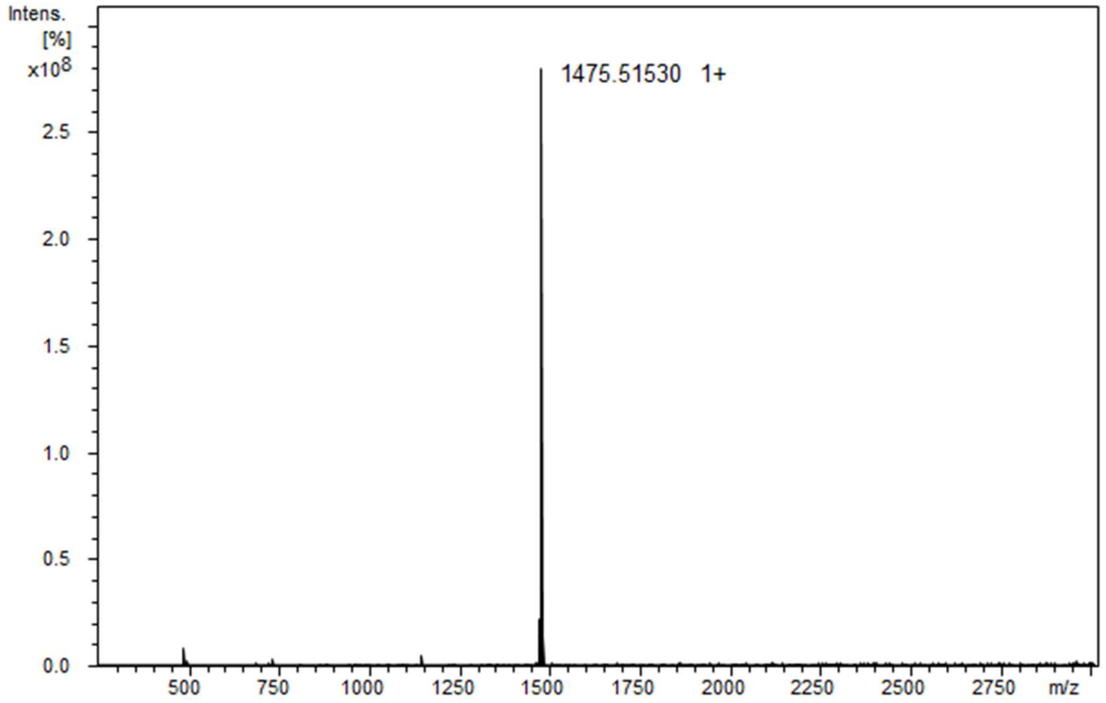

Figure S25 High resolution MALDI FTICR mass spectrum of **11**.

6. Cyclovoltammograms

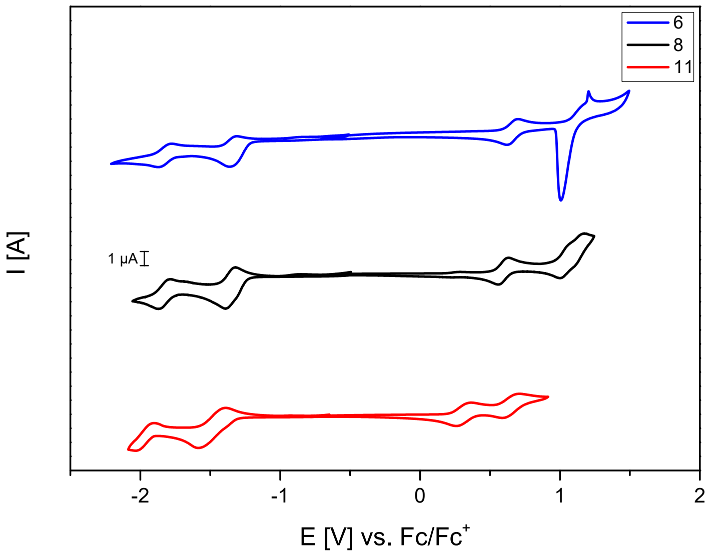

Figure S26: Cyclic voltammograms of D-A dyads **6** (c= 0,52 x10^{-3} Mol/L), **8** (c= 0.45 x 10^{-3} Mol/L)**, 11** (c= 0.63 x 10^{-3} Mol/L) in dichloromethane/tetrabutylammonium hexafluorophosphate (0.1M), 100 mV/s, r.t..

7. Photovoltaic properties

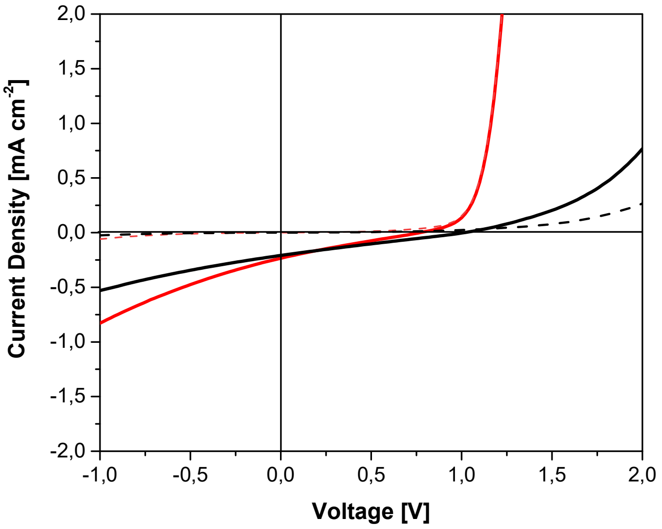

Figure S27: *J-V*-curves of PDCI-DTPs **8** (black curve) and **11** (red curve) in the standard device structure glass/ ITO/ PEDOT:PSS/ dyad **8** or **11**/ LiF/ Al, in the dark (dotted line) and under illumination (full line) at 100 mW/cm^2 AM1.5G light.

8. References

[1] C. Wessendorf, A. Perez-Rodriguez, J. Hanisch, A. Arndt, I. Ata, G. Schulz, A. Quintilla, P. Bäuerle, U. Lemmer, P. Wochner, E. Ahlswede, E. Barrena, *J. Mater. Chem. A*, **2016**, *4*, 2571-2580.

[2] M. J. Frisch, **2013**, Gaussian, Inc., Wallingford CT.

[3] a) A. D. Becke, *J. Chem. Phys.* **1993**, *98*, 5648-5652; b) C. Lee, W. Yang, R. G. Parr, *Phys. Rev. B: Condens. Matter* **1988**, *37*, 785-789.

[4] Y. Zhao, D. G. Truhlar, *Theor. Chem. Acc.* **2008**, *120*, 215-241.

[5] S. Förtsch, A. Vogt, P. Bäuerle, *J. Phys. Org. Chem.* **2017**, *30*:e3743.

[6] T. T. Do, H. D. Pham, S. Manzhos, J. M. Bell, P. Sonar, *ACS Appl. Mater. Interfaces* **2017**, *9*, 16967-16976.

[7] S. M. Dyar, E .A. Margulies, N. E. Horwitz, K. E. Brown, M. D. Krzyaniak, M. R. Wasielewski, *J. Phys. Chem .B.* **2015**, *119*, 13560-13569.

[8] M. Yuchao, W. Jianchang, G. Wei, Y. Ni, W. Lilei, M. Changqi, Patent CN 105646559, Jun 08, **2016**.

[9] N. Keller, D. Bessinger, S. Reuter, M. Calik, L. Ascherl, F.C. Hanusch, F. Auras, T. Bein, *J. Am. Chem. Soc.* **2017**, *139*, 8194-8199.

[10] D. R. Coulson, *Inorg. Synth.*, **1990**, *28*, 107-109.

[11] H.-L. Wong, C.-C. Ko, W. H. Lam, N. Zhu, V. W.-W. Yam, *Chem. Eur. J.* **2009**, *15*, 10005-10009.

[12] K. Nozaki, K. Takahashi, K. Nakano, T. Hiyama, H.-Z. Tang, M. Fujiki, S. Yamaguchi, K. Tamao, *Angew. Chem.* **2003**, *115*, 2097-2099; *Angew. Chem., Int. Ed.* **2003**, *42*, 2051-2053.
